# A Systematic Review and Meta-Analysis of Conditioned Pain Modulation in Children and Young People with Chronic Pain

**DOI:** 10.3390/children11111367

**Published:** 2024-11-11

**Authors:** Christina Liossi, Helen Laycock, Kanmani Radhakrishnan, Zara Hussain, Daniel Eric Schoth

**Affiliations:** 1 Pain Research Laboratory, School of Psychology, University of Southampton, Highfield, Southampton SO17 1BJ, UK; k.radhakrishnan@soton.ac.uk (K.R.); zrh1g21@soton.ac.uk (Z.H.); d.e.schoth@soton.ac.uk (D.E.S.); 2 Great Ormond Street Hospital for Children NHS Foundation Trust, London WC1N 3JH, UK; helen.laycock@doctors.org.uk

**Keywords:** paediatric chronic pain, conditioned pain modulation, quantitative sensory testing, systematic review, meta-analysis

## Abstract

Background/Objectives: Conditioned pain modulation (CPM) is a psychophysical experimental measure of the endogenous pain inhibitory pathway in humans, wherein one pain stimulus (the conditioning stimulus) is used to inhibit an individual’s perception of a second painful (test) stimulus. Research provides evidence of impaired endogenous inhibitory pain responses in adults with chronic pain. CPM is now increasingly applied in paediatric research and clinical practice. The primary aim of this systematic review was to examine the efficacy of CPM in paediatric chronic pain populations (6–24-year-olds) compared to pain-free children and young people (CYP). Methods: The protocol was registered on PROSPERO (CRD42020221927). A systematic search of seven databases was conducted from database inception to 20th June 2024. Study inclusion criteria were as follows: (i) recruited a sample of CYP aged 6 to 24 (inclusive) with chronic pain or who were pain-free; and (ii) applied a CPM paradigm comprising both a painful test and conditioning stimuli that were sufficiently detailed to allow for replication,(iii) adhered to a study design of randomised control trial, case control or cohort study, including cross-sectional or longitudinal; (iv) available in the English language. Study exclusion criteria were: (i) The CPM paradigm used a non-painful test or conditioning stimulus only; and (ii) was only available as an abstract, letter, poster, editorial, case report, or review with or without meta-analyses. Risk of bias was assessed using the Appraisal Tool for Cross Sectional Studies (AXIS). Meta-analyses were conducted in Comprehensive Meta Analysis 3.0 using random effects models to compare the overall CPM responses in CYP with chronic pain conditions to healthy control CYP. Results: Thirty-two studies were eligible for inclusion, six of which were included in one or more meta-analysis (*n* = 407 chronic pain, *n* = 205 control). Meta-analysis revealed significantly weaker CPM responses in CYP with a variety of chronic pain conditions compared to healthy controls (standardized mean difference (SMD) = 0.352), and significantly weaker CPM responses in CYP with abdominal pain conditions compared to healthy controls (SMD = 0.685). No significant difference in CPM response was found between CYP with migraine and healthy controls (SMD = −0.201). Conclusions: Variable results were found across individual studies, and the meta-analysis of the small number of eligible studies provides tentative evidence for impaired CPM in CYP with chronic pain compared to healthy controls. Further research is clearly needed. In particular, studies should present CPM results separately for different age groups, ethnic groups, and sexes, as these variables shape clinical pain responses.

## 1. Introduction

Chronic pain, defined as pain persisting or recurring for longer than three months [1], presents a complex challenge for modern healthcare systems. Although prevalence rates vary across reports and conditions [2,3,4], paediatric chronic pain is a significant global problem affecting approximately 1 in 5 children and young people (CYP) and can have devastating impacts on quality of life [5,6]. Paediatric chronic pain contributes to delays in social and cognitive development [7,8], disrupts family functioning [9], and increases the risk of adult disability [10,11,12].

**Figure 1:**
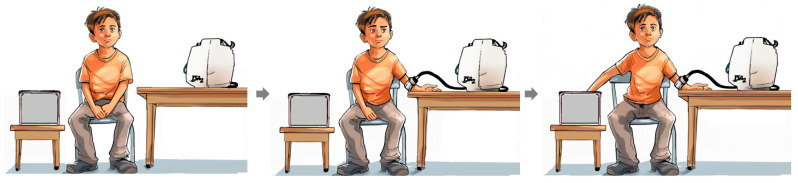
Graphical depiction of a typical conditioned pain modulation experimental paradigm using heat pain as test stimulus (administered to participant’s left forearm) and cold pain as conditioning stimulus (administered to participant’s right hand and wrist). From left to right: (i) participant is seated comfortably in a temperature- and sound-controlled environment; (ii) heat pain test stimulus is administered via thermode alone and heat pain threshold is recorded; (iii) heat pain test stimulus is administered again in the presence of the conditioning pain stimulus (cold water administered via the cold pressor test) and the heat pain threshold is recorded again. Figure created by an independent artist with the assistance of Midjourney version 5.

Paediatric chronic pain is a biopsychosocial phenomenon with a multifactorial aetiology; its treatment requires a comprehensive, personalised assessment and formulation [13], and interdisciplinary management [14]. Pain phenotyping via Quantitative Sensory Testing (QST) has been integrated into patients’ comprehensive biopsychosocial assessment, providing guidance on potentially effective or ineffective treatments, and is increasingly used in clinical care [15]. Conditioned pain modulation (CPM) is a psychophysical experimental measure of the endogenous pain inhibitory pathway in humans, which has the potential to become an additional phenotypic biomarker for pain and to serve as a guide for mechanism-based treatment in chronic pain. The term CPM was coined by Yarnitsky and colleagues, and has largely replaced previous terms such as diffuse noxious inhibitory control (DNIC) in the literature [16,17]. In a typical CPM paradigm, one pain stimulus (the conditioning stimulus) is used to inhibit an individual’s perception of a second painful (test) stimulus (see Figure 1). Within CPM protocols, test stimuli vary and may be applied sequentially or in parallel to a noxious conditioning stimulus [18,19].

CPM is commonly studied in adults with chronic pain, with evidence of significantly impaired endogenous inhibitory pain responses found in several reviews using meta-analysis. Lewis and colleagues [20] found impaired CPM in patients with mixed chronic pain conditions compared to pain-free controls, which were also significantly impaired in patients with specific conditions of fibromyalgia, irritable bowel syndrome, and headache, but not arthritis. Two recent reviews also reported impaired CPM in patients with irritable bowel syndrome compared to healthy controls [21,22]. One review without meta-analysis reported mixed results for impaired CPM in patients with chronic lower back pain, however [23], while another found very uncertain evidence for impaired CPM in people with traumatic or nonspecific neck pain [24].

The psychometric properties of CPM have also been extensively studied, as they are a critical prerequisite for its use in clinical care. Reliability has been found to be highly variable across studies, with inter-session reliability (tests repeated the same day) worse than intra-session (tests repeated different days) reliability, and pressure and cold pressor test being the test stimulus and conditioned stimulus, respectively, most consistently associated with good to excellent intra-session reliability in healthy volunteers and chronic pain patients [25]. Concurrent validity (i.e., the extent to which experimentally assessed CPM correlates with pain characteristics such as intensity, duration, painful area, and associated disability) in patients with different chronic pain conditions is questionable. Data from 1958 patients, providing 62 correlations, showed that the majority of results (69%) reported nonsignificant correlations between CPM efficiency and clinical pain characteristics. The remaining results, however, indicated a correlation between CPM reduction and worse clinical pain presentation. Multiple factors have been associated with better CPM in adults, including younger adult age [26,27], male gender, ovulatory phase, positive expectations, attention to the conditioning stimulus, and carrier of the 5-HTTLPR long allele [27]. A recent review found limited evidence that the CPM effect was significantly associated with psychological factors in patients with spinal pain, however [28].

Few studies have explored CPM in children and adolescents with chronic pain [29,30]. A better understanding of the functioning of descending pain inhibitory pathways in children may help to inform interventions aimed at enhancing central pain inhibition within a developmental framework [31]. Therefore, the primary objective of this systematic review and meta-analysis was to examine the efficacy of CPM in paediatric populations. Secondary objectives were to (i) assess the influence of experimental and demographic variables on CPM outcomes (pain intensity ratings, pain detection thresholds, pain tolerance, nociceptive withdrawal reflex) and (ii) evaluate the psychometric properties of CPM for children and young people.

## 2. Materials and Methods

This systematic review was conducted and reported in accordance with the Cochrane Handbook for Systematic Reviews of Interventions [32] and the Preferred Reporting Items for Systematic Reviews and Meta-Analyses (PRISMA) [33]. The protocol was registered on PROSPERO (CRD42020221927). No ethical approval was required.

### 2.1. Literature Search

The Cochrane Library (title, abstract, keywords), Scopus (article title, abstract, keywords), Web of Science (title), PsycINFO, MEDLINE, and CINAHL using EBSCOhost (title, abstract) and OpenGrey (main search field) databases were searched from database inception until 20 June 2024. The reference lists of all eligible studies were also manually reviewed. The search strategy comprised three blocks with the Boolean search command AND used between each block: (1) CPM (conditioned pain modulation OR conditioned pain OR pain* modulation OR CPM OR diffuse noxious inhibitory control OR DNIC OR heterotopic OR counterirritant); (2) population (girl* OR boy* OR child* OR teen* OR paediatric OR pediatric OR juvenile* OR youth* OR adolescen* OR young* OR school age* OR school child* OR schoolchild*); and (3) pain (chronic pain* OR pain OR idiopathic OR neuropathic OR CRPS OR complex regional pain OR musculoskeletal OR abdominal OR irritable bowel OR inflammatory bowel OR IBS OR IBD OR migraine OR headache OR arthritis OR fibromyalgia). The search results were imported into Endnote X9.3.3 and duplicates were removed through automatic and manual screening. Titles and abstracts were screened independently by two authors (DES and ZH) to identify potentially eligible studies. Full texts were checked for eligibility by DES and either CL or ZH.

### 2.2. Inclusion and Exclusion Criteria

Study inclusion criteria: (i) recruited a sample of CYP aged 6 to 24 (inclusive) with chronic pain or who were pain-free. In cases of studies recruiting samples with age ranges including individuals over 24 years of age, they were only eligible for inclusion if the data were presented separately for 6–24 year olds. (ii) applied a CPM paradigm comprising both a painful test and conditioning stimuli that were sufficiently detailed to allow for replication. Outcome measures (e.g., pain intensity) must have been tested prior to and post-administration of the conditioning stimulus. (iii) adhered to a study design of randomised control trial, case–control, or cohort study, including cross-sectional or longitudinal. (iv) available in the English language. Studies were excluded if: (i) the CPM paradigm used a non-painful test or conditioning stimulus only; (ii) was only available as an abstract, letter, poster, editorial, case report, or review with or without meta-analyses.

### 2.3. Data Extraction

Data from the eligible studies were extracted using a standardised form (Table S1). Data were extracted by either GLL, DES, HL, or ZH and checked for accuracy by CL or DES. Where missing data were evident, the study authors were contacted to request the data.

### 2.4. Study Quality Assessment

The quality of all studies was assessed by DES, with KR independently performing quality assessment for 30% of all studies using the Appraisal Tool for Cross Sectional Studies (AXIS) [34]. AXIS features 20 questions which are answered as either ‘yes’, ‘no’ or ‘don’t know’, along with spaces for comments on each judgement provided. Minor edits were made to the wording of three items; item 3 was expanded from asking whether sample size was justified to include whether adequate power was also achieved; item 9 was edited to increase the relevance of the question to CPM in paediatric populations specifically; and item 20 was edited to ask whether both ethical approval and consent/assent were obtained. The Cohen’s Kappa initial interrater reliability was 0.73 for DES and KR, indicating substantial agreement, and with full agreement achieved on all ratings after discussion.

### 2.5. Meta Analytic Procedures

Meta-analyses were conducted if data from two or more studies were available [35]. Analyses were conducted comparing the overall CPM response in CYP with chronic pain conditions to healthy control CYP using all available data from the studies. Further analyses were then conducted to explore these between-group differences for specific test stimuli modalities (i.e., heat pain and pressure pain) and specific diagnostic categories (i.e., chronic abdominal pain conditions and migraine). Standardised mean differences for between-groups comparisons, comparing CPM responses in children with chronic pain compared to healthy controls, were computed using group means, standard deviations, and random effect models in Comprehensive Meta Analysis (CMA) 3.0 [36] using random effect models [37,38]. Cochrane’s Q and the I^2^ statistic were used to assess study heterogeneity. A significant Cochrane’s Q is indicative of heterogeneity, whereas the I^2^ statistic describes the percentage of variability in effect estimates due to heterogeneity as opposed to sampling error [35,39]. One study repeated CPM testing on the same sample with both pressure and heat pain test stimuli [30]. Appropriate analyses were therefore conducted with data from each test stimulus independently, as it is not appropriate to include the same sample more than once in an analysis [35]. Due to the lack of available data, it was not possible to conduct within-groups analyses.

## 3. Results

### 3.1. Search Results

**Figure 2:**
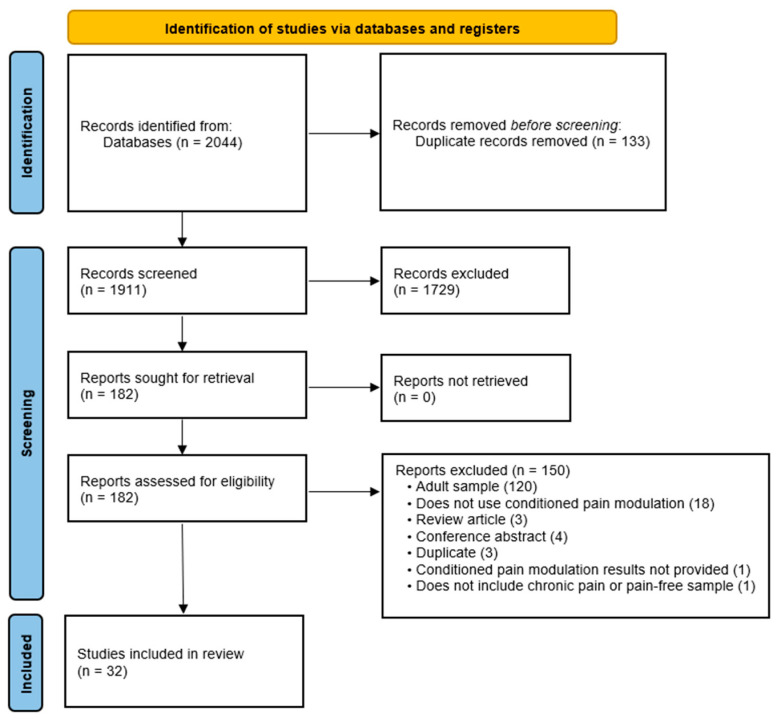
PRISMA 2020 flow of records for inclusion in the systematic review and meta-analysis of conditioned pain modulation in children and young people with chronic pain.

The full literature search process is presented in Figure 2. From the initial identification of 2044 records, thirty-two studies were included in this review, six of which were included in one or more meta-analyses.

### 3.2. Summary of Identified Studies

Fourteen studies were conducted in the USA; seven in Canada; three in Denmark; two in Portugal; and one each in Belgium, Italy, Spain, Sweden, Japan, and the United Kingdom. Twenty-one studies considered young people with pain. Specifically, three studies recruited participants with migraine; four studies with functional abdominal pain; one study with irritable bowel syndrome; two studies with patellofemoral pain; one study with hypermobility spectrum disorder or hypermobile Ehlers–Danlos syndrome; two studies with chronic back pain; one study with idiopathic scoliosis and chronic back pain; one study with neuropathic pain or complex region pain syndrome; two with mixed chronic pain conditions (e.g., musculoskeletal pain, abdominal pain); one exploring bullying involvement and mixed pain conditions (i.e., quantifying the prospective association between bullying and physical pain reporting); and three with widespread/mixed musculoskeletal pain. Of these studies, twelve also recruited a healthy control group, and two used data from a large birth cohort which also included data from pain-free participants. Eight studies recruited only healthy participants. Of the remaining three studies, one recruited prematurely born and full-term-born children, one study recruited adolescents with non-suicidal self-injury and healthy controls, and one recruited predominately healthy young women with a small percentage of participants with chronic pain.

Heat pain was used exclusively as a test stimulus in fifteen studies, with fourteen assessed via thermode and one via thermal grill. Of these studies, thirteen assessed heat pain intensity and three assessed heat pain threshold. Pressure pain was used exclusively as a test stimulus in sixteen studies, with ten assessed via algometer, four via cuff pressure algometer, and two via hydraulic piston connected to a computer-activated pump. Of these studies, eleven assessed pressure pain threshold, three assessed pressure pain threshold and tolerance, and two assessed pressure pain intensity. One study assessed both heat pain intensity via thermode and pressure pain threshold via algometer in separate conditions. Twenty-three studies used the cold pressor task as the conditioning stimulus, four used pressure pain via computerised cuff pressure algometer, three studies used hot water immersion, one study used heat pain via thermode, and one used pressure pain via sphygmomanometer.

### 3.3. Methodological Quality

**Figure 3:**
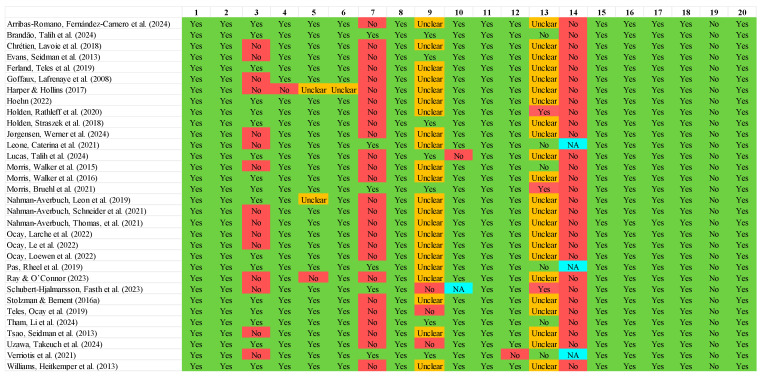
AXIS assessments for all eligible studies included in a systematic review and meta-analysis of conditioned pain modulation in children and adolescents. Note 1. Numbered items are as follows: 1. Were the aims/objectives of the study clear? 2. Was the study design appropriate for the stated aim(s)? 3. Was the sample size justified, with evidence of adequate statistical power? 4. Was the target/reference population clearly defined? (Is it clear who the research was about?) 5. Was the sample frame taken from an appropriate population base so that it closely represented the target/reference population under investigation? 6. Was the selection process likely to select subjects/participants that were representative of the target/reference population under investigation? 7. Were measures undertaken to address and categorise non-responders? (i.e., potential participants not responding to study invitation) 8. Were the risk factor and outcome variables measured appropriate to the aims of the study? 9. Were the CPM outcome variables measured correctly using instruments/measurements that had been trialled, piloted, or published previously in an equivalent paediatric sample? 10. Is it clear what was used to determine statistical significance and/or precision estimates? (e.g., *p*-values, confidence intervals). 11. Were the methods (including statistical methods) sufficiently described to enable them to be repeated? 12. Were the basic data adequately described? 13. Does the response rate raise concerns about non-response bias? 14. If appropriate, was information about non-responders described? 15. Were the results internally consistent? 16. Were the results presented for all the analyses described in the methods? 17. Were the authors’ discussions and conclusions justified by the results? 18. Were the limitations of the study discussed? 19. Were there any funding sources or conflicts of interest that may have affected the authors’ interpretation of the results? 20. Was ethical approval granted and consent/assent of participants attained? Note 2. Green signifies a favourable assessment, red signifies an unfavourable assessment, orange signifies the answer is unclear from the information provided in the study article, and blue signifies the item is not applicable (NA).

Methodological quality assessment ratings are presented in Figure 3. All studies adequately reported the aims/objectives (item 1); used an appropriate study design (item 2); included outcome variables appropriate to the aims of the study (item 8); provided a detailed description of methods and statistical methods (item 11); reported results that were judged to be internally consistent (item 15); presented results for all the analyses described in the methods (item 16); provided detailed and justified discussion of their results (item 17); commented upon study limitations (item 18); did not raise concerns regarding conflicts of interest or funding statements (item 19); and obtained ethical approval and adequately obtained participant consent/assent (item 20). All but one study clearly defined the target population (item 4), all but one study clearly reported the criteria for statistical significance, and all but one study adequately presented the basic data (item 12). Twenty-nine studies had a sample frame taken from an appropriate population base so that it closely represented the target population (item 5), and for all but one study, the selection process was likely to select participants that were representative of the target population (item 6). For thirty studies, the criteria for determining statistical significance were clear (item 10).

In contrast to these general strengths, some limitations are notable. Only 17 studies reported a power calculation with evidence of adequate statistical power (item 3). Only six studies took measures to address and categorise non-responders (item 7), no study provided information on non-responders (item 14), and for only six studies was it clear that the response rate did not raise concerns about non-response bias (concerns were evident for three studies, with the remainder unknown; item 13). Only seven studies clearly reported that the CPM outcome variables had been previously trialled, piloted, or published in an equivalent paediatric sample specifically (item 9).

### 3.4. Narrative Summary

A summary of the main CPM details for each study is provided in Appendix A. Unless otherwise stated below, studies recruited both males and females. Thirteen studies compared CPM responses in children with pain to healthy children [30,40,41,42,43,44,45,46,47,48,49,50,51]. Two studies explored CPM in children with functional abdominal pain (FAP) and healthy controls. Morris and colleagues [44] found children with FAP to show weaker CPM responses than healthy controls, while Pas and colleagues [45] found children with FAP to show a significantly lower CPM response than healthy children. Williams and colleagues [46] explored CPM in girls with irritable bowel syndrome (IBS) and healthy girls. A significant difference between the two groups was observed, with CPM being deficient in girls with IBS compared to healthy controls.

Chrétien and colleagues [43] explored CPM in girls with mixed chronic pain conditions and healthy adolescent girls. Healthy girls showed a significant reduction in heat pain intensity following a cold pressor task compared to ratings taken prior to the task, with no significant decrease in girls with chronic pain. CPM magnitude was, therefore, significantly greater in healthy girls compared to those with chronic pain. Ocay and colleagues [41] recruited children with chronic musculoskeletal pain and healthy controls. Overall, CPM efficiency was significantly weaker in children with musculoskeletal pain compared to healthy controls. However, the authors concluded that adolescents with chronic musculoskeletal pain are a heterogenous population, as distinct subgroups were identified including patients who did and did not display optimal CPM efficiency. Ocay and colleagues [47] regrouped data from multiple studies recruiting children and adolescents with mixed chronic pain conditions and healthy controls. Mean CPM efficiency did not significantly differ between those with chronic pain and healthy controls. Cluster analysis was performed, which revealed heterogeneity amongst patients in their responses to CPM.

Two studies from Nahman-Averbuch and colleagues explored CPM in children with migraine and healthy controls. The first study [30] also recruited a sample of healthy children with a family history of migraine. No significant differences were found in CPM response between groups when either heat pain or pressure pain tests were used. The second study [40] also reported no significant differences in CPM responses in children with migraine compared to healthy controls. Two studies from Holden and colleagues explored CPM in young people with patellofemoral pain. In the first study [49], females with patellofemoral pain had significantly impaired CPM (assessed via pressure tolerance threshold) compared to those recovered from patellofemoral pain, but no differences compared to healthy controls. No differences were found in CPM when pressure pain threshold was considered. The second study [48] delivered an intervention to all participants consisting of activity modification, education, and graded return to sport. At baseline, the healthy control group had a significant increase in pain detection threshold during the painful conditioning stimulus compared to without conditioning. No difference was found for the patellofemoral pain group, indicating no efficient CPM response, which also did not change over time in response to the intervention.

Brandão and colleagues [51] analysed data from the Generation XXI birth cohort in Portugal, collecting pain history at 7, 10, and 13 years. For adolescents with musculoskeletal pain at 13 plus a history of pain, the CPM effect was slightly increased for the pain detection threshold and pain tolerance compared to other participants, although this effect was not statistically significant. Impaired CPM was not detected among adolescents with musculoskeletal pain. The same pattern of results was found for adolescents with musculoskeletal pain at seven and ten with a history of pain. Schubert-Hjalmarsson and colleagues [42] conducted a feasibility study exploring central sensitization in adolescents with hypermobility spectrum disorder or hypermobile Ehlers–Danlos syndrome. Only descriptive statistics were reported, which showed a similar CPM outcome in adolescent patients and healthy adolescents. Jørgensen and colleagues [50] assessed somatosensory profiles in children and adolescents with and without cerebral palsy and with and without chronic pain. Across all participants, the pressure pain threshold consistently increased by a median of approximately 30 kPa from unconditioned to conditioned test stimuli. No other effects were found.

Seven studies explored CPM responses in a single group of young people with chronic pain [29,52,53,54,55,56,57] with may reporting variability in CPM responses. Ocay and colleagues [53] recruited adolescents with chronic back pain. CPM efficiency was found to be optimal in 51.5% of adolescents, suboptimal in 22.7% of adolescents, and inefficient in 25.8% of adolescents. Teles and colleagues [54] recruited children with idiopathic scoliosis and chronic back pain. An efficient pain inhibitory response was shown by 51.1% of children, while 21.3% had sub-optimal CPM and 27.7% had inefficient CPM. Ferland and colleagues [56] recruited children and adolescents with chronic back pain, finding no significant differences in CPM response between males and females. The authors investigated the role of blood monoamines as biomarkers of CPM efficiency.

Verriotis and colleagues [55] explored CPM responses in children with neuropathic pain, which also revealed a spectrum of responses. Specifically, CPM was found to be inhibitory in 54% of children and facilitatory in 14% of children. Morris and colleagues [52] recruited children with FAP, who were randomised to receive either an internet-delivered program of cognitive behaviour therapy (CBT) or pain education. Children were also categorised at baseline into High Pain Dysfunctional, High Pain Adaptive, and Low Pain Adaptive groups, with no significant differences between these groups found for CPM response. The results also showed that beyond any effects of the intervention, pain-related interference declined significantly over time for children with stronger baseline CPM. Tham and colleagues [29] recruited adolescents with FAP, finding that higher CPM (i.e., more efficient CPM) was significantly correlated with lower abdominal pain intensity. When adjusted for age and sex, this was no longer significant, although it was marginally associated with increased pain interreference. Nahman-Averbuch and colleagues [57] recruited children with migraine who received CBT (this sample was also included in a subsequent study by the authors, as discussed above [40]. A significant CPM response was seen in children before CBT, although the authors note that the effect was variable.

Eleven studies explored CPM responses in healthy children only [31,58,59,60,61,62,63,64,65,66,67]. Evans and colleagues [58] explored sex differences in the association between maternal anxiety about pain and children’s CPM responses. For boys, but not girls, higher material anxiety was significantly associated with lower CPM (i.e., less pain inhibition), which remained significant even after accounting for the effects of child age and maternal general psychological distress. Tsao and colleagues [31] also explored the effects of sex and age on CPM responses in healthy children. A significant CPM effect was revealed for the whole sample, although younger children (8–11 years) showed significantly less CPM than adolescents (12–17 years). No differences in CPM were found between boys and girls. Hoehn and colleagues [67] explored the effects of age on CPM in children aged 6 to 12. Significant CPM effects were observed for the majority of the sample, although no significant effect of age was observed for CPM magnitude. The authors conclude that CPM is evident in children as young as 6. Morris and colleagues [59] explored race effects on CPM, finding stronger CPM effects in African-American than Non-Hispanic White children. Stolzman and Bement [60] assessed the impact of body composition on CPM responses in adolescents. CPM responses were found to be similar across sites (nailbed vs. deltoid), weight status (normal vs. overweight/obese), and between boys and girls. CPM at the deltoid was, however, positively associated with left arm lean mass, while CPM at the nailbed was positively associated with physical activity levels.

Ray and O’Connor [62] explored the effects of yoga and slow breathing on endogenous pain modulation in young females. Although most participants were healthy, 7.1% of participants in one of the four groups reported chronic pain. The results showed no significant effects of yoga or slow breathing on CPM efficiency. Leone and colleagues [63] recruited adolescents with non-suicidal self-injury (NSSI) and healthy controls. Adolescents with NSSI showed deficient CPM compared to healthy adolescents. Goffaux and colleagues [61] recruited children who were split into three groups based on their birth status: term-born, born preterm and exposed to numerous painful interventions, and born preterm and exposed to few painful interventions. Cold pressor conditioning pain was found to significantly reduce test heat pain intensity for the full-term and low-pain pre-term groups, with strong inhibitory responses. The high-pain pre-term group, however, demonstrated a complete absence of CPM effect. Harper and Hollins [64] recruited young adults in order to explore the underlying neural mechanisms of the thermal grill illusion (TGI), which refers to a perception of burning heat and sometimes pain that is experienced as a result of the simultaneous application of innocuous warm and cool stimuli to the skin. TGI was compared to a noxious heat condition, with the results showing that CPM produced significant and comparable reductions in pain, unpleasantness, and perceived heat in both conditions.

Uzawa and colleagues [65] examined sex differences in CPM effects in young individuals, along with associations between CPM effects and autonomic activities. No significant differences in CPM indices were found between males and females. Activity in sympathetic and parasympathetic nervous systems was related significantly to CPM effects across all participants, although descending pain modulations in females might have been more associated with autonomic activities in females than males. Lucas and colleagues [66] analysed data from the Generation XXI birth cohort in Portugal, aiming to quantify potential associations between bullying and physical pain in adolescents. Participants were classified as ‘victim only’, ‘both victim and aggressor’, ‘aggressor only’, or ‘not involved’. Adolescents classified as ‘aggressors only’ had more efficient CPM for pressure detection thresholds compared to those not involved in bullying. Further to these studies with healthy participants only, Arribas-Romano and colleagues [68] explored CPM and psychological factors in young people with chronic neck pain and healthy controls. The former group’s age extended beyond the present review’s inclusion criteria, however, and therefore, only the results of the pain-free individuals are considered here. The results showed that the reported pain intensity to the test stimulus was lower during the presence of the conditioning stimulus than at baseline for healthy children.

### 3.5. Psychometric Properties of Outcome Measures

A detailed summary of the outcome measures used and their reported psychometric properties for all studies included in this review is presented in Table S2.

Six studies recruited a sample of children with chronic pain alongside a sample of healthy controls and provided sufficient data for inclusion in the meta-analysis of CPM response in CYP with chronic pain compared to healthy controls [30,40,41,43,45,46]. Three studies used heat pain as the test stimulus [41,43,46], two used pressure pain as the test stimulus [40,45], and one repeated CPM testing with both pressure and heat pain test stimuli [30]. All studies used the cold pressor task as the conditioning stimulus. Insufficient data were available to address the two secondary objectives (i.e., the influence of individual differences and demographic variables on CPM outcomes and the psychometric properties of CPM for CYP) via meta-analytic techniques.

### 3.6. Meta-Analysis of CPM Response in CYP with Chronic Pain Compared to Healthy Controls

Between-groups comparison using the heat test stimulus data from Nahman-Averbuch and colleagues [30] revealed significantly weaker CPM responses in children with chronic pain compared to healthy controls (Analysis 1, *k* = 6, chronic pain *n* = 407, control *n* = 205, *Z* = 2.434, SMD = 0.352 (95% CI = 0.069, 0.636), *p* = 0.015. Heterogeneity: Q = 9.501, *p* = 0.091, I^2^ = 47.37%). The analysis was repeated with pressure pain test stimulus data from Nahman-Averbuch and colleagues [30], which again revealed significantly weaker CPM responses in children with chronic pain compared to healthy controls (Analysis 2, *k* = 6, chronic pain *n* = 407, control *n* = 205, *Z* = 2.079, SMD = 0.325 (95% CI = 0.019, 0.631), *p* = 0.038. Heterogeneity: Q = 10.998, *p* = 0.051, I^2^ = 54.54%).

Analysis with heat pain test stimuli data only [30,41,43,46] revealed significantly weaker CPM responses in children with chronic pain compared to healthy controls (Analysis 3, *k* = 4, chronic pain *n* = 350, control *n* = 149, *Z* = 2.702, SMD = 0.360 (95% CI = 0.099, 0.621), *p* = 0.007. Heterogeneity: Q = 3.740, *p* = 0.291, I^2^ = 19.79%). Analysis with pressure pain test stimuli only [30,40,45] revealed no significant difference in CPM responses between children with chronic pain compared to healthy controls (Analysis 4, *k* = 3, chronic pain *n* = 76, control *n* = 84, *Z* = 0.353, SMD = 0.111 (95% CI = −0.505, 0.728), *p* = 0.724. Heterogeneity: Q = 7.322, *p* = 0.026, I^2^ = 72.69%).

Analyses were also conducted where possible for specific diagnostic categories. Children with abdominal pain conditions showed significantly weaker CPM responses than healthy controls [45,46] (Analysis 5, *k* = 2, chronic pain *n* = 60, control *n* = 52, *Z* = 3.507, SMD = 0.685 (95% CI = 0.302, 1.067), *p* < 0.001. Heterogeneity: Q = 0.020, *p* = 0.889, I^2^ = 0%). Different test stimuli were used in these two studies; Pas and colleagues used pressure pain stimuli, and Williams and colleagues used heat pain stimuli. No significant difference in CPM response was found between children with migraine and healthy controls with the pressure pain test stimuli used [30,40] (Analysis 6, *k* = 2, chronic pain *n* = 38, control *n* = 48, *Z* = −0.918, SMD = −0.201 (95% CI = −0.629, 0.228), *p* = 0.358. Heterogeneity: Q = 0.166, *p* = 0.684, I^2^ = 0%). This analysis was repeated with the heat pain test stimulus from Nahman-Averbuch and colleagues [30], with the result remaining non-significant (Analysis 7, *k* = 2, chronic pain *n* = 38, control *n* = 48, *Z* = −0.440, SMD = −0.096 (95% CI = −0.524, 0.332), *p* = 0.660. Heterogeneity: Q = 0.720, *p* = 0.396, I^2^ = 0%). Forest plots can be found in Appendix B.

### 3.7. The Influence of Experimental and Demographic Variables on CPM Outcomes

Four studies included in this present review explored potential differences in CPM effects between boys and girls, with none reporting significant differences. Evans and colleagues [58] reported CPM magnitudes of boys and girls of 1.46 (SD = 2.0) and −1.51 (SD = 2.3), respectively. Tsao and colleagues [31] reported CPM magnitudes of boys and girls of −1.48 (SD = 2.0) and −1.58 (SD = 2.2), respectively. Ferland and colleagues [56] reported no differences between genders (*p* = 0.878), and neither did Stolzman and Bement [60] (*p* = 0.30). Two included studies explored the influence of age on CPM effects. One study found significantly less CPM in younger children aged 8–11 years (mean absolute CPM −0.98, SD = 2.4) than adolescents aged 12–17 years (mean absolute CPM −1.84, SD = 1.9) [31], while another did not find a significant main effect of age on CPM responses (*p* = 0.439) [67]. Only one study explored potential differences in CPM effects between different ethnic groups, reporting significantly stronger CPM in African-American children than Non-Hispanic White children (*p* = 0.02) [59].

### 3.8. Psychometric Properties of CPM 

Only two studies included in the present review evaluated the reliability of CPM, with both reporting excellent results. Hoehn and colleagues [67] reported a Cronbach’s alpha of 0.95 between two baseline tests of pressure pain threshold assessed minutes apart in healthy children (mean age 9.05 years, SD = 1.84). Verriotis and colleagues [55] reported an intraclass coefficient of 0.94 for three repeated pressure pain threshold measurements performed minutes apart in adolescents with neuropathic pain and adolescents with complex regional pain syndrome (median age 14.9 years, IQR = 12.9–16.1).

## 4. Discussion

The primary objective of this systematic review and meta-analysis was to examine the efficacy of CPM in CYP with chronic pain compared to pain-free CYP. The meta-analysis revealed significantly weaker CPM responses in CYP with chronic pain compared to healthy controls. Variation in the results is apparent, however, with sub-analyses showing that the CPM effect was significantly weaker in those with abdominal pain conditions compared to healthy controls, although no significant differences were found between those with migraine and controls. Sub-analyses revealed significantly weaker CPM responses in CYP with chronic pain compared to healthy controls when heat pain test stimuli were used, but not when pressure pain test stimuli were used. The secondary objectives were to assess the influence of experimental and demographic variables on CPM outcomes and evaluate the psychometric properties of CPM for CYP. Due to the limited number of studies in the meta-analyses, we were unable to statistically explore the impact of demographic variables on CPM effects. An assessment of psychometric properties for all outcome measures showed that no study reported any such properties for pain intensity, unpleasantness, or sensations during CPM.

The overall meta-analytic results in this review are broadly consistent with those reported in the adult meta-analytic literature, which has found evidence of impaired endogenous inhibitory pain responses in individuals with chronic pain [20,21,22]. However, it is important to note that results vary, and CPM may not manifest uniformly across all chronic pain conditions or under all testing conditions. The lack of CPM impairments in CYP with migraine in the present review reflects the inconsistency of CPM in adult migraine patients. A number of studies have found no evidence of impaired CPM in adults with migraine compared to controls (e.g., [69,70]), while another found evidence that the nociceptive flexion reflex was facilitated rather than inhibited during and after the cold pressor test as a conditioning stimulus [71]. Interestingly, Nahman-Averbuch and colleagues [72] reported no differences between patients with migraine and healthy controls on the first CPM trial, but did report less efficient CPM inhibition in patients across three further trials. The same lead author conducted the two paediatric studies included in the present review, although in these studies, only a single CPM trial was included [30,40]. This suggests that specific aspects of the CPM methodology are important considerations when exploring such effects. Furthermore, several studies which have shown impaired CPM in migraine have reported this for trigeminal pain specifically [73,74], highlighting site location in such CPM research as another important consideration.

While there currently appears to be a body of evidence emerging in CYP with chronic pain suggesting lowered CPM compared to healthy CYP, the utility of CPM in other paediatric populations is currently unclear. For example, in adults, a growing body of research has explored the links between CPM and exercise-induced hypoalgesia, with research suggesting CPM predicts greater exercise-induced hypoalgesia in healthy individuals [75] and patients with chronic musculoskeletal pain [76]. Areas such as this remain to be explored in CYP, although, considering the importance of exercise in managing chronic pain in young people [77,78], this is a key area for future research.

CPM methodology varied between the studies included in this systematic review, and in many cases the rationale for deciding upon CPM parameters was not provided. For example, the decision to use heat pain or pressure pain as a test stimulus was split approximately evenly across studies, although, as noted above, sub-analyses showed significantly weaker CPM responses in CYP with chronic pain compared to healthy controls when heat pain test stimuli were used, but not when pressure pain test stimuli were used. The use of both forms of stimuli has been deemed appropriate for exploring experimental pain in CYP [79,80], although it has been suggested that different CPM modalities may engage different inhibitory mechanisms [30]. Further research is clearly warranted exploring the importance of CPM parameters such as the form of test pain stimulus, which is further highlighted by recent animal research suggesting that noxious mechanical and thermal stimuli are associated with different temporal order processing in the cortex [81]. It should also be noted that a recent study with adults reported a stronger inhibitory CPM effect using a pressure-based paradigm compared to a heat-based paradigm, but only for healthy individuals and not patients with chronic pain [82]. Research exploring differences in CPM responses across the lifespan is needed.

A detailed review of the psychometric properties of individual difference outcome measures was performed (Table S2). A wide range of individual difference variables was assessed across the eligible studies, and variation was often noted in the specific measures used. Only a limited number of studies reported psychometric properties for the specific samples recruited, although it was more common for authors to report the properties available in the broader literature as justification for their choice of measure. It has been argued that reliability should be estimated and reported for each administration of any particular instrument, as reliabilities can vary across settings, populations, and administrations of the measure [83]. The American Psychological Association [84] also emphasises the importance of estimating and reporting reliability coefficients for the scores analysed in each individual study, including internal consistency, interrater reliability, and test–retest coefficients, where applicable. Within the present review, only internal consistency was reported. This further highlights the lack of attention given by researchers to this important aspect of research, which is relatively straightforward and quick to analyse and report.

An assessment of the methodological quality of each study was conducted, with various strengths noted. In particular, all studies stated their aims/objectives clearly, used appropriate designs, and presented internally consistent results. Nevertheless, only seven studies explicitly stated that they had used a CPM protocol or procedure that had been trialled, piloted, or published previously in an equivalent paediatric population. Another notable limitation of this body of literature was that only 17 studies reported the results of a power calculation or clearly justified their sample size. While sample sizes varied across studies, the possibility remains that certain studies may have been underpowered to detect significant effects should they exist in the population. A further limitation of note is that only six studies took appropriate measures to address and categorise non-responders (i.e., eligible individuals who for various reasons were not recruited or declined participation). There may be multiple reasons for non-response or participation in research [85], and of course, with certain study designs, it is simply not possible to ascertain such reasons (e.g., recruiting via advertisements). When patients are recruited from medical clinics, however, it would be informative for researchers to provide reasons for non-participation, which could include current levels of pain and disability. As such, non-responders may differ from responders in key characteristics, and therefore, results obtained from the latter may not be wholly generalisable to the former. Further to these limitations, it is also notable that very few studies explored the reliability of CPM. Improvements are needed in the computation and reporting of reliability statistics in paediatric CPM research, including test–retest coefficients and interrater reliability where applicable. We are also in agreement with Nuwailati and colleagues [25] that investigation is needed to explore whether low inter-session reliability is due to dynamic changes in endogenous inhibition or limitations in methodology.

A notable strength of the present review is the above-mentioned assessment of methodological quality. A limitation was that it was only possible to conduct a few meta-analyses, which, in some instances, included data from only two studies. Caution is therefore warranted in the interpretation of the results from these analyses, with further empirical studies needed. Furthermore, we were unable to explore the potential influence of moderating variables, nor possible sources of heterogeneity between studies [32], and therefore, the results of these analyses should be considered tentative until further research is conducted. Similar limitations are observed in the CPM literature as in the wider QST literature [86], and we offer a number of recommendations for future research in young people. First, details should be provided on the training researchers have received in administering CPM procedures, much in the same way that reports of therapeutic interventions typically provide details on the training and experience of the providing therapists. In the present review, only seven studies clearly stated that the assessors had training or were experienced with the CPM procedures and instruments used. The heterogeneity observed in several of the analyses conducted may be at least partly due to differences in assessor training or experience. Second, where possible, we encourage authors to present CPM results separately for different age groups, ethnic groups, and sexes, as these variables are known to shape clinical pain responses (e.g., [87,88,89]). In adults, the CPM effect is shown to progressively decline with age [26,90,91], while the effects of ethnicity and sex are less consistent, with further investigation needed [91]. Few studies in the present review explored the potential importance of these variables in CPM responses in CYP, with firm conclusions unable to be drawn from the limited data available. Finally, variations in precise CPM methodology were shown across the studies included in the present review, and sub-analyses revealed significantly weaker CPM responses in CYP with chronic pain when heat pain test stimuli, but not pressure pain test stimuli, were used. We were unable to further explore the potential importance of implementing different forms of tests and conditioning stimuli, however. Notably, the adult CPM literature also shows considerable variation in testing stimuli and parameters [91]. Development of standardized CPM methodology across the developmental trajectory is warranted [92].

## 5. Conclusions

CPM appears to be impaired in paediatric populations with chronic pain in comparison to healthy controls. Variation in the results is noted, however, and across the broader literature, both pain characteristics and CPM testing parameters have been shown to impact the results obtained. Improvements in reporting are needed, including the training and experience of CPM assessors and psychometric properties of the outcome measures used, along with testing the psychometric properties of the CPM paradigm. Further research is needed that explores the potential importance of individual difference variables, including age, sex, and ethnicity, on CPM effects, which to date have received limited investigation in studies recruiting participants from younger populations.

## Data Availability

The raw data supporting the conclusions of this article will be made available by the corresponding author upon reasonable request.

## Supplementary Materials

The following supporting information can be downloaded at: https://www.mdpi.com/article/10.3390/children11111367/s1; Table S1: Details of each study included in the systematic review and meta-analysis of conditioned pain modulation in children; Table S2: Outcome measures and their psychometric properties for studies included in a systematic review and meta-analysis of conditioned pain modulation in children and adolescents.

## Appendix A. Conditioned Pain Modulation Parameters and Overall Results for Each Study Included in the Systematic Review and Meta-Analysis of Conditioned Pain Modulation in Children and Adolescents

**Study**	**Sample Details**	**CPM Test Stimulus**	**Test and Conditioning Sites**	**CPM Testing Procedure**	**Main CPM Findings**
Arribas-Romano, Fernández-Carnero et al. (2024) [68]	30 pain-free adolescents	Test: Pressure pain threshold via handheld pressure algometerConditioning: Pressure pain via sphygmomanometer	Test site: Right thumb nail bedConditioning site: Left arm	Pressure pain test stimulus administered before, during, and one minute after conditioning pressure stimulus	Reported pain intensity to the test stimulus was lower during the presence of the conditioning stimulus than at baseline.
Brandão, Talih et al. (2024) [51]	Participants from the Generation XXI birth cohort.1496 adolescents. At age thirteen, 883 reported having any pain, of which 401 reported having musculoskeletal pain as their principal pain site.143 adolescents reported current musculoskeletalpain with more than 3 months’ duration. 50 reported current musculoskeletal painin addition to musculoskeletal painin one or more of the previous evaluation waves at ages 7and 10.	Test: Pressure pain threshold and tolerance via automated cuff algometerConditioning: Pressure pain via automated cuff algometer	Test site: Right legConditioning site: Left leg	Pressure pain test stimulus administered before and during conditioning pressure stimulus	For adolescents with musculoskeletal pain at 13 plus a history of pain, the CPM effect was slightly increased for pain detection threshold and pain tolerance compared to other participants, although this effect was not statistically significant. Impaired CPM was not detected among adolescents with musculoskeletal pain. The same pattern of results was found for adolescents with musculoskeletal pain at seven and ten with a history of pain.
Chrétien, Lavoie et al. (2018) [43]	16 teenage girls with chronic pain and 25 healthy adolescent girls	Test: Heat pain intensity rating via thermodeConditioning: Cold pressor	Test site: Volar part of the left forearmConditioning site: Right forearm	Heat pain administered before and immediately after cold pressor	Pain intensity produced by heat pain stimulationssignificantly decreased following cold pressor pain in healthy girls, but not in girls with chronic pain. CPM magnitude was significantly greater in healthy girls compared to those with chronic pain.
Evans, Seidman et al. (2013) [58]	133 healthy children and adolescents	Test: Pressure pain intensity rating via hydraulic piston connected to a computer activated pumpConditioning: Cold pressor	Test site: Thumbnail of left handConditioning site: Right hand	Pressure pain administered before, during, and after cold pressor	For boys, but not for girls, higher material anxiety was significantly associated with lower CPM (less pain inhibition). CPM magnitude was similar for boys and girls.
Ferland, Teles et al. (2019) [56]	105 paediatric patients with chronic back pain	Test: Heat pain intensity via thermodeConditioning: Cold pressor	Test site: Right forearmConditioning site: Left forearm	Heat pain administered before and during cold pressor	High blood metanephrine was a significant predictor of poorer CPM efficiency, explaining 53% of CPM variation in males and 7% of CPM variation in females.
Goffaux, Lafrenaye et al. (2008) [61]	26 children term-born or born pre-term exposed to numerous painful interventions or few painful interventions	Test: Heat pain intensity via thermodeConditioning: Cold pressor	Test site: Left calf and left forearmConditioning site: Right hand	Heat pain administered before and during cold pressor	Cold pressor pain was found to significantly reduce test heat pain intensity for full-term and low-pain pre-term groups with strong inhibitory responses. The high-pain pre-term group demonstrated a complete absence of CPM effect.
Harper & Hollins (2017) [64]	37 healthy undergraduate students	Test: Heat pain intensity via thermal grillConditioning: Cold pressor	Test site: Right volar forearmConditioning site: Left hand	Heat pain administered during cold pressor run and control run (neutral temperature water), the order of which was counterbalanced	CPM produced significant and comparable reductions in pain in noxious heat and thermal grill conditions.
Hoehn (2022) [67]	54 healthy children	Test: Pressure pain threshold via algometerConditioning: Cold pressor	Test site: Right thumbnailConditioning site: Left hand	Pressure pain administered before, during, and after cold pressor	A significant CPM effect was observed, with no significant main effect of age (6–12 years)
Holden, Rathleff et al. (2020) [48]	151 adolescents with patellofemoral pain and 50 healthy controls	Test: Pressure pain threshold via computerised cuff pressure algometerConditioning: Pressure pain via computerised cuff pressure algometer	Test site: LegConditioning site: Leg(For those with patellofemoral pain, the test limb was the knee with pain or the most painful knee in the case of bilateralpain. The test limb was randomly selected for controls)	Test pressure pain stimulus administered before and during the conditioning stimulus	The healthy control group had a significant increase in pain detection threshold during the painful conditioning stimulus compared to without conditioning. No significant difference was found for the patellofemoral pain group, indicating no efficient CPM response.
Holden, Straszek et al. (2018) [49]	36 young females with patellofemoral pain, 22 recovered from patellofemoral pain, and 29 healthy controls	Test: Pressure pain threshold and tolerance via computerised cuff pressure algometerConditioning: Pressure pain via computerised cuff pressure algometer	Test site: LegConditioning site: Leg(For those with or recovered from patellofemoral pain, the test limb was the knee with pain or the most painful knee in the case of bilateralpain. The test limb was randomly selected for controls)	Test pressure pain stimulus administered before and during the conditioning stimulus	Considering pressure tolerance threshold, females with patellofemoral pain had significantly impaired CPM compared to those recovered from patellofemoral pain, but no differences in CPM compared to healthy controls. No differences were found in CPM when pressure pain threshold was considered.
Jørgensen, Werner et al. (2024) [50]	25 children and adolescents with cerebral palsy (9 with chronic pain and 16 without chronic pain) and 26 typically developed children (TDC) and adolescents (14 with chronic pain and 12 without chronic pain)	Test: Pressure pain threshold via handheld pressure algometerConditioning: Cold pressor	Test sites: Hand and footConditioning sites: Hand and foot(Test and conditioning sites were (i) both hands, and (ii) both feet)For all participants except TDC without chronic pain, the primary test site was the most affected limb.The non-dominant hand or foot site was randomlychosen in TDCwithout chronic pain.	Pressure pain stimulus administered before and during the cold pressor	Across all participants, pressure pain threshold consistently increased by a median of approximately 30 kPa from unconditioned to conditioned test stimuli. No other effects were found.
Leone, Caterina et al. (2021) [63]	30 adolescents with non-suicidal self-injury (NSSI) and 20 healthy controls	Test: Heat pain intensity via thermodeConditioning: Heat pain via thermode	Test site: Non-dominant volar forearmConditioning site: Dominant forearm	Test heat pain administered before and during conditioning heat pain	Adolescents with NSSI showed significantly deficient CPM compared to healthy adolescents.
Lucas, Talih et al. (2024) [66]	1727 children and adolescents classified based on a bullying scale as aggressor only, victim only, both victim and aggressor, or not involved	Test: Pressure pain threshold and tolerance threshold via computerised cuff pressure algometerConditioning; Pressure pain via computerised cuff pressure algometer	Test site: Lower right legConditioning site: Lower left leg	Test pressure pain stimulus administered before and during the conditioning stimulus	Children classified as aggressors only had higher CPM for pressure detection thresholds compared to those not involved in bullying.
Morris, Bruehl et al. (2021) [52]	63 adolescents with functional abdominal pain (FAP) receivinginternet-delivered cognitive behaviour therapy (*n* = 90) or pain education (*n* = 93)	Test: Heat pain intensity via thermodeConditioning: Hot water immersion	Test site: Ventral forearm of non-dominant handConditioning site: Dominant hand	Test heat pain administered before and during hot water immersion	Beyond any effects of the interventions, pain-related interference declined significantly over time for children with stronger baseline CPM.
Morris, Walker et al. (2015) [59]	78 healthy children	Test: Heat pain intensity via thermodeConditioning: Hot water immersion	Test site: Ventral forearm of non-dominant handConditioning site: Dominant hand	Test heat pain administered before and during hot water immersion	Significantly stronger CPM effects were found in African-American children than Non-Hispanic White children
Morris, Walker et al. (2016) [44]	63 youth with functional abdominal pain (FAP) and 77 healthy controls	Test: Heat pain intensity via thermodeHot water immersion	Test site: Ventral forearm of non-dominant handConditioning site: Dominant hand	Test heat pain administered before and during hot water immersion	Children with FAP showed significantly weaker CPM responses than healthy controls
Nahman-Averbuch, Leon et al. (2019) [30]	19 adolescentswith migraine, 20 healthy adolescents with a family history of migraine, and 29 healthy adolescents without a family history of migraine	Test: Heat pain intensity via thermode, pressure pain threshold via algometerConditioning: Cold pressor	Test site: Both stimuli at the lower dominant legConditioning site: Non-dominant foot	Heat/pressure pain administered before and during cold pressor	No significant difference in CPM magnitude was found between the three groups.
Nahman-Averbuch, Schneider et al. (2021) [57]	20 adolescentswith migraine	Test: Pressure pain threshold via algometerConditioning: Cold pressor	Test site: Lower dominant leg and trapeziusConditioning site: Non-dominant foot	Pressure pain administered before and during cold pressor	A significant CPM response was seen in children before CBT, although the effect was variable.
Nahman-Averbuch, Thomas, et al. (2021) [40]—Secondary analysis of above 2021 [57] study, recruited additional healthy controls	19 adolescentswith migraine and 20 healthy adolescents	Test: Pressure pain threshold via algometerConditioning: Cold pressor	Test site: Lower dominant leg and trapeziusConditioning site: Non-dominant foot	Pressure pain administered before and during cold pressor	No significant difference in CPM magnitude was found between adolescents with migraine and healthy controls.
Ocay, Larche et al. (2022) [41]	302 adolescents with chronic musculoskeletal pain and 80 healthy controls	Test: Heat pain intensity via thermodeConditioning: Cold pressor	Test site: Right volar forearmConditioning site: Left forearm	Heat pain administered before and after cold pressor	CPM efficiency was significantly weaker in children with musculoskeletal pain than in healthy controls. Adolescents with chronic musculoskeletal pain were heterogenous, with distinct subgroups identified, including patients who did and did not display optimal CPM efficiency
Ocay, Ye et al. (2022) [47]	639 children and adolescents with chronic pain and 60 healthy controls (regrouped data from multiple former studies)	Test: Heat pain intensity via thermodeConditioning: Cold pressor	Test site: Right volar forearmConditioning site: Left forearm	Heat pain administered before and after cold pressor	Mean CPM efficiency did not significantly differ between those with chronic pain and healthy controls. Cluster analysis was performed, which revealed heterogeneity amongst patients in their responses to CPM
Ocay, Loewen et al. (2022) [53]	198 adolescents with chronic back pain	Test: Heat pain intensity via thermodeConditioning: Cold pressor	Test site: Right forearmConditioning site: Left arm	Heat pain administered before and after cold pressor	CPM efficiency was optimal in 51.5% of adolescents, suboptimal in 22.7% of adolescents, and inefficient in 25.8% of adolescents.
Pas, Rheel et al. (2019) [45]	39 children and youth with functional abdominal pain disorder and 36 healthy controls	Test: Pressure pain threshold via algometerConditioning: Cold pressor	Test site: Dominant trapezial regionConditioning site: Non-dominant hand	Pressure pain administered before and during cold pressor	Children with functional abdominal pain showed significantly lower CPM responses than healthy children
Ray & O’Connor (2023) [62]	54 healthy young women randomised to receive yoga/no yoga and slow-breathing/normal breathing (*n* = 11–15 per group)	Test: Heat pain intensity via thermodeConditioning: Cold pressor	Test site: Right forearmConditioning site: Left hand	Test heat pain administered before and during cold pressor	No significant effects of either yoga or slow breathing on CPM efficiency were found.
Schubert-Hjalmarsson, Fasth et al. (2023) [42]	10 children with hypermobility spectrum disorderor hypermobile Ehlers–Danlos syndrome and 9 healthy controls	Test: Pressure pain threshold via handheld pressure algometerConditioning: Cold pressor	Test site: Trapezius (side not stated)Conditioning site: Non-dominant hand	Pressure pain stimulus administered before and during (30s and 50s) the cold pressor	Only descriptive statistics are reported. A similar CPM outcome was found for patient and control groups.
Stolzman & Bement (2016) [60]	32 normal-weightadolescents and 24 overweight/obese adolescents	Test: Pressure pain threshold via algometerConditioning: Cold pressor	Test site: Left 4th digit nailbed and left middle deltoid muscleConditioning site: Right foot	Pressure pain administered during cold pressor and during cool water (control condition)	CPM responses were similar across weight status between boys and girls.
Teles, Ocay et al. (2019) [54]	Ninety-four adolescents diagnosed with idiopathic scoliosis and chronic back pain	Test: Heat pain intensity via thermodeConditioning: Cold pressor	Test site: Left volar forearm (control area), most painful location on the back (affected area)Conditioning site: Hand and arm	Heat pain administered before and after the cold pressor	Efficient pain inhibitory response was shown by 51.1% of children, while 21.3% had sub-optimal CPM and 27.7% had inefficient CPM
Tham, Li et al. (2024) [29]	77 adolescents with functional abdominal pain	Test: Heat pain threshold via thermodeConditioning: Cold pressor	Test site: Volar surface of the dominant forearmConditioning site: Non-dominant hand and wrist	Heat pain administered during the cold pressor conditioning stimulus	Higher CPM was significantly correlated with lower abdominal pain intensity. When adjusted for age and sex, this was no longer significant, although it was marginally associated with increased pain interference.
Tsao, Seidman et al. (2013) [31]	133 healthy children	Test: Pressure pain intensity via hydraulic piston connected to a computer-activated pumpConditioning: Cold pressor	Test site: Thumbnail of left handConditioning site: Right hand	Pressure pain administered before, during, and after the cold pressor	A significant CPM effect was revealed for the whole sample, although younger children (8–11 years) showed significantly less CPM than adolescents (12–17 years). No significant difference in CPM was found between boys and girls.
Uzawa, Takeuch et al. (2024) [65]	32 healthy young people (14 female, 18 male)	Test: Pressure pain threshold via handheld pressure algometerConditioning: Cold pressor	Test site: Left trapeziusConditioning site: Right hand	Pressure pain stimulus administered before and after the cold pressor	No significant differences in CPM indices were found between males and females. Sympathetic and parasympathetic nervous system activitieswere significantly associated with CPM effects across all participants. Descending pain modulations in women might be more associated with autonomic activities than those in men.
Verriotis et al. (2021) [55]	52 adolescents with neuropathic pain and 14 adolescents with complex region pain syndrome	Test: Pressure pain threshold via algometerConditioning: Cold pressor	Test site: Head of fibulaConditioning site: Contralateral handTypically, pressure test pain was applied on the right knee with the lefthand for conditioning, but if patients reported pain in the left hand orright knee, this was reversed.	Pressure pain administered before, during, and after the cold pressor	CPM was found to be inhibitory in 54% of children and facilitatory in 14% of children.
Williams, Heitkemper et al. (2013) [46]	22 girls with irritable bowel syndrome (IBS) and 21 healthy girls	Test: Heat pain threshold via thermodeConditioning: Cold pressor	Test site: Volar surface of right forearmConditioning site: Left hand	Heat pain administered before and during cold pressor	Girls with IBS did not show significant endogenous pain inhibition. A significant difference between the two groups was observed for endogenous pain inhibition, which was deficient in girls with IBS compared to healthy controls.

## Appendix B. Between-Groups Forest Plots for Individual Analyses Conditioned Pain Modulation Response in Children and Young People with to Healthy Controls

Analysis 1. CPM responses in CYP with chronic pain compared to healthy controls using heat test-stimulus data from Nahman-Averbuch and colleagues (2019).

Analysis 2. CPM responses in CYP with chronic pain compared to healthy controls using pressure pain test-stimulus data from Nahman-Averbuch and colleagues (2019).

Analysis 3. CPM responses in CYP with chronic pain compared to healthy controls using data from heat pain test stimuli only.

Analysis 4. CPM responses in CYP with chronic pain compared to healthy controls using data from pressure pain test stimuli only.

Analysis 5. CPM responses in CYP with abdominal pain compared to healthy controls.

Analysis 6. CPM responses in CYP with migraine compared to healthy controls using pressure pain test-stimulus data from Nahman-Averbuch and colleagues (2019).

Analysis 7. CPM responses in CYP with migraine compared to healthy controls using heat test-stimulus data from Nahman-Averbuch and colleagues (2019).
