# Supplementary material for: A Systematic Review and Meta-Analysis of Conditioned Pain Modulation in Children and Young People with Chronic Pain
Source: Children (Basel). 2024 Nov 11;11(11):1367. doi: 10.3390/children11111367 (PMC11592744; doi:10.3390/children11111367)
Supplement: Supplementary file 1 [file children-11-01367-s001.zip › Table S1 - CPM Data extraction 05-09-24.docx]

**Table S1**

*Details of each study included in the systematic review and meta-analysis of conditioned pain modulation in children*

| **Study Country** | **Participant demographics and medical characteristics** | **Conditioned pain modulation protocol details** | **Pain and additional outcome measures** | **Conditioned pain modulation results and outcome results (mean and SD unless stated otherwise)** |
| --- | --- | --- | --- | --- |
| **Arribas-Romano, Fernández-Carnero et al. (2023)** Spain | **30 pain-free students**  Age: 21 (20 to 24)  Sex: 24 female, 6 male  Height (cm): 167 (162, 171)  Weight (kg): 58.5 (55, 66)  BMI: 21.4 (20.6, 23.7)  Students were pain-free, without having experienced any episodes of chronic pain (pain for more than 3 months) in the last 6 months.  [Thirty adolescents with neck pain were also recruited, although their age range does not fit this review’s inclusion criteria and therefore their results are not reported here | **Test stimulus: Pressure pain** Pressure pain threshold (PPT) assessed via handheld algometer with a 1cm^2^ rubber head on the nail bed of the right thumb. Pressure applied at a rate of 1kg/s until the participant reported the onset of pain.  **Conditioning stimulus: Pressure pain** Applied via sphygmomanometer placed on the left arm, with its lower edge 3cm proximal to the ulnar fossa. The cuff was inflated to 260mmHg and held until the participant perceived pain of 6–7/10 on a Numeric Pain Rating Scale (NPRS).  **Procedure** Test pressure pain threshold was assessed before, during, and one-minute after the conditioning stimulus. | **Anxiety**  Spanish adapted version of the State Anxiety Inventory.  **Depression**  Spanish adapted version of the Beck  Depression Inventory II  **Pain Catastrophising**  Spanish adapted version of the Pain  Catastrophising Scale.  **Kinesiophobia**  Spanish version of the Tampa Scale for Kinesiophobia, assessing the degree of fear of movement and (re)injury.  **Numeric Pain Rating Scale**  0 (no pain) to 10 (the worst pain ever felt) scale. | **Pain-free participants (*n* = 30)** PPT thumb (kg/cm^2^): 5.56 (1.85) CPM (difference scores, kg/cm^2^): 2.10 (1.64)  State Anxiety Inventory: 16.3 (11.7) Beck Depression Inventory: 3(0–8)* Tampa Scale for Kinesiophobia: 18.4 (5.7) Pain Catastrophising Scale: 7 (1–15)*  * Medians and interquartile range  Reported pain intensity to the test stimulus was lower during the presence of the conditioning stimulus than at baseline. |
| **Brandão, Talih et al. (2023)**  Portugal | **1496 participants with a pain history at age 13: 723 female, 773 male.**  Musculoskeletal (MSK) principal pain sites *n* (%): 401 (26.8). Neck and shoulders: 12 (3.0), upper limbs: 46 (11.5), back: 81 (20.2), hips: 7 (1.7), lower limbs: 257 (64.1).  MSK pain case definitions, *n* (%): Cross-sectional (current chronic MSK pain > 3 months duration): No: 1353 (90.4), Yes: 143 (9.6). Longitudinal (current MSK pain with MSK pain history reported at ages 7 and/or 10): No: 1446 (96.7), Yes: 50 (3.3)  Causes of principal pain, *n* (%): 250 (16.7). Any physical: 109 (43.6), Any psychosocial: 33 (13.2), related to activities of daily living: 188 (75.2), Indefinite or unclassified: 12 (4.8) | **Test stimulus: Pressure pain** Assessed via cuff pressure algometry on the right leg for analysis. Pressure pain detection and pain tolerance thresholds were both assessed. The cuff was inflated at a rate of 1kPa/s. Participants indicated once the pressure became painful using an electronic slider on a VAS, and to continued rating the pain until the sensation was no longer tolerable. Pain detection threshold was defined as the pressure value when the VAS moved 1cm. Pain tolerance threshold was defined as the pressure when participants moved the VAS to 10cm.  **Conditioning stimulus: Pressure pain** Assessed via cuff pressure algometry on the left leg, set to 70% of the pain tolerance threshold with a 100 kPa/s inflation and deflation rate.  **Procedure** Test pressure pain threshold was assessed before and during the conditioning stimulus. | **Pain Sensitivity**  Pressure pain detection and pain tolerance thresholds as described  **Temporal summation of pain (TSP)**  Assessed via cuff pressure algometry. Ten sequential pressure stimuli were applied on the right leg with an intensity equal to the pain tolerance threshold, with a one-second duration and one-second interval. Participants rated their perceived pain by adjusting the VAS, with scores for each stimulus extracted. Stimuli 1–4 (VAS-I) and 8–10 (VAS-II) were averaged. The TSP effect was computed as the difference between VAS-I and VAS-II (i.e. VAS-II minus VAS-I). | **QST Parameters at age 13, mean (SD)** Pain Sensitivity   \| Pain Sensitivity (*n*) \| Girls (723) \| Boys (773) \| Total (1496) \| \| --- \| --- \| --- \| --- \| \| Pain Detection Thresholds (PDT) \| 20.9 (11.7) \| 20.6 (12.2) \| 20.7 (12.0) \| \| Pain Tolerance Thresholds (PTT) \| 52.5 (20.3) \| 51.8 (19.9) \| 52.1 (20.1) \| \| Difference between PTT and PDT \| 31.6 (16.4) \| 31.2 (16.3) \| 31.4 (16.4) \|   Temporal Summation of Pain   \| Temporal Summation of Pain (*n*) \| Girls (580) \| Boys (634) \| Total (1214) \| \| --- \| --- \| --- \| --- \| \| VAS-I \| 3.64 (2.23) \| 3.96 (2.28) \| 3.80 (2.26) \| \| VAS-II \| 4.74 (2.49) \| 4.95 (2.38) \| 4.85 (2.43) \| \| Difference between VAS-II and VAS-I \| 1.10 (1.11) \| 0.98 (1.14) \| 1.04 (1.13) \|   Conditioned Pain Modulation   \| Conditioned Pain Modulation (*n*) \| Girls (421) \| Boys (408) \| Total (829) \| \| --- \| --- \| --- \| --- \| \| Conditioned pain detection threshold \| 31.6 (18.3) \| 32.2 (19.1) \| 31.9 (18.7) \| \| Conditioned pain tolerance threshold \| 59.6 (22.1) \| 61.1 (21.6) \| 60.3 (21.9) \| \| Difference between conditioned and baseline PDT \| 10.6 (14.6) \| 11.7 (14.7) \| 11.1 (14.7) \| \| Difference between conditioned and baseline PTT \| 8.4 (10.8) \| 10.3 (11.1) \| 9.3 (11.0) \|   **Comparison of QST medians (25^th^-75^th^ percentiles) between participants at age 13 with two musculoskeletal pain case definitions and the remaining adolescents (Mann-Whitney test)**  Pain Sensitivity   \| Pain Sensitivity (*n*) \| Current chronic musculoskeletal pain (143) \| Remaining adolescents (1353) \| *p* value \| \| --- \| --- \| --- \| --- \| \| Pain detection thresholds (PDT; in kPa) \| 20.0 (12.9–27.5) \| 18.0 (12.2–25.9) \| 0.244 \| \| Pain tolerance thresholds (PTT; in kPa) \| 49.4 (35.7–67.1) \| 47.8 (37.3–63.5) \| 0.723 \|   Conditioned Pain Modulation   \| Conditioned Pain Modulation (*n*) \| Current chronic musculoskeletal pain (56) \| Remaining adolescents (558) \| *p* value \| \| --- \| --- \| --- \| --- \| \| Difference between conditioned and baseline PDT \| 7.65 (0.10-17.0) \| 7.45 (0.78-16.5) \| 0.980 \| \| Difference between conditioned baseline and PTT \| 8.04 (1.18–20.1) \| 8.24 (2.25–15.0) \| 0.641 \|   Temporal Summation of Pain   \| Temporal Summation of Pain (*n*) \| Current chronic musculoskeletal pain (114) \| Remaining adolescents (1100) \| *p* value \| \| --- \| --- \| --- \| --- \| \| VAS-I (in cm) \| 3.38 (1.97–5.56) \| 3.39 (2.00–5.47) \| 0.781 \| \| VAS-II (in cm) \| 4.74 (2.84–6.48) \| 4.67 (2.92–6.62) \| 0.834 \| \| Difference between VAS-II and VAS-I \| 0.91 (0.23–1.67) \| 0.86 (0.35–1.66) \| 0.514 \|   Pain Sensitivity   \| Pain Sensitivity (*n*) \| Current chronic musculoskeletal pain with a history of musculoskeletal pain at ages 7 and/or 10 (50) \| Remaining adolescents (1446) \| *p* value \| \| --- \| --- \| --- \| --- \| \| Pain detection threshold (PDT; in kPa) \| 14.5 (10.2–25.7) \| 18.2 (12.2–26.3) \| 0.072 \| \| Pain tolerance threshold (PTT; in kPa) \| 40.2 (31.2–60.7) \| 49.0 (37.3–63.9) \| 0.020 \|   Conditioned Pain Modulation   \| Conditioned Pain Modulation (*n*) \| Current chronic musculoskeletal pain with a history of musculoskeletal pain at ages 7 and/or 10 (20) \| Remaining adolescents (594) \| *p* value \| \| --- \| --- \| --- \| --- \| \| Difference between conditioned and baseline PDT \| 7.65 (−1.08–15.6) \| 7.45 (0.78–16.5) \| 0.595 \| \| Difference between conditioned baseline and PTT \| 5.49 (−0.20–15.5) \| 8.24 (2.25–15.3) \| 0.471 \|   Temporal Summation of Pain   \| Temporal Summation of Pain (*n*) \| Current chronic musculoskeletal pain with a history of musculoskeletal pain at ages 7 and/or 10 (40) \| Remaining adolescents (1174) \| *p* value \| \| --- \| --- \| --- \| --- \| \| VAS-I (in cm) \| 3.16 (2.00–5.63) \| 3.40 (2.01–5.46) \| 0.917 \| \| VAS-II (in cm) \| 4.73 (2.87–6.39) \| 4.68 (2.91–6.62) \| 0.831 \| \| Difference between VAS-II and VAS-I \| 1.06 (0.48–1.49) \| 0.85 (0.32–1.67) \| 0.557 \|   **Conditioned Pain Modulation** For adolescents with musculoskeletal pain at 13 plus a history of pain, the CPM effect was slightly increased for pain detection threshold and pain tolerance compared to other participants, although this effect was not statistically significant. Impaired CPM was not detected among adolescents with musculoskeletal pain. The same pattern of results was found for adolescents with musculoskeletal pain at seven and ten with a history of pain. |
| **Chrétien, Lavoie et al. (2018)** Canada | **Chronic pain group**  16 teenage girls with chronic/recurrent pain Age: 15.7 (SE 0.2) BMI: 21.5 (SE 0.9) Sum of skin thickness (mm): 43.0 (SE 1.1) Physical activity (hours per week): 10.4 (SE 1.7) VO_2_ max (mL/min): 1718.6 (SE 73.5)  **Healthy Adolescents group**  25 healthy adolescent females Age: 15.8 (SE 0.2) BMI: 21.5 (SE 0.5) Sum of skin thickness (mm): 39.1 (SE 0.9) Physical activity (hours per week): 15.2 (SE 1.7) VO_2_ max (mL/min): 1978.9 (SE 48.1) | **Test Stimulus: Heat Pain**  A 30 × 30 mm thermode was used to induce thermal pain stimulations on the volar part of the left forearm. The initial temperature was 37 °C, which increased at a fixed rate of 0.3 °C per second. Participants reported when sensation changed from heat sensation to minimal pain (heat pain threshold) and when the pain could no longer be sustained (heat pain tolerance). Two trials were performed, and mean ratings calculated.  Following this, the thermode was placed on the volar part of the left forearm for 5 seconds. A constant temperature (heat pain stimulation) was administered, which was computed individually for each participant, and which corresponded to mild (30%) pain intensity using the formula: [0.3 (heat pain tolerance - heat pain threshold)] + heat pain threshold. Participants evaluated the pain intensity immediately after, with the procedure was performed before and after the cold pressor task.  **Conditioning Stimulus: Cold Pressor Test (CPT)**  Participants were instructed to immerse their right forearm in a bath of circulating 10 °C cold water for 2 minutes.  **Procedure**  Heat pain threshold and tolerance values were first computed for the participant, after which heat pain stimulations at mild intensity (30%) were completed. Heat pain stimulations were performed before and immediately after the cold pressor task to evaluate CPM. | **Anxiety**  State-Trait Anxiety Inventory (STAI) to assess state and trait anxiety.  **Depression**  Children’s Depression Inventory  **Pain Catastrophising**  Pain Catastrophising Scale  **Sleep Quality**  Pittsburgh Sleep Quality Inventory  Measures are simply listed in this article, and specific details such as measure versions are not provided.  **Graded exercise test**  A maximal oxygen capacity (VO2max) test performed via cycle ergometer. Participants initially completed a three-minute warm-up of 60 revolutions per minute (rpm) without resistance. Resistance was then gradually added with a ramp protocol, individualized to reach the maximal theoretical aerobic power between 8 and 12 min (increments 15–30 W/min). Participants were encouraged to keep a constant rate of  approximately 60 rpm.  **Cold pain stimulation**  A 2 × 2 cm ice cube placed on four different sites of the left arm (volar portion) for 10 seconds. Pain intensity was evaluated with a verbal numeric rating scale at the end of the procedure. | **Means and standard errors**   \|  \| **Healthy (*n* = 25)** \| **Chronic pain (*n* = 16)** \| \| --- \| --- \| --- \| \| State anxiety (STAI) \| 32.2 (1.3) \| 35.4 (1.9) \| \| Trait anxiety (STAI) \| 35.8 (1.9) \| 39.8 (1.4) \| \| Depression symptoms (CDI) \| 8.8 (0.9) \| 14.6 (1.7) \| \| Pain Catastrophizing Scale \| 16.5 (1.8) \| 19.9 (2.5) \| \| Sleep quality (PSQI) \| 4.3 (0.5) \| 7.1 (0.8) \|   **Conditioned pain modulation**  Pain rated on a 0 – 10 scale: 0 = ‘none’, 10 = ‘most intense pain tolerable.  Pain intensity evaluations for heat pain stimulations before CPT did not significantly differ between healthy girls and girls with and chronic pain (3.0 ± 0.2 vs. 3.2 ± 0.3 respectively; *t* = 0.5, *p* = .60).  Healthy adolescent girls showed a significant reduction in pain intensity induced by heat pain stimulations after the cold pressor test (3.0 ± 0.2 vs. 2.2 ± 0.2 before and after CPT respectively; *t* = 4.0, *p* = .0006).  Girls with chronic pain showed no significant reductions in pain intensity induced by heat pain stimulations after the cold pressor test (3.2 ± 0.3 vs. 3.0 ± 0.2 before and after CPT respectively; *t* = 0.8, *p* = .40).  The effectiveness of CPM was significantly greater in healthy girls compared to girls with chronic pain (26.7 ± 8.1 vs. −1.9 ± 9.6 respectively; *t* = 2.2, *p* = .03).  **Exercise induced analgesia** In healthy adolescents, pain intensity induced by the ice cube significantly decreased after physical exercise (3.5 ± 0.5 before vs. 2.7 ± 0.4 after exercise; *p* = .001). A significant reduction was not found in the chronic pain group (3.1 ± 0.7 before vs. 3.1 ± 0.7 after exercise; *p* = .9). Ice cube pain intensity before the graded exercise test were comparable between groups (3.5 ± 0.5 healthy vs. 3.1 ± 0.7 pain; t = 0.6, *p* = .6).  The effectiveness of exercise induced analgesia was greater in healthy participants than adolescents with chronic pain (23.8 ± 6.1 compared to. −12.0 ± 11.2 respectively; *p* = .004).  All data are presented as means and standard error of the mean. |
| **Evans, Seidman et al. (2013)**  USA | **133 healthy children and adolescents**  Aged 8-17 years (13.0 ± 2.9).  70 female, 63 male  41 Hispanic/Latino, 92 Non-Hispanic/non-Latino.  56 White  33 African American  2 Asian  1 American Indian/Alaska native  38 Multiracial  3 unspecified  133 mothers of child participants  Age 43.2 ± 7.3  37 Hispanic/Latino, 96 Non-Hispanic/Non-Latino  67 White  34 African American  4 Asian  1 American Indian/Alaska native  2 Native Hawaiian  21 Multiracial  4 Unspecified | **Test Stimulus: Pressure Pain**  Discrete 5-second pressure stimuli applied to the thumbnail of the left hand with a 1x1cm rubber probe controlled by computer activated hydraulic piston. Stimuli were presented ascending from .066 kg/cm^2^, increasing in .132 kg/cm^2^ intervals until the participant reported moderate pain (a rating of 6 on the 0–10 NRS) or to a maximum of 1.12 kg/cm^2^. Then, stimuli were delivered at 15-second intervals in random order, using the multiple random staircase pressure pain sensitivity method. The final 4 pressure steps on the high staircase were averaged and rounded up to 1 of 24 pressure levels (.05–1.20 kg/cm^2^) to be used as the test stimulus in the CPM task.  **Conditioning Stimulus: Cold pressor test**  Participants placed their right hand in a cold pressor unit, submerged up to approximately 2-inches above the wrist with water circulating at a temperature of 5°C.  **Procedure**   1. TS administered without the cold-pressor conditioning stimulus to obtain a baseline TS pain rating. 2. At 17s total, participants were administered the cold pressor task. 3. At 39s total, while the right hand was immersed in the cold water, the TS was administered a second time. 4. At 47s total, conditioning stimulus stopped. 5. At 65s total, the TS was administered a third time (TS3). 6. At 117s total, TS was administered a fourth time   Throughout, TS pain ratings using the 0–10 NRS were made immediately following each 5-second TS administration.  CPM magnitude is calculated as the difference score between TS1 and TS3 | **Pain: Numerical Rating Scale**  0-10 scale. The higher the score the more pain felt in relation to the cold-pressor task.  **Anxiety: Numerical Rating Scale**  0-10 scale. The higher the score the more anxiety experienced. Anticipatory anxiety was assessed prior to CPM task  **Pain-related Fear: Numerical Number Scale**  0-10 scale. Assessed immediately after the end of the CPM task. The higher the score the more pain-related fear experienced.  **Maternal fear of pain & pain related anxiety: Pain Anxiety Symptoms Scale-20-Item Version (PASS-20)**  Four 5-item subscales measuring cognitive, escape/avoidance, fear and physiological anxiety. Higher scores indicate increased fear of pain and pain related anxiety.  **Maternal general psychological distress: Brief Symptom Inventory-18-Item Version (BSI-18), Global Severity Index (GSI)**  3 subscales measuring somatization, depression and anxiety. Higher scores indicated greater overall psychological distress. | **Data are provided for boys and girls separately.**  **Boys:**   \|  \| M \| SD \| \| --- \| --- \| --- \| \| CPM Anxiety \| 4.05 \| 3.0 \| \| CPM Magnitude (Pain at TS3-Pain at TS1) \| -1.46 \| 2.0 \| \| CPM Fear \| 4.31 \| 3.3 \| \| Mothers’ PASS-20 escape/avoidance \| 9.63 \| 4.8 \| \| Mothers’ PASS-20 fear \| 3.86 \| 4.5 \| \| Mothers’ PASS-20 cognitive \| 8.21 \| 5.8 \| \| Mothers’ PASS-20 physiological anxiety \| 3.48 \| 4.4 \| \| Mothers’ PASS-20 total score \| 25.17 \| 16.5 \| \| Mothers’ BSI-18 GSI \| 6.95 \| 6.7 \|   **Girls:**   \|  \| M \| SD \| \| --- \| --- \| --- \| \| CPM Anxiety \| 4.11 \| 2.9 \| \| CPM Magnitude (Pain at TS3-Pain at TS1) \| -1.51 \| 2.3 \| \| CPM Fear \| 4.20 \| 2.8 \| \| Mothers’ PASS-20 escape/avoidance \| 9.37 \| 6.2 \| \| Mothers’ PASS-20 fear \| 3.87 \| 4.7 \| \| Mothers’ PASS-20 cognitive \| 7.37 \| 5.8 \| \| Mothers’ PASS-20 physiological anxiety \| 3.45 \| 4.5 \| \| Mothers’ PASS-20 total score \| 23.67 \| 17.8 \| \| Mothers’ BSI-18 GSI \| 6.39 \| 6.0 \|   **Correlation Results:**  **Girls:**  CPM anticipatory anxiety (0.343; *p* < .01) and CPM pain related fear  (-0.135; *p* < .01) were significantly related to maternal PASS-20 total score.  CPM anticipatory anxiety (0.333; *p* < .01) and CPM pain related fear  (-0.339; *p* < .01) were significantly related to maternal PASS-20 cognitive anxiety score.  **Boys:**  CPM magnitude significantly related to maternal PASS-20 total score (0.431, *p* < .01) and escape/avoidance (0.382, *p* < .01), fearful thinking (0.351, *p* < .01), cognitive anxiety (0.395, *p* < .01), and physiological subscales (0.339, *p* < .01).  **Multivariate Regression Results:**  Separate sequential multiple regressions were performed for each DV and were performed for boys and girls separately.  Step 1 = child age  Step 2 = maternal GSI  Step 3 = Maternal PASS-20 Score  **Girls:**  **Anticipatory Anxiety**  Step 1: child age; NS  Step 2: maternal GSI; NS  Step 3: maternal PASS-20; contributed 9% of the variance (β = 0.325; R^2^ = 0.124; t = 2.45; *p* = 0.02)  Full model: Accounted for 12% of the variance in CPM magnitude (8% adjusted)  **Pain-related fear**  Step 1: child age; NS  Step 2: maternal GSI; NS  Step 3: maternal PASS-20; contributed 9% of the variance in CPM magnitude (β = 0.320; R^2^ = 0.091; *p* < .05)  **CPM Magnitude**  Step 1: child age; NS  Step 2: maternal GSI; NS  Step 3: maternal PASS-20; NS  **Boys:**  **Anticipatory Anxiety**  Step 1: child age; explained 7% of variance in CPM magnitude (β = -0.261; R^2^ = 0.068; *p* < .05)  Step 2: maternal GSI; NS  Step 3: NS  **Pain-related fear**  Step 1: child age; NS  Step 2: maternal GSI; NS  Step 3: maternal PASS-20; NS  **CPM Magnitude**  Step 1: child age; NS  Step 2: maternal GSI; NS  Step 3: maternal PASS-20; NS significant incremental increase in the prediction of child CPM magnitude (β = 0.395; R^2^ = 0.204; t = 3.15; P = 0.00), accounting for an additional 14% of the variance in CPM magnitude.  Full model accounted for 20% (18% adjusted) of variation in CPM magnitude. |
| **Ferland, Teles et al. (2018)** Canada | **Paediatric patients with chronic back pain**  105 patients (22 male). Mean age 15.2 (2.22).  70.2% had adolescent idiopathic scoliosis, 16.3% disc disease, 135.% other including spondylolysis, spina bifida, osteogenesis imperfecta  Participants were aged between 10 and 18 and had reported back pain over the last three months and could understand and respond to outcome measures in French or English.  No patient in the sample was receiving antidepressant or other neuropathic pain medication. The use of out of counter pain medication was not recorded. | **Test stimulus: Thermal pain**  Assessed via 9cm^2^ Peltier thermode applied to the right forearm. Initial temperature was Initially set at 32°C, increasing by 0.3°C per second to reach the target temperature (individual pain intensity rating of 5/10), where it remained constant for 120 seconds. Patients continuously rated their pain using the computerised visual analogue scale (CoVas).  **Conditioning stimulus: Cold pressor test**  Participants immersed their left forearm to approximately two inches above their elbow in a bath of cold water (12°C) for two minutes they rated the pain every 15 seconds.  **Procedure**  Mean pain intensity of the test stimulus before and after the conditioning stimulus. | **Pain Profiles**  A modified version of the Adolescent Pediatric Pain Tool (APPT) was used to evaluate the intensity and quality of pain (sensory, affective, evaluative and temporal descriptors).  The Neuropathic Questionnaire (DN4) consisted of both sensory descriptors and signs related to bedside sensory examination used to identify whether neuronal  **Physical and Emotional Functioning**  Functional Disability Inventory was used to assess physical functioning.  **Sleep quality**  Assessed using the Pittsburgh Sleep Quality Index (PSQI). Measures quality, latency, duration, habitual efficiency, disturbances, use of sleeping medications and daytime dysfunction.  **Emotional functioning**  Evaluated using the Revised Child Anxiety and Depression Scale, a self-report to assess children’s report of symptoms corresponding to selected DSM-IV anxiety disorders and depression. | **CPM and outcome measures**   \|  \| Total (n = 105) \| Male (n = 22) \| Female (n = 83) \| \| --- \| --- \| --- \| --- \| \| Pain intensity (NRS) \| 7 (6, 8) \| 7 (6, 8) \| 7 (5, 8) \| \| APPT \|  \|  \|  \| \| Sensory \| 14 (11, 24) \| 16 (11, 24) \| 11 (5, 18) \| \| Affective \| 0 (0, 9) \| 0 (0, 18) \| 0 (0, 9) \| \| Evaluative \| 25 (15.25, 38) \| 25 (25, 50) \| 25 (12, 38) \| \| Temporal \| 18 (18, 27) \| 18 (18, 29.25) \| 18 (9, 29.25) \| \| DN4, median (P25/P75) \| 2 (1, 4) \| 1 (0, 2) \| 2 (1, 4) \| \| Neuropathic pain \| 26.20% \| 13.60% \| 29.60% \| \| Physical and emotional functioning \|  \|  \|  \| \| RCADS \| 31.90 (16.90) \| 24.38 (16.29) \| 33.85 (16.60) \| \| Anxiety/depression \| 5.90% \| 4.80% \| 6.20% \| \| Functional disability index (FDI) \| 13.07 (8.94) \| 10.23 (7.73) \| 13.83 (9.13) \| \| FDI (classification) \|  \|  \|  \| \| No disability \| 51.00% \| 68.20% \| 46.30% \| \| Mild disability \| 27.90% \| 18.20% \| 30.50% \| \| Moderate disability \| 17.30% \| 13.60% \| 18.30% \| \| Severe disability \| 3.80% \| 0.00% \| 4.90% \| \| PSQI score \| 7.21 (3.19) \| 6.00 (3.08) \| 7.54 (3.16) \| \| Sleep disorder \| 74.80% \| 59.10% \| 79.00% \| \| CPM assessment \|  \|  \|  \| \| CPM (measures) \| 37.0 (14.0–65.0) \| 30.19 (41.54) \| 34.64 (48.97) \| \| Mean pain intensity pre \| 39.64 (20.65) \| 38.47 (22.63) \| 39.94 (20.24) \| \| Mean pain intensity post \| 24.20 (17.41) \| 29.90 (19.06) \| 22.72 (16.76) \|   CPM was not significantly associated with pain quality, functional disability, sleep, or mood disorders. Hierarchal multiple linear regression assessing the effect of age, gender, pain profile and physical and emotional functioning was not significant (*p* = .466). Stepwise regression found no significant association between patient reported measures and CPM.  Overall different correlation coefficients between males and females, with stronger coefficients observed in males. CPM presented a positive and significant correlation with the blood concentrations of DOPA (.499) and 5-HT (.519) in males only. CPM correlated significantly with ME (-0.275), NE (-.254), and NME (-.270) in the entire sample, and also for males (-.763, -.0472, and -0.578 respectively) and females for NE (-0.201) and MNE (-0.217).  A hierarchal multiple linear regression assessing the effect of all monoamines on CPM (controlling for age and gender) was non-significant (*p* = .064). Stepwise regression controlling for age and gender indicated ME (*p* = .002) was the only significant predictor for CPM. Higher blood ME concentration was associated with poorer CPM efficacy. ME explained 53% of variation of CPM in males (*p* < .0001) and 7% in females (*p* = .014).  Across the whole sample, 5-HT was positively correlated with sensory (0.204) and evaluative (0.243) descriptors of quality of pain. A negative correlation was shown between Epi and DN4 (-0.228). A negative correlation was found between NME and FDI in females (-0.222). A positive correlation between DOPA and evaluative quality of pain (0.472) was found in males. No such associations were found after controlling for confounders in the multiple linear regressions. |
| **Goffaux, Lafrenaye et al. (2008)** Canada | 26 children  Aged 7-11  13 premature (<32-week gestation)  6 ‘Low pain’  7 ‘high pain’  (based on median number of days spent in the NICU and under mechanical ventilation as an estimate for number of painful interventions experienced)  13 full-term (>38-week gestation)  Exclusion Criteria:  Chronic pain; taken painkillers prior to testing  **Full Term:**  Age 9.3 ± 1.3  Gestational age 39.0 ± 0.7  Birth weight 3806.4 ± 506.2 g  **Low-pain preterm:**  Age 9.2 ± 1.5  Gestational age 30.5 ± 1.6  Birth weight 1567.0 ± 363.7  **High-pain preterm:**  Age 9.4 ± 1.1  Gestational age 28.6 ± 2.3  Birth weight 1050 ± 282.9 | **Test stimulus: thermal pain**  Applied using 3x3cm Peltier thermode controlled by a Medoc Neuro Sensory Analyzer, Model TSA-II. Pain threshold value was determined by increasing thermode temperature from baseline of 32°C at a rate of 1°C/s until pain was perceived. Thermal pain sensitivity was tested by setting the thermode to 46°C for 5 seconds Global changes in thermal pain sensitivity were tested by repetition on both the left calf and left forearm.  **Conditioning Stimulus: Cold pressor test**  Right hand submerged for 3 min in a bath of cold water kept at 13 °C.  **Procedure**  After participants were fitted with pediatric ECG recording electrodes, their resting  heart rates were measured. Pain threshold and suprathreshold values were then assessed, after which participants immersed their right hand in cold water and immediately repeated thermode testing. | **Pain intensity: visual analogue scale (VAS)**  Participants rated pain intensity using VAS with numeric and verbal descriptors ranging from 0 (no pain) to 10 (most intense pain imaginable).  **Heart Rate: Electrocardiogram activity (ECG)**  3-lead ECG sampled at a frequency of 1000Hz. Baseline was recorded over a 2-minute period prior to testing. Heart rate changes in response to CPM was calculated as percentage difference in heartrate between immersion and baseline periods, computed over 30 second periods successively. | All participants perceived the submersion task as painful;  (*F*(2,23) = 2.41, *p* = 0.11, n2 = 0.17).  High-pain preterm children showed a trend toward increased pain intensity (m = 5.6, SD = 2.4) than low-pain preterm (mean=3.8, SD =1.7) and full-term (m = 4.1, SD = 0.9) children.  Conditioning pain resulted in reduced pain intensity values for the full-term and low-pain preterm groups (*F*s(1,23) > 5.31, *p*s < .03, n2s > .20), but had no effect on the high-pain preterm group (*F*(1,23) = 0.06, *p* = 0.82, n2 = 0.003).  Significant variation in CPM between high-pain and low-pain preterms  (*F*(1,11) = 6.45, p = 0.02) remained when the total number of painful procedures was removed as a covariant (F(1,9) = 5.83, p = 0.04).  Addition of the number of painful procedures experienced during the first week of NICU admission as a covariate resulted in non-significance  (*F*(1,9) = 3.99, p = 0.08).  **Heart Rate Responses:**   \|  \| M \| SD \| \| --- \| --- \| --- \| \| High pain preterm \| 93.3 \| 10.6 \| \| Low pain preterm \| 91.2 \| 8.1 \| \| Full term \| 77.5 \| 9.1 \|   Significant difference in heart rate between groups;  (*F*(2,23) = 8.27, *p* = 0.002, n2 = 0.42).  Significant variation in heart rate increase in response to cold pressor between groups; *F*(10,115) = 2.04, p = 0.03, n2 = 0.15.  High-pain preterms showed minimal HR increase (*p*s > 0.14) whilst low-pain preterms and full terms showed significant increase in HR which remained significant across the first 90seconds of the cold pressor task (*t*s > 2.3, *ps* < .05). |
| **Harper & Hollins (2017)**  USA | **37 healthy undergraduate students**  Randomised to either thermal grill (TG) group or noxious heat (NH) group.  Thermal grill group  n = 18; 6 males  Aged 18 to 22  (mean = 19.1, SD = 1.2)  Noxious heat group  n = 19; 7 males  Aged 18 to 20 mean=19.1, SD=0.9 | **Test Stimulus: Thermal grill**  The thermal grill consisted of 12 copper  tubes (length 33 cm; diameter 1 cm; thickness 0.4 mm). Temperature was modified via water flowing through plastic tubing connected to the ends of the bars. The right volar forearm was tested.  **Conditioning stimulus: Cold pressor test**  The participant’s left hand was immersed into a water bath. The temperature of the water was painfully cold (mean = 6.1 °C; SD = 0.08) during the CPM run and was neutral (mean = 32.7 °C; SD = 0.26) during the control run.  **CPM Testing Procedure**  Participants completed a CPM run and a control run, in counterbalanced order.  The participant placed their right volar forearm onto the grill perpendicular to the long axis of the bars. For participants in the NH group, the grill was controlled to approximately 45 °C. For participants in the TG group, the bars were interlaced at approximately 18 °C and 42 °C to produce the thermal grill illusion. Some variation was reported in temperatures however, as discussed in the results column.  Each run began with the participant placing their left hand in the water bath up to the wrist. Participants provided verbal ratings of pain intensity of the water bath on a 0–100 scale (0 = ‘no pain’, 100 = ‘the most intense pain imaginable’), every 15 seconds for the first 45 seconds. One minute into the trial, participants placed the volar surface of their right forearm onto the grill for five seconds. Participants then provided verbal ratings for  grill pain intensity (0–100) and unpleasantness (0 = ‘not at all unpleasant’, 100 =‘the most unpleasant sensation imaginable’). After the trial, participants completed a sensation questionnaire, circling descriptors which applied to the grill sensations.  A component temperatures experiment was conducted with a separate sample of participants, although details are not reported here. | No additional outcome measures were used. | **Bar temperature (°C)**  For the NH group, the average bar temperatures of sets 1 and 2 were 44.75 (SD = 0.39) and 44.87 (0.41) during the control run respectively. The average temperatures were 44.71 (0.48) and 44.87 (0.41) during the CPM run respectively.  For the TG group, the average bar temperatures of the warm and cool sets were 41.96 (0.37) and 18.18 (0.15) during the control run respectively. The average temperatures were 42.01 (0.39) and 18.22 (0.17) during the CPM run respectively.  **CPM pain intensity ratings (mean and SD)**   \| **Condition** \| **Group** \| **Rating 1 (15 secs)** \| **Rating 1 (30 secs)** \| **Rating 1 (45 secs)** \| **Average bath rating** \| \| --- \| --- \| --- \| --- \| --- \| --- \| \| Control \| Noxious heat \| 1.9 (4.7) \| 1.6 (4.6) \| 1.6 (4.7) \| 1.7 (4.6) \| \|  \| Thermal grill \| 2.2 (3.5) \| 1.9 (3.5) \| 2.0 (3.9) \| 2.1 (3.2) \| \| CPM \| Noxious heat \| 38.8 (24.6) \| 52.1 (27.0) \| 62.5 (26.5) \| 51.1 (25.1) \| \|  \| Thermal grill \| 37.2 (23.5) \| 49.4 (26.5) \| 58.1 (27.7) \| 48.2 (28.0) \|   No significant differences in pain intensity were reported across groups  **Grill pain intensity**  During the control run, the noxious heat and thermal grill conditions produced pain intensity ratings of 35.7 (SD = 25.1) and 27.1 (23.9) respectively. During the CPM run, pain intensity of the noxious heat and thermal grill conditions were reduced by 48.2% (M = 18.5; SD = 20.1) and 47.0% (M = 14.3; SD = 13.5) respectively.  A significant main effect of run type was found, indicating a significant reduction in grill pain intensity by CPM (*F*(1, 35) = 19.8, *p* < .001). The main effect of group (i.e., noxious heat vs. thermal grill) was not significant (*F*(1,35) = 1.1, *p* = .30],nor was the interaction between run type and group two factors was not significant (*F*(1,35) = 0.4, *p* = .51).  To further explore lack of differences for group, post hoc *t*-tests were run. Painfulness of the two grill configurations was not significantly different in the control run (*t*(35) = 1.07, *p* = .29), nor in the CPM run (*t*(35) = 0.73, *p* = .47).  **Grill pain unpleasantness**  Pain unpleasantness in the noxious heat (M = 35.8; SD = 27.1) and thermal grill (M = 31.6; SD = 25.7) conditions during the control runs was reduced during CPM by 55.2%  (M = 16.1, SD = 18.1) and 56.6% (M = 13.7, SD = 14.6) respectively.  A significant main effect of run type was found, indicating a significant reduction in grill pain intensity by CPM (*F*(1, 35) = 26.5, *p* < .001). The main effect of group (i.e., noxious heat vs. thermal grill) was not significant (*F*(1,35) = 0.3, *p* = .61], nor was the interaction between run type and group two factors was not significant (*F*(1,35) = 0.1, *p* = .80).  To further explore lack of differences for group, post hoc *t*-tests were run. Painfulness of the two grill configurations was not significantly different in the control run (*t*(35) = 0.48, *p* = .63), nor in the CPM run (*t*(35) = 0.43, *p* = .67).  **Grill descriptors**  The frequencies of sensations reported for the thermal test stimuli during the CPM run did not significantly differ between noxious heat and thermal grill groups (*p* < .19).  CPM changed perception of the thermal grills (noxious heat and thermal grill groups) in a similar manner, both significantly less often described as *burning* and *hot*, and described significantly more often as *warm* during CPM compared to control run. For the thermal grill group, CPM also significantly reduced *sharp* responses. |
| **Hoehn et al. (2022)** USA | **54 healthy children**  Aged 6-12years (9.05 ± 1.84)  34 male, 20 females.  43 white  6 Black/African American  1 Hispanic/Latino  3 Biracial  8 1^st^ grade  8 2^nd^ grade  9 3^rd^ grade  10 4^th^ grade  6 5^th^ grade  9 6^th^ grade  2 7^th^ grade | **Test Stimulus: Pressure pain threshold**  Pressure pain applied to right thumbnail elicited by algometer at a steady rate via a blunt round probe at 0.5 cm2 in diameter. Pressure increased at a rate of 2N/s with a ceiling pressure of 60N.  Two experimental test trials were conducted, with the mean pain score of the two trials calculated as the participant’s pressure pain threshold.  **Conditioning Stimulus: Cold pressor**  Left hand submerged up to the wrist in a water bath of 12°C. 25 seconds post submersion the first pressure pain threshold was performed. 45 seconds post submersion the second pressure pain threshold was performed.  After a 10-minute break, the CPM task was repeated.  A control condition of non-painful conditioning stimulus was conducted using a water bath circulated at 22°C.  **Procedure**  Baseline pressure pain thresholds were first assessed, followed by the first CPM trial. A ten-minute break was then provided, after which the second baseline measure of pressure pain threshold was taken followed by the second CPM trial. | **Pain intensity: Visual Analogue scale (VAS)**  Participants rated pain intensity using VAS with numeric and verbal descriptors ranging from 0 (no pain) to 10 (most intense pain imaginable).  **Pubertal Development: Pubertal Development Scale, modified for parent-report.**  5-item questionnaire comprising skin changes, changes in height/growth spurt, body hair, breast growth (girls only), voice deepening (boys only), and facial hair (boys only) rated on a four-point Likert type scale  Additional item regarding parents’ perception of pubertal timing compared to peers, rated on a four-point Likert scale (1) much earlier, 2) somewhat earlier, 3) about the same, 4) somewhat later, and 5) much later)  **Anxiety & Pain-related Anxiety: Visual Analogue Scale**  Children rated anxiety in relation to pressure and cold-pressor tasks. Participants marked on a visual 10cm line with anchors of “not at all worried or nervous” at the left end and “very worried or nervous” at the right end. | \|  \| **Mean** \| **SD** \| \| --- \| --- \| --- \| \| Baseline 1 Mean PPT \| 20.28 \| 9.42 \| \| Baseline 2 Mean PPT \| 19.39 \| 8.87 \| \| Mean of 2 Baseline PPTs \| 19.84 \| 8.91 \| \| Painful CPM Mean PPT \| 22.50 \| 10.71 \| \| Nonpainful CPM Mean PPT \| 19.85 \| 9.34 \| \| Duration of hand in cold water (s) \| 42.87 \| 18.77 \|   Painful conditioning stimulus resulted in greater endogenous inhibition (*p* = .009)  Significant linear effect of condition on CPM (χ2 = 12.30, *p* < .001), with greater CPM effects during delivery of the conditioning stimulus.  Significant improvement of model fit when condition included as a quadratic predictor (χ2 = 4.5, *p* < .05)  Significant main effect of age; adding age as a fixed predictor significantly improved model fit (χ2 = 6.5, *p* < .05), with older age predicting higher PPT scores across time points. |
| **Holden, Rathleff et al. (2020)** Denmark | **Patellofemoral pain (PFP) group (*n* =138), Mean (SD)**  Age (years): 12.6 (1.2)  Sex (% female): 76  Height (m): 1.62 (0.1)  Weight (kg): 50.4 (9.4)  Bilateral pain (%): 73.5  Pain duration (months), median (IQR): 18 (9-24)  KOOS Symptoms (0-100): 78.2 (12.2)  KOOS Pain (0-100): 68.5 (1.2)  KOOS function in daily living (0-100): 79.0 (14.3)  KOOS function in sport and recreation (0-100): 55.3 (21.2)  KOOS quality of life (0-100): 49.3 (15.5)  Worst pain in the last week (NRS 0-10): 6.6 (2.2)  **Control group *(n* = 48), Mean (SD)**  Age (years): 12.3 (1.4)  Sex (% female): 62  Height (m): 1.60 (0.1)  Weight (kg): 48 (10.4)  KOOS Symptoms (0-100): 97.7 (5.2)  KOOS Pain (0-100): 99.7 (1.2)  KOOS function in daily living (0-100): 100 (0)  KOOS function in sport and recreation (0-100): 99.8 (1)  KOOS quality of life (0-100): 99.7 (1.3)  KOOS, Knee injury and Osteoarthritis Outcomes Score; NRS, numeric rating scale. | **Test stimulus: Pressure pain**  Assessed using an automated cuff algometer applied to the head of the gastrocnemius muscle on each limb. The cuff was inflated at a rate of 1 kPa/second. Participants used an electronic VAS to indicate when the sensation first changed from pressure to pressure pain, and to continue to rate the pain until they could no longer tolerate it.  **Conditioning stimulus: Pressure pain**  Assessed using an automated cuff algometer applied to the contralateral leg inflated at a rate of 100 kPa/second to a level of 70% of the pressure tolerance thresholds (PTT) and held constant.  **Procedure**  The test stimulus was applied alone, and again during the conditioning stimulus. | **Numerical Rating Scale** Assessing worst pain intensity during the last week  **Knee Injury and Osteoarthritis Outcome Score** Assessing pain, symptoms, function in daily living, function in sport and recreation, and knee-related quality of life  **Cuff pain sensitivity**  Assessed via automated cuff algometer, as described for the test stimulus. The pain detection threshold was defined as the point at which the VAS reached 1 cm, and PTT defined as the point at which participants pressed the button and stopped the stimulation.  **Temporal summation of pain**  Assessed via computerised cuff algometer. The TSP paradigm consisted of 10 sequential stimulations (1-second stimulation, 1-second interval without stimulation) inflated at 100 kPa/second to the level of the PTT. Participants were first exposed to 4 stimulations (60%, 80%, 90%, and 100% PTT) with 5 second intervals. Following this, the TSP paradigm of 10 equal pressure stimuli began, at 100% PTT. Participants rated the pain of each stimulus using the electronic VAS, without returning the slider to zero in-between stimuli. Participants were given no indication as to whether stimuli would be more or less painful. VAS scores were averaged from the first to fourth VAS score (VAS-I) and for the final 3 VAS scores (VAS-II). The TSP effect was defined as the difference between VAS-I and VAS-II (VAS-II minus VAS-I; greater difference indicating more facilitated TSP). | **Mean (95% CI) of cuff pressure detection thresholds (PDT) and tolerance thresholds (PTT) for adolescents with patellofemoral pain (PFP) and controls at baseline.**   \|  \| PFP \| Control \| \| --- \| --- \| --- \| \| CUFF PDT (KPa) \| 25.3 (23.3-27.3)* \| 35.9 (32.3-39.5) \| \| CUFF PTT (KPa) \| 63.9 (59.9-67.9)# \| 74.7 (67.5-82.0) \|   Significantly different from controls (**p* = .001; #*p* = .05).  **Conditioned Pain Modulation**  For CPM, there was a significant condition*group interaction (F (1, 182) 5 5.098; P 5 0.025). Post hoc analysis showed that the control group had an increase in PDT (F (1, 45) 5 5.191; P 5 0.028) during painful conditioning stimulus compared to without conditioning (mean difference 5 5.4 kPa; 95% CI: 0.6-10.2), indicating a CPM response. However, there was no significant change in PDT during conditioning for the PFP group (F (1, 137) 5 0.47; P 5 0.495) indicating no efficient CPM response in the PFP group.  **Mean (95% CI) values for effects of conditioned pain modulation (CPM-effect; change in PDT k from pre-conditioning to during conditioning) and temporal summation of pain (TSP-effect; VAS-II minus VAS-I representing summation) at baseline, 4- and 12-weeks during intervention in the patellofemoral pain group.**   \|  \| CPM-effect \| TSP-effect \| \| --- \| --- \| --- \| \| Baseline \| 0.6 (-1.5 to 2.7) \| 1.5 (1.3-1.7) \| \| 4-week \| -0.3 (-2.4 to 1.8) \| 1.8* (1.6-2.0) \| \| 12-week \| -2.1 (-4.2 to 0.1) \| 1.5 (1.3-1.7) \|   Significantly different from controls (*P , 0.05).  **Univariable linear regression of baseline quantitative sensory testing predicting change in pain form baseline to 12 week.**   \| Baseline variable \| B \| 95% CI B \| Beta \| *p* \| \| --- \| --- \| --- \| --- \| --- \| \| Widespread PPT \| -0.001 \| -0.01 to 0.002 \| -0.63 \| .487 \| \| CPM \| -0.015 \| -0.07 to 0.04 \| -0.051 \| .579 \| \| TSP \| -0.73 \| -1.24 to -0.22 \| -0.254 \| .006 \|   CPM, conditioned pain modulation; PPT, pressure pain thresholds; TSP, temporal summation of pain. |
| **Holden, Straszek et al. (2018)** Denmark | **Current-PFP** (patellofemoral pain) (*n* = 36)  Age (years): 22.8 (1.1)  BMI (kg/m^2^): 24.1 (4.1)  Height (m): 1.69 (0.08)  Weight (kg): 69.2 (13.8)  Test limb (% dominant): 37  Bilateral pain (%): 89%  Pain duration (y): 8 (7-10)  KOOS symptoms (0-100): 71 (16)  KOOS pain (0-100): 67 (13)  KOOS activity (0-100): 78 (13)  KOOS sport (0-100): 48 (21)  KOOS QoL (0-100): 51 (21)  Pain frequency (%): Daily: 34%. Several times per week: 34%. Weekly: 17%. Monthly: 14%. Rarely: 0%. Never: 0%. Current pain (NRS 0-10): 2 (2)  Worst pain in the past 4 weeks (NRS 0-10): 7 (2)  Average pain in the past 4 weeks (NRS 0-10): 4 (1)  **Recovered-PFP** (*n* = 22)  Age (years): 23.2 (1.2)  BMI (kg/m^2^): 23.7 (4.0)  Height (m): 1.66 (0.06)  Weight (kg): 65.3 (10.5)  Test limb (% dominant): 54  Bilateral pain (%): 77%  Pain duration (y): 5 (2.9-6.6)  Time since knee pain (y): 2 (0.7-4.0)  KOOS symptoms (0-100): 95 (5)  KOOS pain (0-100): 97 (4)  KOOS activity (0-100): 98 (2)  KOOS sport (0-100): 91 (11)  KOOS QoL (0-100): 85 (13)  **Healthy control** (n = 29)  Age (years): 23.1 (1.2)  BMI (kg/m^2^): 22.7 (4.1)  Height (m): 1.67 (0.06)  Weight (kg): 63.3 (11.1)  Test limb (% dominant): 41  KOOS symptoms (0-100): 97 (3)  KOOS pain (0-100): 100 (1)  KOOS activity (0-100): 100 (2)  KOOS sport (0-100): 99 (2)  KOOS QoL (0-100): 98 (4) | **Test stimulus: Pressure pain**  Assessed using an automated cuff algometer applied to the head of the gastrocnemius muscle on each limb. The cuff was inflated at a rate of 1 kPa/second to a maximum of 100 kPa. Participants used an electronic VAS to indicate when the sensation first changed from pressure to pressure pain, and to continue to rate the pain until they could no longer tolerate it.  **Conditioning stimulus: Pressure pain**  Assessed using an automated cuff algometer applied to the contralateral leg inflated at a rate of 100 kPa/second to a level of 70% of the pressure tolerance thresholds (PTT) and held constant.  **Procedure** The test stimulus was applied alone, and again during the conditioning stimulus. | **Numerical Rating Scale** Assessing worst pain and average pain intensity during the last week  **Pain location** Assessed via Navigate Pain app  **Knee Injury and Osteoarthritis Outcome Score** Assessing pain, symptoms, function in daily living, function in sport and recreation, and knee-related quality of life  **Pressure pain threshold**  Assessed via handheld algometer with a 1-cm^2^ probe, with pressure increased at a rate of 30 kPa/s. Participants indicated when the sensation changed from pressure pain. The following sites were tested: the knee at the centre of the patella on the test leg; the tibialis anterior muscle 5 cm distal to the tibial tuberosity on the test leg; and the contralateral elbow, on the lateral epicondyle of the humerus.  **Cuff pain sensitivity**  Assessed via automated cuff algometer, as described for the test stimulus. The pain detection threshold was defined as the point at which the VAS reached 1 cm, and PTT defined as the point at which participants pressed the button and stopped the stimulation. This procedure was repeated bilaterally.  **Temporal summation of pain** Assessed via computer-controlled cuff algometer. Ten stimuli (1 second each) at the level of the cuff pressure tolerance threshold were applied with a one second break between stimuli. Participants continuously rate the pain intensity of the stimuli using an electronic VAS, with the slide not returning to zero between stimuli. For analysis of TSP, the average VAS score was calculated in the interval from the first to the fourth VAS score (VAS-I) and for the final 3 VAS scores (VAS-II). The TSP effect was defined as the difference between VAS-I and VAS-II (VAS-II minus VAS-I). | **Conditioned Pain Modulation**  Participants with current-PFP pain had a reduced CPM effect relative to the recovered-PFP (*p* < .005). No other significant between-group differences were found.  **Temporal Summation of Pain** The current-PFP and recovered-PFP groups had a facilitated TSP effect compared with controls (*p* < .01 and *p* < .05 respectively).  **Differences in threshold values on the test leg at baseline and during conditioning, and in VAS during temporal summation paradigm.**   \|  \| Pressure detection threshold \| \| Pressure tolerance threshold \| \| Temporal summation of pain \| \| \| --- \| --- \| --- \| --- \| --- \| --- \| --- \| \|  \| Baseline \| During conditioning \| Baseline \| During conditioning \| Baseline \| During conditioning \| \| Current PFP \| 19.3 (17.0-21.7) \| 25.4 (21.6-29.3)* \| 42.0 (37.4-46.5) \| 47.2 (42.0-52.5)* \| 4.0 (3.4-4.7) \| 5.8 (4.9-6.69 \| \| Recovered PFP \| 24.0 (19.9-28.1) \| 34.2 (28.2-40.2)* \| 53.0 (43.9-62.1) \| 65.8 (64.6-77.0)* \| 3.6 (2.8-4.5) \| 5.3 (4.5-6.1) \| \| Controls \| 25.1 (21.4-28.1) \| 35.9 (30.6-41.2)* \| 54.8 (48.6-61.1) \| 65.3 (57.4-73.2)* \| 4.3 (3.5-5.1) \| 5.2 (4.3-6.1) \|   * Indicates significant increase from baseline. PFP, patellofemoral pain; VAS, visual analogue scale.  **Mean (95% CI) pressure pain thresholds (PPTs), cuff pressure pain detection threshold (PDT), and pressure tolerance thresholds (PTTs) for females with current PFP, recovered PFP, and pain-free controls.**   \|  \| Current PFP \| Recovered PFP \| Controls \| \| --- \| --- \| --- \| --- \| \| Cuff PDT \| 21.1 (18.0-24.18) \| 24.5 (20.1-28.4) \| 25.8 (22.3-29.2) \| \| Cuff PTT \| 42.2 (36.3-48.1)*† \| 52.9 (45.3-60.4) \| 55.0 (48.5-61.6) \| \| PPT centre for patella \| 377.3 (318.3-436.2)*† \| 492.3 (420.3-564.3)† \| 602.8 (534.0-671.7) \| \| PPT tibialis anterior \| 323.2 (262.2-384.1)† \| 398.5 (332.1-464.8) \| 479.8 (410.1-549.4) \| \| PPT contralateral elbow \| 363.3 (262.3-384.1)† \| 423.2 (332.1-464.8) \| 448.7 (410.1-549.4) \|   * Significantly lower than recovered-PFP (*p* < .05); † Significantly lower than controls ( *p* < .05). |
| **Jørgensen, Werner et al. (2024)** Denmark | **Children and adolescents with cerebral palsy (CCP)**  25 children and adolescents with cerebral palsy. Nine with and 16 without chronic pain.  **CCP all (*n* = 25)**  *Age*, median (range): 11 (7-17)  *Female/male*, *n*: 5/20  *BMI*, median (range): 18.5 (13.2-28.7)  *Dominant hand*, *n* right/left/unknown: 15/8/2  *Primary test site*:  Hand *n* (%), right/left, *n*: 8 (32), 3/5  Foot *n* (%), right/left, *n*: 17 (68), 8/9  Pain scale, *n* WBS/NRS: 10/15  **CCP without chronic pain (*n* = 16)**  *Age*, median (range): 11 (7-17)  *Female/male*, *n*: 5/11  *BMI*, median (range): 18.7 (13.2-28.7)  *Dominant hand*, *n* right/left/unknown: 11/3/2  *Primary test site*:  Hand *n* (%), right/left, *n*: 8 (50), 3/5  Foot *n* (%), right/left, *n*: 8 (50), 2/6  Pain scale, *n* WBS/NRS: 7/9  **CCP with chronic pain (*n* = 9)**  *Age*, median (range): 10 (7-17)  *Female/male*, *n*: 0/9  *BMI*, median (range): 17.5 (16-21)  *Dominant hand*, *n* right/left/unknown: 4/5/0  *Primary test site*:  Hand *n* (%), right/left, *n*: 0 (0), 0/0  Foot *n* (%), right/left, *n*: 9 (100), 6/3  Pain scale, *n* WBS/NRS: 3/6  **Typically developed children and adolescents (TDC)**  26 typically developed children and adolescents. Fourteen with and 12 without chronic pain.  **TDC all (*n* = 26)**  *Age*, median (range): 12 (7-16)  *Female/male*, *n*: 16/10  *BMI*, median (range): 17.1 (14.2-28)  *Dominant hand*, *n* right/left/unknown: 13/4/9  *Primary test site*:  Hand *n* (%), right/left, *n*: 8 (31), 1/7  Foot *n* (%), right/left, *n*: 18 (69), 7/11  Pain scale, *n* WBS/NRS: 4/22  **TDC without chronic pain (*n* = 12)**  *Age*, median (range): 9.5 (7-14)  *Female/male*, *n*: 6/6  *BMI*, median (range): 16.8 (15.1-17.6)  *Dominant hand*, *n* right/left/unknown: 9/1/2  *Primary test site*:  Hand *n* (%), right/left, *n*: 6 (50), 0/6  Foot *n* (%), right/left, *n*: 6 (50), 0/6  Pain scale, *n* WBS/NRS: 4/8  **TDC with chronic pain (*n* = 14)**  *Age*, median (range): 12 (9-16)  *Female/male*, *n*: 10/4  *BMI*, median (range): 19.2 (14.2-28)  *Dominant hand*, *n* right/left/unknown: 4/3/7  *Primary test site*:  Hand *n* (%), right/left, *n*: 2 (14), 1/1  Foot *n* (%), right/left, *n*: 12 (86), 7/5  Pain scale, *n* WBS/NRS: 0/14 | **Test stimulus: Pressure pain**  Pressure pain threshold assessed via hand-held algometer with a 1-cm^2^ tip. Pressure was applied manually, increasing by 10–20 kPa/s with a cut-off at 1000 kPa  **Conditioning stimulus: Cold pressor test**  Participants submerged the hand or foot in cold circulating water with a constant temperature of 10.0 ± 0.2◦C for one minute.  **Procedure** The test stimulus was applied alone, and again after 90 seconds of immersion in the cold water.  For all participants expect TDC without chronic pain, the primary test site was the most affected limb. The non-dominant hand or foot site was randomly chosen in TDC without chronic pain. | **Pain Rating Scales**  The Wong-Baker Faces Pain Rating Scale (WBS) and an 11-point Numeric Rating Scale (NRS) were used to assess pain or discomfort during the test procedures  **Reaction Time Test**  Online RED LIGHT-GREEN LIGHT Reaction Time Test  **Detection Thresholds**  Warm and cool detection thresholds performed three times on each site via thermal stimulator with a 25 × 25 mm^2^ thermode , with a thermal ramp of ±1 degrees C/s. Mechanical detection threshold (MDT) was performed five times on each test site using calibrated von Frey polyamide mono- filaments. Vibration detection threshold (VDT) was performed three times on each test site by using a tuning fork placed perpendicularly to the skin.  **Pain Thresholds**  Heat pain threshold (HPT) and heat pain intensity (HPT intensity) assessed using the same thermal stimulator as above. Pressure pain threshold (PPT) and pressure pain intensity (PPT intensity) performed once on each test site using a hand-held algometer with a 1 cm^2^ probe. Pressure was applied at 10–20 kPa/s with a cut-off at 1000 kPa, and.  Mechanical pain threshold (MPT) performed five times on each test site using weighted pins (8–512 mN) on a circular stimulation area of 0.049 mm^2^.  **Dynamic tests**  Wind-up ratio (i.e., temporal summation) assessed via the weighted pins described above. The pin one ordinal number below the MPT was applied with 1 Hz (1/s) for 30 seconds, with the test performed once on each site. Dynamic mechanical allodynia (DMA) was assessed via a brush stroke for 30 seconds (stroke velocity 1 cm/s, stroke distance 1–1.5 cm), performed once on each site The test was performed one time on every test site. For both tests, participants rated the pain on their chosen scale after 15 and 30 seconds of repetitive stimulation. | **Conditioned pain modulation**  Across all participants, pressure pain threshold consistently increased by a median of approximately 30 kPa from unconditioned to conditioned test stimuli. No other effects were found.  **Median and 95CI (unless otherwise stated)**   \| **Reaction times** \| \| \| --- \| --- \| \| CCP all: 0.41 (0.35 - 0.46) CCP without pain: 0.41 (0.35-0.49)  CCP with chronic pain: 0.41 (0.34-0.46) \| TDC all: 0.34 (0.31-0.37)  TDC without chronic pain: 0.37 (0.29-0.44)  TDC with chronic pain: 0.33 (0.31-0.35) \| \| **MDT** \| \| \| CCP all: 2.8 (2.4-3.2) CCP without pain: 2.8 (2.4-3.6)  CCP with chronic pain:2.8 (2.4-3.2) \| TDC all: 2.8 (2.4-2.8)  TDC without chronic pain: 2.8 (2.4-2.8)  TDC with chronic pain: 2.8 (2.4-2.9) \| \| **WDT** \| \| \| CCP all: 3.3 (2.6-4.1) CCP without pain: 3.2 (2.6-5.1) CCP with chronic pain: 3.5 (2.5-7.0) \| TDC all: 3.1 (2.2-5.5)  TDC without chronic pain: 2.1 (1.4-4.0)  TDC with chronic pain: 5.5 (2.8-8.4) \| \| **CDT** \| \| \| CCP all: 1.9 (1.3-3.2) CCP without pain: 1.9 (1.1-5.8)  CCP with chronic pain: 1.9 (1.4-4.1) \| TDC all: 1.3 (1.1-1.5)  TDC without chronic pain: 1.3 (0.9-1.6)  TDC with chronic pain: 1.3 (1.0-2.9) \| \| **HPT** \| \| \| CCP all: 40.5 (38.5-43.7) CCP without pain: 42.1 (38.5-45.7)  CCP with chronic pain: 39.3 (37.5-41.3) \| TDC all: 42.5 (38.7-44.6)  TDC without chronic pain: 38.7 (36.8-44.0)  TDC with chronic pain: 44.2 (42.3-46.0) \| \| **Pain intensity (n, %) of HPT** \| \| \| WBS <5, NRS <7  CCP all: 7 (35) CCP without pain: 5 (45)  CCP with chronic pain: 2 (22)  WBS > 4, NRS > 6  CCP all: 13 (65) CCP without pain: 6 (55)  CCP with chronic pain: 7 (78) \| WBS <5, NRS <7  TDC all: 15 (58)  TDC without chronic pain: 10 (83)  TDC with chronic pain: 5 (36)  WBS > 4, NRS > 6  TDC all: 11 (42)  TDC without chronic pain: 2 (17)  TDC with chronic pain: 9 (64) \| \| **PPT** \| \| \| CCP all: 168 (150.3-209.8) CCP without pain: 166 (126.8-221.5)  CCP with chronic pain: 168 (131.3-265.3) \| TDC all: 189 (140.7-233.1)  TDC without chronic pain: 140 (92.8-212.3)  TDC with chronic pain: (214.5 (166.8-305.3) \| \| **Pain intensity (n, %) of PPT** \| \| \| WBS <5, NRS <7  CCP all: 14 (66) CCP without pain: 9 (56)  CCP with chronic pain: 5 (56)  WBS > 4, NRS > 6  CCP all: 11 (44) CCP without pain: 7 (44)  CCP with chronic pain: 4 (44) \| WBS <5, NRS <7  TDC all: 14 (54)  TDC without chronic pain: 7 (58)  TDC with chronic pain: 7 (50)  WBS > 4, NRS > 6  TDC all: 12 (46)  TDC without chronic pain: 5 (42)  TDC with chronic pain: 7 (50) \| \| **PPT2 (during conditioning stimulus)** \| \| \| CCP all: 210 (156.5-251.1) CCP without pain: 201.5 (141.8-407.7)  CCP with chronic pain: 223 (135.5-291.7) \| TDC all: 213.5 (154.1-263.5)  TDC without chronic pain: 154 (129.7-221.6)  TDC with chronic pain: 255.5(190.6-311.8) \| \| **Pain intensity (n, %) of PPT2** \| \| \| WBS <5, NRS <7  CCP all: 16 (64) CCP without pain: 10 (62)  CCP with chronic pain: 6 (67)  WBS > 4, NRS > 6  CCP all: 9 (36) CCP without pain: 6 (36)  CCP with chronic pain: 3 (33) \| WBS <5, NRS <7  TDC all: 18 (69)  TDC without chronic pain: 9 (75)  TDC with chronic pain: 9 (64)  WBS > 4, NRS > 6  TDC all: 8 (31)  TDC without chronic pain: 3 (25)  TDC with chronic pain: 5 (36) \| \| **VDT** \| \| \| CCP all: 7 (6.0-7.0) CCP without pain: 6.5 (6-7.4)  CCP with chronic pain: 7 (5.1-8.0) \| TDC all: 7 (6.0-7.0)  TDC without chronic pain: 7 (6.2-7.0)  TDC with chronic pain: 6 (5.0-7.0) \| \| **MPT** \| \| \| CCP all: 128 (128-138) CCP without pain: 128 (128-348.5)  CCP with chronic pain: 128 (72.8-128) \| TDC all: 128 (99.1-256)  TDC without chronic pain: 96 (64-233.9)  TDC with chronic pain: 192 (128-256) \| \| **WUR pain intensity at 30 seconds (n, %)** \| \| \| WBS <5, NRS <7  CCP all: 15 (60) CCP without pain: 9 (56)  CCP with chronic pain: 6 (67)  WBS > 4, NRS > 6  CCP all: 3 (12) CCP without pain: 1 (6)  CCP with chronic pain: 2 (22) \| WBS <5, NRS <7  TDC all: 20 (76)  TDC without chronic pain: 10 (67)  TDC with chronic pain: 10 (70)  WBS > 4, NRS > 6  TDC all: 4 (16)  TDC without chronic pain: 1 (25)  TDC with chronic pain: 3 (21) \| \| **DMA pain intensity at 30 seconds (n, %)** \| \| \| WBS <5, NRS <7  CCP all: 3 (12) CCP without pain: 1 (6)  CCP with chronic pain: 2 (22)  WBS > 4, NRS > 6  CCP all: 1 (4) CCP without pain: 0 (0)  CCP with chronic pain: 1 (11) \| WBS <5, NRS <7  TDC all: 3 (11)  TDC without chronic pain: 2 (17)  TDC with chronic pain: 1 (7)  WBS > 4, NRS > 6  TDC all: 2 (8)  TDC without chronic pain: 0 (0)  TDC with chronic pain: 2 (14) \| \| **CPT time to pain (seconds)** \| \| \| CCP all: 11.5 CCP without pain: 12  CCP with chronic pain: 11 \| TDC all: 12  TDC without chronic pain: 11  TDC with chronic pain: 13 \|   **Comparison between all TDC and CCP (regardless of pain status)**  Reaction Times: All CCP had a median reaction time that was 70ms higher than the median reaction time in all TDC (*p* = .010).  **Comparisons across pain status for TDC and CCP**  In TDC with chronic pain, WDT and HPT were both significantly higher than TDC without chronic pain (*p* =.008 and *p* = .02 respectively). Significantly more TDC with chronic pain reported high HPT intensity (WBS >4 or NRS >6) than TDC without chronic pain (*p* = .01). TDC with chronic pain had significantly higher PPT and PPT2 than TDC without chronic pain (both *p* =.03).  **Comparisons between all CCP and TDC with chronic pain and controls**  In all adolescents with CCP, MDT, WDT and CDT were all significantly higher than in TDC without chronic pain (*p* = .05, *p* = .03 and *p* = .03 respectively) as was the proportion of participants with high HPT intensity (*p* = .008). When comparing all CCP with TDC with chronic pain, significantly lower HPT was found in the former than the latter (*p* = .04). |
| **Leone, Caterina et al. (2021)** Italy | **Two groups – Non-suicidal self-injury (NSSI) participants vs healthy controls**  NSSI participants  N=30 (27 female)  Average age was 15.7 years (11–18)  Diagnosis  11 depressive disorder  8 anxiety disorder  4 bipolar disorder  4 behavioural disorder  3 eating disorder  Healthy controls  N=20 (18 female)  Healthy age and sex matched friends / family  16.8 years (11–18) | **Test Stimulus: Heat pain intensity**  Assessed via 30 × 30 Peltier thermode. Contact heat applied to the non-dominant volar forearm (dermatome C5, C6). A baseline temperature of 32°C was applied, which was increased at a rate of 2C°/s. Participants indicated as soon as they perceived a painful sensation with an intensity of at least 60 on a 0–100 NRS scale for three consecutive trials. The average of the three trials was considered the target temperature (pain-60) for the test stimulus.)  **Conditioning Stimulus: Heat pain**  Assessed via 30 × 30 Peltier thermode applied to the dominant forearm (dermatome C8, T1). Heat stimulus lasting 60 seconds using same temperature as test stimuli (predetermine Pain-60)  **Procedure**  Test stimuli intensity measured before and during the conditioning stimuli. Using the ramp and hold method, two consecutive heat stimulations were applied using ‘pain-60’ as target temperature to the non-dominant forearm. The conditioning stimulus was applied, during the last 30 seconds of which two test stimuli were applied consecutively and their intensity rated. | **Thermal Pain Thresholds**  Assessed via 30 × 30 Peltier thermode on the right-hand dorsum. Baseline temperature of 32°C reached target temperature at a ramp rate of 1°C/s. Cold detection threshold (CDT), warm detection threshold (WDT), the cold pain threshold (CPT) and heat pain threshold (HPT) were measured.  **Laser evoked potentials**  The dorsum of the right hand was stimulated by laser pulses (intensity, 89–140 mJ/mm2, 2x individual pinprick threshold; duration, 5 ms; diameter, 5 mm) eliciting pinprick sensations. The interstimulus interval was varied between 10–15 seconds. The target was shifted after each stimulus. The perceptive threshold was defined as the lowest intensity at which the participants perceived at least 50% of laser stimuli, and the pinprick threshold as the lowest intensity at which participants perceived a clear pinprick sensation.  EEG was recorded using 32 Ag–AgCl scalp electrodes placed according to the International 10–20 system. nElectroculographic (EOG) signals were simultaneously recorded using surface electrodes. Separate average waveforms were computed for each participant.  **NSSI group only completed psychopathological instruments:**  Deliberate Self-Harm Inventory (DSHI)  Repetitive Non-Suicidal Self-Injury Questionnaire (R- NSSI-Q)  Clinician-rated severity of non-suicidal self-injury (CRS-NSSI)  Child Depression Inventory (CDI)  Beck Hopelessness Scale (BHS)  Personality Inventory for DSM-5 (PID-5). | **NSSI participants**   \|  \| M \| SD \| \| --- \| --- \| --- \| \| CDT (°C from baseline) \| 4.5 \| 5.06 \| \| WDT (°C from baseline) \| 3.8 \| 3.04 \| \| CPT (°C) \| 9.05 \| 8.52 \| \| HPT (°C) \| 46.51 \| 3.01 \| \| Laser pinprick threshold (mj/mm^3^) \| 82.23 \| 18.49 \| \| NRS pinprick \| 3.42 \| 1.7 \| \| LEP N1 Amplitude (μV) \| 6.27 \| 5.5 \| \| LEP N2 Amplitude (μV) \| 16.75 \| 10.51 \| \| LEP P2 Amplitude (μV) \| 29.39 \| 8.7 \| \| CPM (NRS 0-100) Last minus first \| -0.98 \| 9.25 \|   **Healthy volunteers**   \|  \| M \| SD \| \| --- \| --- \| --- \| \| CDT (°C from baseline) \| 3.6 \| 2.3 \| \| WDT (°C from baseline) \| 3.13 \| 1.77 \| \| CPT (°C) \| 10.83 \| 8.79 \| \| HPT (°C) \| 45.3 \| 4.34 \| \| Laser pinprick threshold (mj/mm^3^) \| 80.7 \| 15.1 \| \| NRS pinprick \| 3.25 \| 1.5 \| \| LEP N1 Amplitude (μV) \| 6.74 \| 5.14 \| \| LEP N2 Amplitude (μV) \| 23.5 \| 11.1 \| \| LEP P2 Amplitude (μV) \| 27.55 \| 15.27 \| \| CPM (NRS 0-100) Last minus first \| -9.92 \| 11.32 \|   LEP N2 amplitude was significant higher in the NSSI group than the healthy group (*p* = .02).  The CPM effect significantly higher in the control group compared to the NSSI group (*p* = .006). Group means suggest intact endogenous pain modulation in healthy controls, and a deficit in the descending inhibitory pain control in the NSSI group. Analysis of variance (ANOVA) of the CPM variable showed disclosed a significant difference between control versus suicidal groups (*p* < .04) and control versus non-suicidal groups (*p* < .004). No significant difference was found between suicidal versus non-suicidal groups(*p* > 0.3).  No significant correlations were found between variables in the NSSI group. |
| **Lucas, Talih et al. (2024)** Portugal | **1727 children and adolescents; 845 females.**  Bullying involvement at age 10  Bullying profiles distribution, *n* (%)  Not involved: 728 (42.2)  Victim only: 445 (25.8)  Both victim and aggressor: 486 (28.1)  Aggressor only: 68 (3.9)  Adverse childhood experiences at age 10  Number of reported ACEs, *n* (%)  0-3A: 935 (54.1)  4-5: 487 (28.2)  6 or more: 305 (17.7) | **Test stimulus: Pressure pain** Assessed via cuff pressure algometry on the right leg for analysis. Pressure pain detection and pain tolerance thresholds were both assessed. The cuff was inflated at a rate of 1kPa/s. Participants indicated once the pressure became painful using an electronic slider on a VAS, and to continued rating the pain until the sensation was no longer tolerable. Pain detection threshold was defined as the pressure value when the VAS moved 1cm. Pain tolerance threshold was defined as the pressure when participants moved the VAS to 10cm.  **Conditioning stimulus: Pressure pain** Assessed via cuff pressure algometry on the left leg, set to 70% of the pain tolerance threshold.  **Procedure** Test pressure pain threshold was assessed before and during the conditioning stimulus. | **Pain Sensitivity**  Pressure pain detection and pain tolerance thresholds as described  **Temporal summation of pain (TSP)**  Assessed via cuff pressure algometry. Ten sequential pressure stimuli were applied on the right leg with an intensity equal to the pain tolerance threshold, with a one-second duration and one-second interval. Participants rated their perceived pain by adjusting the VAS, with scores for each stimulus extracted. Stimuli 1–4 (VAS-I) and 8–10 (VAS-II) were averaged. The TSP effect was computed as the difference between VAS-I and VAS-II (i.e. VAS-II minus VAS-I).  **Bullying** Bullying involvement assessed via the Bully Scale Survey  **Adverse childhood experiences** Lifetime exposure to adverse childhood experiences (ACE) assessed via items adapted from the ACEs study. | **QST Responses at Age 13, mean (SD)**  Pain detection thresholds (PDT; kPa): 21.0 (12.2)  Pain tolerance threshold (PTT; kPa): 52.6 (20.1)  Pain intensity VAS-I (mm): 3.8 (2.2)  Pain intensity VAS-II (mm): 4.8 (2.4)  Temporal summation of pain (difference between VAS-II and VAS-I) (mm): 1.0 (1.1)  Conditioned minus baseline PDT (kPa): 11.3 (14.8)  Conditioned minus baseline PTT (kPa): 9.4 (11.1)  **Linear regression coefficients and 95% CIs for the associations between exposure to different bullying profiles measured at age 10 and cuff pressure algometry responses at age 13**   \| Cuff pressure algometry responses at age 13 \| Bullying profiles at age 10 (reference category: not involved) \| \| \| \| --- \| --- \| --- \| --- \| \|  \| Victim only \| Both victim and aggressor \| Aggressor only \| \| Pain detection thresholds (kPa) \| -1.81 (−3.29, −.33) \| .57 (−.97, 2.12) \| 2.03 (−1.00, 5.07) \| \| Pain tolerance threshold (kPa) \| -2.73 (−5.17, −0.29) \| .01 (−2.54, 2.56) \| 4.88 (−.13, 9.89) \| \| Pain intensity VAS-I (mm) \| .37 (.07, .68) \| .04 (−.28, .35) \| -.20 (−.85, .44) \| \| Pain intensity VAS-II (mm) \| .39 (.06, .72) \| -.01 (−.35, .32) \| -.04 (−.73, .66) \| \| Temporal summation of pain (difference between VAS-II and VAS-I) (mm) \| .02 (−.14, .17) \| -.05 (−.21, .11) \| .17 (−.16, .49) \| \| Conditioned minus baseline PDT (kPa) \| -1.00 (−3.37, 1.38) \| -1.42 (−3.96, 1.12) \| 5.17 (.09, 10.26) \| \| Conditioned minus baseline PTT (kPa) \| -.05 (−1.82, 1.73) \| -.36 (−2.26, 1.54) \| -.95 (−4.75, 2.86) \| |
| **Morris, Walker et al. (2015)** USA | **78 healthy children**  Aged 10-17 years  N = 40 African American  Age 14.6 ± 2.0  20 male  20 female  19 post menarchal females  Socioeconomic status 34.5 ± 13.0  N = 38 Non-Hispanic White  Age 15.0 ± 1.6  18 male  20 female  17 post menarchal females  Socioeconomic status 40.0 ± 12.7 | **Test stimulus: thermal pain**  Delivered by 30x30mm thermode applied to the ventral forearm of the non-dominant hand. Administered using Medoc TSA-II Neurosensory Analyzer  **Conditioning Stimulus: hot water immersion**  Dominant hand submerged in a water bath circulating at 46.5°C.  **Procedure**  Thermode applied to the non-dominant ventral forearm for 15 second periods at 45 °C, 46 °C, and 47 °C, and at additional lower or high temperatures as needed until ‘pain-6’ was identified. Three pre-conditioning pain ratings recorded at a non-overlapping test site for 30-seconds at 10-second intervals. Participants took a 10-minute break. Participants submerged their dominant hand in the hot water for 60 seconds during which they provided three pain ratings at 0, 10 and 20 seconds. At 30 seconds, a single 30 second pain-6 heat pulse was applied to the non-dominant arm and participants provided conditioning pain ratings at 40, 50 and 60 seconds. | **Pain Intensity: Numerical Rating Scale (NRS)**  0-10 scale (0 = ‘no pain’, 10 = ‘worst imaginable pain’).  **Somatic Symptoms: Children’s Somatization Inventory revised form (CSI)**  Participants reported how much they were bothered by 24 somatic symptoms on a 5-point scale ranging from 0 (‘not at all’ to 4 (‘a whole lot’).  **Functional Disability: The Functional Disability Inventory**  Measured perceived impact of general physical health on psychosocial and physical functioning. Participants reported the degree of difficulty of 10 different tasks on a 5-point scale. Items were summed to provide an overall functional disability score.  **Pain Catastrophising: Pain Catastrophizing Scale for Children  (PCS-C)**  13-item self-report questionnaire. Participants reported the degree of difficulty they would have performing 15 specific activities due to their physical health on a 5-point scale ranging from 0 to 4.  **Anxiety & Depressive symptoms: PROMIS depressive and anxiety symptom scales**  Participants reported how frequently they experienced 8 anxiety and 8 depressive symptoms on 5-point scales ranging from 0 (“never”) to 5 (“almost always”). | \| **M (SD)** \| **African American** \| **Non-Hispanic Whites** \| \| --- \| --- \| --- \| \| Pain-6 temperature \| 46.3 (1.8) \| 47.3 (1.3) \| \| Pre-conditioning \| 5.6 (1.5) \| 6.2 (1.2) \| \| CPM trial 1 \| 5.4 (2.0) \| 5.7 (1.7) \| \| CPM trial 2 \| 4.2 (2.2) \| 5.1 (1.7) \| \| CPM trial 3 \| 3.4 (2.2) \| 4.7 (1.5) \|   African American participants showed significantly lower Pain-6 temperatures; (*t* = 2.81, *p* < .01)  Average conditioning pain rating (*m* = 4.8) declined significantly from their average pre-conditioning pain rating (*m* = 5.9); (*t* = 5.4, *p* < .01)  African Americans reported lower average conditioning pain ratings than non-Hispanic whites; (*t* = 2.4, *p* = .02)  Race significantly associated with mean conditioning pain when controlling for mean pre-conditioning pain; *F* = 4.0, *p* = .049). African American children showed greater CPM effect than Non-Hispanic White children.  Significant Race x Trial interaction; *b* = .33, *SE* = .13, *p* = .02; Simple effects showed pain ratings decreased faster in African American children (*b* = −.80, *SE* = .09, *p* < .01), than for Non-Hispanic white children (*b* = −.47, *SE* = .10, *p* < .01).  Pain ratings decrease in children with lower somatisation scores  (*b* = −.46, *SE* = .10, *p* < .01) but did not vary significantly in children with higher somatisation scores (*b* = .08, *SE* = .29, *p* = .78)  Child somatisation was associated with changes in pain rating;  (*b* = .18, *SE* = .07, *p* = .01). |
| **Morris, Walker et al. (2016)** USA | **Functional Abdominal Pain Group  (n = 63)**  Aged 11-17 (14.4 ± 1.9)  21 Male, 42 Female  53 Caucasian, 10 non-Caucasian  Somatic symptoms 30.8 ± 16.0  Functional Disability 11.3 ± 7.4  A significant proportion of FAP participants met criteria for a pain-related functional gastrointestinal disorder:  IBS (n = 39)  functional dyspepsia (n = 4)  IBS and functional dyspepsia (n = 3)  Eligibility: recurrent abdominal pain for at least the past 2 months and could read and write in English at least sixth grade level.  **Healthy Controls (n = 77)**  Aged 10-17 (14.8 ± 1.8)  38 Male, 39 Female  37 Caucasian, 40 Non-Caucasian | **Test stimulus: Thermal Pain**  Delivered using 30x30mm thermode applied to participant’s non-dominant ventral forearm. Computer software Medoc TSA-II Neurosensory Analyser & TPS-CoVAS version 3.19 was used to apply the test stimulus.  **Conditioning Stimulus: Hot water immersion**  Dominant hand submerged in a water bath with a 46.5°C temperature.  **Procedure**  P-60 determined via repeat 15 second application of thermode to the non-dominant ventral forearm at 45, 46 and 47°C and higher/lower temperatures until P-60 was identified. Thermode moved to non-overlapping area on the nondominant ventral forearm, and the test stimulus was applied continuously for 30s and three pain ratings were taking at each 10s interval. A ten-minute break was then given. After this, the dominant hand was immersed in conditioning water bath for 60s and pain ratings were taken at 0, 10 and 20s.  At 30 seconds immersion, the test stimulus was applied at the participant’s P-60 for 30 seconds with 3 pain ratings taken at each 10 second interval. | **Pain intensity: Numerical rating scale (NRS)**  Participants reported pain intensity on a 0 (“no pain”) to 100 (“worst pain imaginable”) scale.  **Somatic Symptoms: The Children’s Somatization Inventory - revised form (CSI).**  Measured somatic symptoms including headache, dizziness, nausea and back pain in the past 2-weeks. Participants report how bothered they were by each symptom on a 5-point scale (0 = ‘not at all’, 4 = ‘a whole lot’). Items were summed to create an overall somatic symptom score.  **Functional Disability: The Functional Disability Inventory**  Measured perceived impact of general physical health on psychosocial and physical functioning. Participants reported the degree of difficulty of 10 different tasks on a 5-point scale. Items were summed to provide an overall functional disability score. | \|  \| **FAP** \| **Control** \| \| --- \| --- \| --- \| \| Somatic Symptoms \| 30.8 (16.0) \| 8.2 (7.0 \| \| Functional Disability \| 11.3 (7.4) \| 2.3 (5.1) \| \|  \|  \|  \| \| P-60 Temperature \| 46.8 (2.0) \| 46.8 (1.6) \| \| Preconditioning mean \| 58.2 (17.1) \| 59.8 (13.3) \| \| Preconditioning 10s \| 61.6 (16.7) \| 64.2 (14.4) \| \| Preconditioning 20s \| 57.9 (19.5) \| 58.7 (15.3) \| \| Preconditioning 30s \| 54.5 (23.9) \| 56.2 (17.7) \| \|  \|  \|  \| \| Water bath 0s \| 32.6 (24.1) \| 31.4 (22.5) \| \| Water bath 10s \| 37.2 (26.5) \| 35.2 (25.3) \| \| Water bath 20s \| 39.7 (25.9) \| 31.1 (23.0) \| \|  \|  \|  \| \| Conditioning 40s \| 60.9 (18.7) \| 55.5 (18.8) \| \| Conditioning 50s \| 55.9 (17.7) \| 46.3 (20.3) \| \| Conditioning 60s \| 51.1 (22.9) \| 39.9 (20.0) \|   Significant within-individual decrease across the 3 preconditioning ratings  (*b* = -4.0, SE = 0.96, *p* < 0.001) but no significant variation between FAP and healthy groups (*b* = .64, SE = 1.44, *p* = .659).  Older youth reported significantly higher preconditioning pain ratings than younger youth (*b* = 2.54, SE = 0.95, *p* = .009).  FAP youth reported significantly higher pain ratings in the second (*t* = 2.84, *p* < .01) and third (t = 2.96, *p* < .01) CPM ratings than healthy controls.  FAP youth reported significantly higher third water bath pain ratings  (*t* = 2.02, *p* < .05) than healthy controls.  FAP group had significantly higher mean conditioning thermode pain ratings after controlling for mean preconditioning thermode pain ratings in comparison to that of healthy controls. (F(1,129) = 16.1, *p <*  .001, n^2^ = .11).  Significant FAP X trial interaction (*b* = 3.69, SE = 1.08, *p* = .001). FAP youth pain ratings declined slower (b = -4.19, SE = 1.15, p < .001) than healthy controls (b = -7.79, SE = .83, p < .001).  Pain inhibition and pain facilitation were differentially distributed between FAP and healthy youth (χ^2^ = 8.56, *p* = .003)  Significant race X trial interaction (b = 2.30, SE = 1.10, p = .037) with weaker CPM effects in Caucasian than Non-Caucasian youth.  FAP group reported significantly higher levels of somatic symptoms (30.8 ± 16.0) and functional disability (11.3 ± 7.4) than healthy controls (8.2 ± 7.0; 2.3 ± 5.1).  Significant CSI x trial interaction (*b* = 1.30, SE = .53, *p* = .014)  Significant functional disability x trial interaction (*b* = 1.40, SE = .53, *p* = .009) |
| **Morris, Bruehl et al. (2021)** USA | **Adolescents with functional abdominal pain** 183 adolescents ages 11 to 17 years old (mean = 14.6, SD = 1.9) enrolled in RCT evaluate efficacy 8-week online CBT intervention to online education for chronic abdominal pain (90 CBT group, 93 online education). Met Rome IV criteria for pediatric pain-related functional gastrointestinal disorder.  Assigned at baseline to one of three FAP patient subgroups (High Pain Dysfunctional HPD, High Pain Adaptive HPA, Low Pain Adaptive LPA)  Demographics for each group outlined below.  Age   \| Age  years \| M \| SD \| \| --- \| --- \| --- \| \| HPD \| 15.0 \| 1.8 \| \| HPA \| 14.5 \| 1.9 \| \| LPA \| 14.1 \| 1.8 \|   Sex male (female)  HPD 16 (54)  HPA 24 (55)  LPA 24 (10) | Analgesia free 4 hours prior to testing  **Test Stimulus: Heat pain intensity**  Delivered by a thermode (30 × 30 mm) applied to the ventral forearm of the participant's non-dominant arm and administered via a computerized Medoc TSA-II Neurosensory Analyzer. The thermode temperature eliciting a pain rating between 50 and 70 was determined first (P-60) applied in sequences of 15 second pulses at 45 °C, 46 °C, and 47 °C, and at additional lower or higher temperatures as warranted until the P-60 was identified.  **Conditioning Stimulus: Hot water immersion**  Dominant hand submerged in a water bath with a 46.5°C temperature.  **Procedure**  Perceived pain intensity was rated by participants on a 0 to 100 scale. First P-60 temperature determined for test stimulus. Then, the thermode was moved to a non-overlapping location on the non-dominant ventral forearm, and the pre-conditioning test period began. The forearm thermal test stimulus was applied at the P-60 temperature continuously for a 30-second period, with three pre-conditioning pain ratings obtained at 10-second intervals. Next, participants took a 10-minute brea, following which they immersed their dominant hand in the hot water bath for 60 seconds. During this, participants provided three water bath pain ratings at 0, 10 and 20 seconds. At 30 seconds, the forearm thermal test stimulus was again applied at the P-60 temperature continuously for a 30-second period, with three conditioning pain ratings obtained at 40, 50 and 60 seconds. | **QST**  The Medoc TSA-II Neurosensory analyzer and 30 × 30mm thermode was used to determine pain threshold, pain tolerance, and TSSP.  **Pain threshold** - thermode attached to the ventral forearm of the participant’s non-dominant arm and moved upwards on the forearm to a new location for each trial. From a baseline of 32°C, probe temperature increased at a rate of 0.5°C/sec until the participant responded by pressing a button to indicate either first felt pain. Four trials performed.  **Pain tolerance** same principle as above but protocol started at an adaptation temperature of 40°C and the temperature increased at a ramp rate of 0.5°C per second. Participants were instructed to terminate the stimulus by clicking on a computer mouse “when you can’t stand the heat pain any longer.” Temperature maximum limit was 51°C. Four pain tolerance trials were conducted, with a 25 second interstimulus interval during which the thermode was moved to a new, non-overlapping location.  **Temporal summation of second pain (TSSP)** A standardized oscillating thermal stimulation protocol. A sequence of 10 heat pulses, 0.5 seconds in duration, with a 40°C adaptation temperature and 47°C target stimulus intensity was applied to the ventral forearm. These heat pulses were delivered at a frequency of 0.4 Hz. Participants rate the intensity of pain sensations immediately after the peak of each pulse using a verbal numeric rating scale of 0 (“no pain”) to 100 (“most pain possible”). Experimenters terminated the temporal summation of second pain protocol immediately following any pain ratings of 100. Temporal summation of second pain was computed as the difference between the maximum pain rating and the first pulse pain rating.  **Pain interference**  8-item PROMIS Pediatric Pain Interference - Short Form. assessed at baseline (pre-treatment) and at 1- (mid-treatment), 2- (post-treatment), 6-, and 12-month follow-up assessments.    **Abdominal pain**  Four-item API (Abdominal Pain Index). assessed at baseline (pre-treatment) and at 1- (mid-treatment), 2- (post-treatment), 6-, and 12-month follow-up assessments.  **Gastrointestinal symptoms**  7-item gastrointestinal subscale of the CSSI-24 (Children’s Somatic Symptoms Inventory–24). assessed at baseline (pre-treatment) and at 1- (mid-treatment), 2- (post-treatment), 6-, and 12-month follow-up assessments.    **Baseline scores (to allocate grouping)** Abdominal Pain Index (as above)  Children’s Somatic Symptoms Inventory–24 (CSSI-24) - gastrointestinal and non-gastrointestinal symptom subscales.  Pain Response Inventory – catastrophizing subscale.  Children’s Depression Inventory. 27-item questionnaire,.  Functional Disability Inventory. 15-items assessing psychosocial and physical functioning rated on a 5-point scale (0 = ‘no trouble’, 4 = ‘impossible’). Sum of all items is used as an overall FDI score.  Pain Beliefs Questionnaire-Short Form pain threat, problem-focused coping efficacy, and emotion-focused coping efficacy subscales. | **HPD group**   \|  \| Mean \| SD \| \| --- \| --- \| --- \| \| Pain threshold \| 43.0 \| 3.7 \| \| Pain tolerance \| 46.9 \| 2.0 \| \| TSSP (max change) \| 15.3 \| 18.7 \| \| CPM (difference) \| 6.1 \| 15.5 \| \| Abdominal pain \|  \|  \| \| - baseline \| 2.8 \| 0.8 \| \| - mid treatment \| 2.3 \| 0.9 \| \| - post treatment \| 1.9 \| 1.0 \| \| - 6m follow up \| 1.7 \| 1.1 \| \| - 12m follow up \| 1.6 \| 1.1 \| \| Gastrointestinal symptoms \|  \|  \| \| - baseline \| 2.1 \| 0.7 \| \| - mid treatment \| 1.7 \| 0.8 \| \| - post treatment \| 1.4 \| 0.8 \| \| - 6m follow up \| 1.4 \| 0.9 \| \| - 12m follow up \| 1.4 \| 0.9 \| \| Pain interference \|  \|  \| \| - baseline \| 15.9 \| 6.6 \| \| - mid treatment \| 15.8 \| 7.3 \| \| - post treatment \| 12.4 \| 7.6 \| \| - 6m follow up \| 12.2 \| 8.4 \| \| - 12m follow up \| 11.1 \| 8.2 \|   **HPA group**   \|  \| Mean \| SD \| \| --- \| --- \| --- \| \| Pain threshold \| 42.6 \| 33.1 \| \| Pain tolerance \| 46.7 \| 1.7 \| \| TSSP (max change) \| 18.3 \| 17.1 \| \| CPM (difference) \| 2.7 \| 19.3 \| \| Abdominal pain \|  \|  \| \| - baseline \| 2.2 \| 0.8 \| \| - mid treatment \| 1.8 \| 0.9 \| \| - post treatment \| 1.4 \| 0.9 \| \| - 6m follow up \| 1.4 \| 0.8 \| \| - 12m follow up \| 1.4 \| 0.9 \| \| Gastrointestinal symptoms \|  \|  \| \| - baseline \| 1.5 \| 0.7 \| \| - mid treatment \| 1.1 \| 0.7 \| \| - post treatment \| 1.0 \| 0.6 \| \| - 6m follow up \| 0.9 \| 0.6 \| \| - 12m follow up \| 1.0 \| 0.7 \| \| Pain interference \|  \|  \| \| - baseline \| 10.4 \| 5.8 \| \| - mid treatment \| 9.5 \| 6.6 \| \| - post treatment \| 8.0 \| 6.5 \| \| - 6m follow up \| 7.4 \| 6.1 \| \| - 12m follow up \| 8.0 \| 7.3 \|   **LPA group**   \|  \| Mean \| SD \| \| --- \| --- \| --- \| \| Pain threshold \| 42.9 \| 3.4 \| \| Pain tolerance \| 47.2 \| 1.5 \| \| TSSP (max change) \| 16.4 \| 16.3 \| \| CPM (difference) \| 3.9 \| 15.8 \| \| Abdominal pain \|  \|  \| \| - baseline \| 1.3 \| 0.6 \| \| - mid treatment \| 1.0 \| 0.8 \| \| - post treatment \| 0.8 \| 0.7 \| \| - 6m follow up \| 0.9 \| 0.9 \| \| - 12m follow up \| 0.8 \| 0.7 \| \| Gastrointestinal symptoms \|  \|  \| \| - baseline \| 0.9 \| 0.6 \| \| - mid treatment \| 0.6 \| 0.5 \| \| - post treatment \| 0.6 \| 0.4 \| \| - 6m follow up \| 0.6 \| 0.6 \| \| - 12m follow up \| 0.7 \| 0.6 \| \| Pain interference \|  \|  \| \| - baseline \| 3.9 \| 3.6 \| \| - mid treatment \| 5.2 \| 6.2 \| \| - post treatment \| 4.3 \| 4.2 \| \| - 6m follow up \| 5.3 \| 6.4 \| \| - 12m follow up \| 2.7 \| 3.9 \|   CPM was not associated with pain-related interference at baseline (b = .393, SE = .398, *p* = .325), although did significantly predict changes in pain-related interference over time (b = −.108, SE = .039, *p* = .006).  Beyond any effects of the intervention, pain-related interference declined significantly over time for participants with stronger baseline CPM (b = −.858, SE = .396, *p* = .032), but not those with weaker baseline CPM (b = −.642, SE = .400, *p*= .110).  Neither pain threshold (b = .042, SE = .042, *p* = .320), pain tolerance (b = .024, SE = .049, *p* = .633), nor temporal summation of second pain (b = −.032, SE = .041, *p* = .428) predicted changes in pain­ related interference. |
| **Nahman-Averbuch, Leon et al. (2019)** USA | **Migraine Group (n = 19)**  Aged 14.3 ± 1.9  5 Male, 14 Female  Migraine duration (y) 3.4 ± 3.1  Migraine frequency (days per month) 12.1 ± 9.1  Mean pain of migraine attack 4.6 ± 2.3  PedMIDAS 69.7 ±79.9  **Family History of Migraine (Fam-His) Group (n = 19)**  Aged 14.4 ± 1.8  7 Male, 12 Female  **Healthy Group (n = 28)**  14.3 ± 1.7  7 Male, 12 Female | **Test Stimulus (TS) 1: Tonic Heat**  Delivered using 16x16mm thermode (Medoc, Israel) applied to the lower dominant leg for 30s at 46.0°C. Baseline temperature of 32°C, increased/decreased at 5°C/s.  **Test Stimulus (TS) 2: Pressure Pain**  Delivered using a 1cm diameter probe pressure algometer (Medoc, Israel) applied to the lower dominant leg. Increase rate of 60 kPa. Pressure pain threshold was determined as the force at which participant felt a sensation of pain.  **Conditioning Stimulus: Cold Pressor Test**  Immersion of the nondominant foot in a cold-water bath maintained at 8°C for 60s. Participants’ pain ratings were measures using mechanical visual analogue scale.  **Procedure**  Two baseline paradigms of heat or pressure stimulation were delivered in a random order to the participant. Following this, the two conditioned paradigms (conditioning stimulus together with the test stimulus) were delivered in the same order as the baseline paradigms. Specifically, test stimuli were delivered three times during the last 30 seconds of the cold pressor test. Paradigms were separated with 8-minute breaks. | **Pain Intensity: Visual Analogue scale (computerised)**  Participants rated pain via a digitally presented visual analogue scale ranging from ‘no pain sensation’ to ‘most intense pain imaginable’.  **Migraine Health History Form:**  3 parts: parent reported child general health and demographics, Child reported migraine characteristics, child reported medications.  **Functional Disability: Functional Disability Index (FDI)**  15-items assessing psychosocial and physical functioning rated on a 5-point scale (0 = ‘no trouble’, 4 = ‘impossible’).  **Paediatric Migraine Disability Assessment Scale (PedMIDAS):**  6-item self-report questionnaire assessing number of days of migraine presence.  **The Patient-Reported Outcomes Measurement Information System (PROMIS):**  32 item self-report questionnaire assessing fear (8-items), anxious misery (8-items), hyperarousal (8-items) and depressive symptoms (8-items). | **Familiarisation stimulus response curve:**  Significant effects of temperature (*F*(1,459) = 564.3, *p* < .0001), group (*F*(2, 459) = 3.6, *p* = .027), and group x temperature interaction (*F*(2, 459) = 5.5, *p* = .004) in the stimulus response curve.  Fam-His group gave higher pain rating than the migraine group, whose rating were in turn greater than those by the healthy group. (no descriptive statistics provided)  Significant main effect of temperature on pain ratings (*F*(1, 459) = 426.6, *p* < .0001), with pain ratings increasing with temperature.  **Heat CPM:**   \|  \| **Migraine** \| **Fam-His** \| **Control** \| \| --- \| --- \| --- \| --- \| \| **Baseline TS** \|  \|  \|  \| \| Repetition 0 \| 3.35 ± 1.84 \| 3.51 ± 1.59 \| 3.08 ± 1.96 \| \| Repetition 1 \| 4.23 ± 2.42 \| 4.26 ± 1.68 \| 9.36 ± 2.25 \| \| Repetition 2 \| 4.49 ± 3.02 \| 4.24 ± 1.97 \| 3.94 ± 2.21 \| \| Repetition 3 \| 4.53 ± 3.15 \| 3.90 ± 1.81 \| 3.82 ± 2.38 \| \|  \|  \|  \|  \| \| **Conditioning TS** \|  \|  \|  \| \| Repetition 0 \| 3.71 ±2.55 \| 3.14 ± 1.79 \| 3.48 ± 2.30 \| \| Repetition 1 \| 3.13 ± 2.60 \| 2.36 ± 1.74 \| 2.71 ± 2.30 \| \| Repetition 2 \| 3.58 ± 2.84 \| 3.22 ± 1.83 \| 3.16 ± 2.32 \| \| Repetition 3 \| 3.60 ± 2.88 \| 3.10 ± 2.14 \| 3.47 ± 1.97 \| \|  \|  \|  \|  \| \| **CS Pain ratings** \|  \|  \|  \| \| Repetition 1 \| 4.3 ± 2.8 \| 4.1 ± 2.6 \| 2.3 ± 1.8 \| \| Repetition 2 \| 404 ± 2.7 \| 4.9 ± 2.8 \| 3.2 ± 2.2 \| \| Repetition 3 \| 4.2 ±2.9 \| 3.9 ± 2.8 \| 3.2 ± 2.2 \| \|  \|  \|  \|  \| \| **CPM Response** \|  \|  \|  \| \| Repetition 1 \| -1.10 ± 1.76 \| -1.84 ± 1.88 \| -1.38 ± 1.31 \| \| Repetition 2 \| -0.65 ± 2.90 \| -0.97 ± 1.86 \| -0.95 ± 1.71 \| \| Repetition 3 \| -0.66 ± 2.38 \| -0.70 ± 1.98 \| -0.50 ± 1.35 \|   Significant paradigm effect (*F*(1, 50) = 64.06, *p* < .0001); baseline pain intensity ratings were higher compared to pain intensity ratings during the conditioned paradigm.  Significant paradigm X repetition interaction (*F*(2, 49) = 3.21, *p* = .044).  Significant effect of order (*F*(1, 49) = 4.81, *p* = .033); participants who received the baseline heat paradigm first had overall lower heat pain intensity ratings compared to participants who received the baseline pressure paradigm first.  Significant effect of repetition (*F*(2, 100) = 8.87, *p* < .001), with post-hoc Tukey analyses revealing that the first CPM response was larger compared to the second and third responses (Tukey-adjusted *p* < 0.05)  Conditioning pain intensity significantly differed across repetitions (*F*(2, 120) = 7.04, *p* = .001). Post hoc analysis showed second rating was significantly higher than the first and third pain ratings (*p* < .05).  Significant group X repetition (*F*(4, 120) = 4.36, *p* = .003). No significant variation in migraine group  Fam-His group showed greater ratings in the second repetition than the first and third (*p* < .05) Healthy group showed lowest first rating compared to second and third (*p* < .05)  **Pressure CPM:**   \|  \| **Migraine** \| **Fam-His** \| **Control** \| \| --- \| --- \| --- \| --- \| \| **Baseline TS** \| \|  \|  \| \| Repetition 0 \| 396.9 ± 199.8 \| 408.2 ± 172.4 \| 402.3 ± 170.0 \| \| Repetition 1 \| 347.0 ± 141.8 \| 395.3 ± 186.2 \| 367.8 ± 172.4 \| \| Repetition 2 \| 310.4 ± 138.7 \| 383.9 ± 173.9 \| 356.6 ± 193.8 \| \| Repetition 3 \| 325.0 ± 147.4 \| 390.0 ± 160.1 \| 353.8 ± 201.1 \| \|  \|  \|  \|  \| \| **Conditioning TS** \| \|  \|  \| \| Repetition 0 \| 319.2 ± 130.3 \| 355.6 ± 156.8 \| 316.6 ± 158.6 \| \| Repetition 1 \| 346.3 ± 171.8 \| 416.5 ± 215.6 \| 342.3 ± 171.8 \| \| Repetition 2 \| 344.3 ± 190.1 \| 400.1 ± 158.3 \| 348.0 ± 155.9 \| \| Repetition 3 \| 328.8 ± 165.2 \| 393.6 ± 192.5 \| 318.8 ± 107.5 \| \|  \|  \|  \|  \| \| **CS Pain ratings** \| \|  \|  \| \| Repetition 1 \| 3.6 ± 2.8 \| 4.1 ± 2.7 \| 2.9 ± 2.1 \| \| Repetition 2 \| 4.1 ± 3.0 \| 4.4 ± 2.7 \| 3.3 ± 2.2 \| \| Repetition 3 \| 3.8 ± 3.0 \| 3.7 ± 2.7 \| 3.4 ± 2.5 \| \|  \|  \|  \|  \| \| **CPM Response** \| \|  \|  \| \| Repetition 1 \| 0.7 ± 126.0 \| -21.2 ± 142.7 \| 8.8 ± 111.9 \| \| Repetition 2 \| -33.9 ± 85.8 \| -16.2 ± 122.6 \| -16.3 ± 115.7 \| \| Repetition 3 \| -3.8 ± 77.6 \| -3.5 ±135.8 \| 6.5 ± 85.0 \|   Significant order effect (*F*(1, 57) = 4.24, *p* = .044) |
| **Nahman-Averbuch, Schneider et al. (2021)**  USA | **Adolescents with migraine** N=20 (4 male, 16 female)  **Ethnicity**  Caucasian 16  African American 4  **Age**  Mean age: 14.8 ± 2.2 years old (range 10-17)  **Headache occurrence pre inclusion**  Had headaches for 56.2±37.1 months prior to enrolment, with an average duration of untreated headaches of 7.4±6.9 hours.  Participants completed eight weekly CBT sessions. | **Test Stimulus: Pressure pain**  Pressure pain thresholds (PPT) delivered with a pressure algometer (1cm diameter probe) to the lower dominant leg (anterior tibialis muscle) or the trapezius in random order. The increase rate was 60 kPa/second. Pressure was gradually increased until the participant felt a sensation of pain which they indicated via button press. The PPT value was an average of two PPT trials.  **Conditioning Stimulus: Cold pressor test**  Immersion of the non-dominant foot into a cold-water bath (8°C) for 60 seconds. Participants rated pain intensity from this stimulus after 20 seconds of immersion using a separate mechanical VAS.  **Procedure**  The test stimulus was delivered alone and then, after an 8-minute break, delivered concurrently during the last 30 seconds of the conditioning stimulus. The two test stimuli were delivered first in random order with a 2-minute break between them. | Continuous variable of change in absolute headache days from 28-day baseline to the last 28 days of the study  Change in headache frequency was determined by examining the rate of the absolute number of headache days, per 28-day period, at baseline and during the last 28 days of the study (the last four weeks of CBT sessions).  Percent headache reduction, which was the percent change in headache between baseline and during the last 28 days of the study  Pain intensity and unpleasantness were defined using a radio analogy along a VAS.  Neuroimaging – MRI – (A T1 anatomical scan was always completed first following two resting-state pseudo- continuous arterial spin labelling (pCASL), and two resting-state blood oxygen level- dependent (BOLD) scans in a random order. | **CPM before CBT intervention**   \|  \| M \| SD \| \| --- \| --- \| --- \| \| Trapezius \|  \|  \| \| -Baseline PPT (kPa) \| 142.7 \| 77.2 \| \| -CPM PPT (kPa) \| 161.5 \| 82.5 \| \| -CPM response \| -18.9 \| 35.7 \| \| Leg \|  \|  \| \| -Baseline PPT (kPa) \| 280.6 \| 124.4 \| \| -CPM PPT (kPa) \| 324.4 \| 136.8 \| \| -CPM response \| -43.8 \| 78.3 \|   **Other**  The average pain intensity and unpleasantness ratings during the MRI scans were 1.2±1.8 and 1.2±1.9, respectively.  **Headache frequency**  Before CBT : 14.6 ± 7.4 days per 28-day assessment (range 8-28)  After CBT : 9.9 ± 7.2 days per 28-day assessment (range 2-28)  **CPM at baseline**  A significant CPM response was shown before CBT. During the conditioning stimulus, a significant increase in PPT at the trapezius (t(19)=−2.36, *p* = .029) and leg (t(19)=−2.50, *p* = .022) was found, although the response varied across participants. Baseline CPM responses were related to headache frequency reduction after CBT. Lower CPM response at the trapezius was related with greater reduction in headache days (*r* = .492, *p* = .028). No correlation was found between CPM responses at the leg and headache frequency reduction (*r* = .240, *p* = .309). |
| **Nahman-Averbuch, Thomas, et al. (2021)**  **USA** | **Adolescents with** **migraine**  N = 19 adolescents with migraine (14.9 ± 2.3 years of age, mean ± SD; 16 female),   \|  \| M \| SD \| \| --- \| --- \| --- \| \| Headache frequency per month \| 13.9 \| 7.0 \| \| Headache attack duration (hours) \| 9.8 \| 11.6 \| \| PedMIDAS \| 46.5 \| 26.6 \| \| Days of school missed due to headache in last semester \| 4.0 \| 3.3 \|   **Healthy adolescents**  N = 20 (13.8 ± 2.5 years of age, mean ± SD; 16 female) | **Test stimulus: Pressure pain**  Pressure pain thresholds (PPT) delivered with a pressure algometer (1cm diameter probe) to the trapezius. The increase rate was 60 kPa/second. Pressure was gradually increased until the participant felt a sensation of pain which they indicated via button press.  **Conditioning stimulus: Cold pressor test**  Immersion of the non-dominant foot into a cold-water bath (8°C) for up to 60 seconds. Participants rated pain intensity from this stimulus after 20 seconds of immersion using a separate mechanical VAS.  **Procedure**  The test stimulus was delivered alone and then delivered concurrently during the last 30 seconds of the conditioning stimulus. | **Pain unpleasantness rating** Participants indicated the magnitude of pain sensation on a 0 (no pain/unpleasantness) to 10 (the most intense pain/ unpleasantness imaginable) VAS.  **Pediatric Migraine Disability Assessment Scale** Used to assess migraine disability. | **Adolescents with migraine** PedMIDAS score: 46.5 ± 26.6   \|  \| M \| SD \| \| --- \| --- \| --- \| \| Cold pain intensity \| 3.8 \| 2.4 \| \| Cold pain unpleasantness \| 5.0 \| 2.5 \| \| PPT baseline \| 146 \| 79.1 \| \| CPM response \| -20.4 \| 35.1 \|   **Healthy adolescents**   \|  \| M \| SD \| \| --- \| --- \| --- \| \| Cold pain intensity \| 3.1 \| 2.1 \| \| Cold pain unpleasantness \| 4.4 \| 2.8 \| \| PPT baseline \| 248 \| 145.5 \| \| CPM response \| -3.4 \| 72.2 \|   No differences were found between adolescents with migraine and healthy controls for ratings of cold pain intensity (estimate = −0.78, 95% CI: −2.22, 0.66, *p* = .290) nor cold pain unpleasantness (estimate = −0.91, 95% CI: −2.55, 0.72, *p* = .274)  PPT prior to immersion (PPT_baseline) were significantly lower in adolescents with migraine (146.0 ± than healthy controls (estimate = 124.28, 95% CI: 58.98, 189.59, *p* < 0.001; effect size, *d* = 1.40) Sex was a significant confounder for PPT_baseline.  Considering CPM responses, no group difference was found between adolescents with migraine and healthy controls (estimate = 17.57, 95% CI: −21.30, 56.44, *p* = .376). |
| **Ocay, Larche et al. (2022)** Canada | **Adolescents with primary or secondary musculoskeletal (MSK) pain and healthy controls**   \| Age (years) \| M \| SD \| \| --- \| --- \| --- \| \| MSK \| 14.93 \| 1.95 \| \| Control \| 14.99 \| 1.96 \|  \| Sex \| Female \| Male \| \| --- \| --- \| --- \| \| MSK \| 247 \| 55 \| \| Control \| 32 \| 48 \|  \| Race \| Caucasian \| Person of colour \| \| --- \| --- \| --- \| \| MSK \| 231 \| 70 \| \| Control \| 58 \| 22 \|  \| Past hospitalisation in last 48 hours \| Yes \| No \| \| --- \| --- \| --- \| \| MSK \| 90 \| 212 \| \| Control \| 14 \| 66 \|  \| Previous surgery \| No \| Yes \| \| --- \| --- \| --- \| \| MSK \| 182 \| 120 \| \| Control \| 55 \| 25 \|   **MSK patient details**  Primary location of pain: 11 head and neck; 24 upper limbs; 4 thorax; 175 back; 88 lower limbs  Pain radiated for 48%  Presence of a secondary pain site 52%  Mild-moderate pain intensity (NRS 3.34±2.41) was reported by the patients the day of the assessment  Pain rating over last month: average pain (5.81±1.93); worst pain (8.39±1.56); best pain (1.87±1.86)  Time period of having pain: 223 pain for more than 12 months; 29 pain for 3–6 months; 50 pain for between 6 and 12 months.    Frequency of pain: 232 pain at least once a day; 50 pain every second day; 20 pain only once a week.  Duration: 180 painful episode to be constant; 5 painful episodes lasted a few seconds; 42 painful episodes lasted a few minutes; 75 painful episodes lasted a few hours | **Test stimulus: Heat pain**  Assessed via thermode applied to the right volar forearm to reach a predetermined test temperature to a pain intensity 50/100 (T50). Once the target temperature was reached, it remained constant for 120 seconds. Baseline temperature of 32°C which increased 0.3°C/second. Participants evaluated their pain with a computerized visual analogue scale (CoVAS) ranging from 0 to 100. Average pain intensity was calculated.  **Conditioning Stimulus: Cold pressor test**  Immersion of their left forearm cold water (12°C) for 120 seconds. Every 15 seconds participants to rated their pain intensity using a NRS 0–10. Average pain intensity during the conditioning stimulus was then calculated.    **Procedure**  Heat pain intensity was assessed before and immediately after the conditioning stimulus. | **Pain assessment**  Face-to-face interview. Duration and frequency of pain assessed using NRS. Douleur Neuropathique 4 (DN4) was administered to assesses potential neuropathic aspects of pain.  **Pain catastrophising**  Pain Catastrophizing Scale for Children (PCS-C)  **Disability**  Functional Disability Inventory (FDI)  **Anxiety and depression**  Revised Child Anxiety and Depression Scale (RCADS) questionnaire  **Sleep quality**  Pittsburgh Sleep Quality Index (PSQI) questionnaire  **Quantitative Sensory Testing**  For adolescents with MSK pain, mechanical QST was performed on the left volar forearm and the painful anatomical region. For healthy participants mechanical QST was performed on the left volar forearm. Thermal QST was performed on the left volar forearm for all participants  *Mechanical detection threshold (MDT).* Assessed via calibrated von Frey filaments ranging between 0.008 and 300 grams.  *Dynamic mechanical allodynia (DMA).* A standardized brush exerting light touch at a single stroke for 2 cm in length applied five times.  *Vibration detection threshold (VDT).* A tuning fork applied to a joint or bony prominence of the tested area three times  *Mechanical pain summation (MPS).* One and 10 stimulations from a calibrated pinprick applied, with participants reported their pain immediately at the end of the stimulation(s) and every 15 seconds post-stimuli during a 60-second period.  *Wind-up ratio (WUR)*. Ratio of the average pain intensity immediately reported after the train of 10 stimuli over the average pain intensity immediately reported after one stimulus.  *Pressure pain threshold (PPT).* Assessed via handheld algometer  *Warm detection threshold (WDT), and heat*  *pain threshold (HPT).* A 9-cm^2^ warm thermode (Q-sense) a with a baseline 32°C, 0.3°C/second upslope was applied three times. | **MSK group**   \|  \| M \| SD \| \| --- \| --- \| --- \| \| Adolescent Pediatric Pain Tool \|  \|  \| \| Pain locations, x/67 \| 8.78 \| 8.4 \| \| Sensory descriptors, x/37% \| 23.26 \| 15.36 \| \| Affective descriptors, x/11% \| 15.57 \| 17.41 \| \| Evaluative descriptors, x/8% \| 43.73 \| 24.52 \| \| Temporal descriptors, x/24% \| 29.01 \| 15.96 \| \| DN4 score \| 2.96 \| 2.03 \| \| FDI total score \| 15.79 \| 9.76 \| \| PCS total score \| 28.4 \| 9.98 \| \| RCADS total T-score \| 52.27 \| 14.08 \| \| PSQI total score \| 7.81 \| 3.77 \| \| MDTlog (mN) control area \| 0.69 \| 1.22 \| \| MDTlog (mN) test area \| 0.67 \| 1.77 \| \| DMAlog (NRS 0–10) control area \| -4.27 \| 1.18 \| \| DMAlog (NRS 0–10) test area \| -3.4 \| 2.07 \| \| VDT (x/8) control area \| 6.72 \| 0.98 \| \| VDT (x/8) test area \| 5.96 \| 1.35 \| \| WURlog (ratio) control area \| 0.75 \| 0.98 \| \| WURlog (ratio) test area \| 0.66 \| 1.03 \| \| PPTlog (kPa) control area \| 5.11 \| 0.47 \| \| PPTlog (kPa) test area \| 5.11 \| 0.65 \| \| WDTlog (°C from baseline) control area \| 0.43 \| 0.69 \| \| HPT (°C) control area \| 39.35 \| 2.73 \| \| CPM efficiency (%) \| -22.16 \| 44.28 \| \| TSP (NRS −10-+10) \| 0.02 \| 2.27 \|   **Healthy control group**   \|  \| M \| SD \| \| --- \| --- \| --- \| \| PCS total score \| 18.55 \| 8.77 \| \| RCADS total T-score \| 46.35 \| 11.59 \| \| PSQI total score \| 4.88 \| 2.61 \| \| MDTlog (mN) control area \| 0.42 \| 1.05 \| \| DMAlog (NRS 0–10) control area \| -4.53 \| 0.48 \| \| VDT (x/8) control area \| 7.04 \| 0.85 \| \| WURlog (ratio) control area \| 0.60 \| 0.54 \| \| PPTlog (kPa) control area \| 5.38 \| 0.54 \| \| WDTlog (°C from baseline) control area \| 0.48 \| 0.66 \| \| HPT (°C) control area \| 39.02 \| 2.6 \| \| CPM efficiency (%) \| -33.37 \| 33.28 \| \| TSP (NRS −10-+10) \| 0.33 \| 2.05 \|   MSK patients showed significantly less efficient CPM than healthy controls (*p* = .014), along with significantly lower vibration detection threshold (*p* = .004 control area, *p* < .001 test area) and lower pressure pain thresholds (*p* < .001 control area, *p* = .024 test area)  The results showed four distinct pain modulatory profiles within patients, including: patients with optimal CPM efficiency and absence of temporal summation (i.e., functional central processing; n = 112); patients showing temporal summation of pain only (i.e., facilitation; n = 18); patients showing suboptimal or inefficient CPM only (i.e., dysfunctional inhibition; n = 136); patients displaying suboptimal or inefficient CPM and the presence of temporal summation of pain (ie, dysfunctional central processing; n = 27). Demographic characteristics and pain intensity did not vary across these profiles. |
| **Ocay, Ye et al. (2022)** Canada | Patients were regrouped from multiple former studies  **Patient Group**  608 patients with persistent pain  Age: 15.18 ± 2.14  Female: 80.92%  Male: 19.08%  Most experienced persistent pain (*n* = 329), though some experienced recurrent pain (*n* = 223) for more than 6 months (*n* = 568)  Pain primarily located in the back (*n* = 410), other areas included head/neck (*n* = 31), the abdomen (*n* = 24), the groin (*n* = 1), the thorax (*n* = 14), the upper extremities (*n* = 18) and the lower extremities (*n* = 109). 50.99% of patients however reported more than one pain site.  **Control Group**  60 controls  Age: 15.06 ± 2.23  Female: 48.33%  Male: 51.67% | **Test stimulus: Heat pain**  Assessed via 9cm^2^ thermode applied to the right forearm to reach a predetermined test temperature to a pain intensity 50/100 (T50). Once the target temperature was reached, it remained constant for 120 seconds. Baseline temperature of 32°C which increased 0.3°C/second. Participants evaluated their pain with a computerized visual analogue scale (CoVAS) ranging from 0 to 100. Average pain intensity was calculated.  **Conditioning Stimulus: Cold pressor test**  Immersion of their left forearm cold water (12°C) for 120 seconds. Every 15 seconds participants to rated their pain intensity using a NRS 0–10. Average pain intensity during the conditioning stimulus was then calculated.    **Procedure**  Heat pain intensity was assessed before and immediately after the conditioning stimulus. | **Pain intensity:**  Numerical rating scale (NRS), ranging from 0 (no pain) to 10 (worst pain imaginable). Verbally reported during cold pressor test  Computerised visual analogue scale (CoVAS), ranging from 0 (no pain) to 100 (worse pain imaginable).  **Temporal summation of pain (TSP)** The absolute difference in pain intensity of the last 60 seconds of each test stimulus was assessed as the facilitatory pain response. | **Average Heat Pain Threshold:**  Patients: 38.95 ± 3.13 degrees C  Controls: 38.71 ± 2.63 degrees C.  (*F* = 1.17, *p* = 0.28)  **Average Test Temperature:**  Patients: 43.41 ± 2.38 degrees C  Controls: 42.84 ± 2.38 degrees C  (*F*= 4.33, *p* = 0.038, ω^2^ < 0.01)  **CPM Efficiency**  Patients: -26.13% ± 43.20%  Controls: -32.47% ± 35.47%  No significant difference between patients and controls (*F* = 2.21,  *p* = .137)  **Mean Reported Change in Pain Intensity in last 60 secs of TS1** (before conditioning stimulus)  Patients: 0.45 ± 21.70  Controls: 6.46 ± 19.05  Significant difference between patients and controls (*F*= 4.92, *p* = .027, ω^2^ < 0.01)  **Mean Reported Change in Pain Intensity in last 60 secs of TS2** (after the conditioning stimulus)  Patients: 1.84 ± 19.05  Controls: 5.16 ± 14.49  No significant difference between patients and controls (*F* = 1.63, *p* = .202)  **Mean Reported Pain Intensity During Conditioning Stimulus**  Patients: 6.92 ± 2.44  Controls: 6.31 ± 2.41  (*F* = 4.03, *p* = .027, ω^2^ < 0.01).  Three subgroups of patients were identified based on CPM outcomes:   - Cluster 1: best characterized by high pain intensity during the CPT, lack of TSP, and efficient inhibitory CPM. - Cluster 2: best characterized by low pain intensity during the CPT, lack of TSP, and efficient inhibitory CPM. - Cluster 3: best characterized by high pain intensity during the CPT, presence of TSP, and inefficient inhibitory CPM.   Cluster 1: mean age 15.15 (2.12), 218 female and 53 male. Average pain intensity 3.08 (2.78)  Cluster 2: mean age 15.26 (1.86), 156 female and 30 male. Average pain intensity 2.94 (2.50)  Cluster 3: mean age 15.15 (2.48), 118 female and 33 male. Average pain intensity 2.73 (2.48)  The three clusters did not significant different on any demographic variable measured, not pain type, location, presence of secondary pain, or presence of pain before CPM assessment.   \|  \| Cluster 1 (n = 271) \| Cluster 2 (n = 186) \| \| --- \| --- \| --- \| \| Heat pain threshold (°C), mean (SD) \| 38.23 (2.90)b \| 39.98 (3.33)a \| \| Test temperature (°C), mean (SD) \| 42.70 (2.45)b,c \| 44.39 (1.98)a \| \| Change in pain intensity during the last 60 s of TS1 (NRS 2100 to 1100), mean (SD) \| -10.62 (20.17)b, \| 6.89 (17.72)a \| \| Decrease, n (%) \| 73 (26.94) \| 12 (6.45) \| \| Constant, n (%) \| 189 (69.74) \| 139 (74.73) \| \| Increase, n (%) \| 9 (3.32) \| 35 (18.82) \| \| Average pain intensity during CS (NRS 0–10), mean (SD) \| 8.16 (1.47)b \| 4.04 (1.70)a,c \| \| Change in pain intensity during the last 60 s of TS2 (NRS 2100 to 1100), mean (SD) \| -6.11 (16.42)b, \| 2.10 (14.11)a, \| \| Decrease, n (%) \| 39 (14.39) \| 11 (5.91) \| \| Constant, n (%) \| 222 (81.92) \| 157 (84.41) \| \| Increase, n (%) \| 10 (3.69) \| 18 (9.68) \| \| CPM efficiency (%), mean 6 SD \| -41.06 (34.19)c \| -33.10 (37.84) \| \| Inefficient, n (%) \| 45 (16.61) \| 44 (23.66) \| \| Suboptimal, n (%) \| 61 (22.51) \| 44 (23.66) \| \| Optimal, n (%) \| 165 (60.89) \| 98 (52.69) \|  \|  \| Cluster 3 (n = 151) \| Control Subjects  (n = 60) \| \| --- \| --- \| --- \| \| Heat pain threshold (°C), mean (SD) \| 38.96 (2.92)b \| 38.71 (2.63)b \| \| Test temperature (°C), mean (SD) \| 43.49 (2.27)a,b \| 42.84 (2.38)b \| \| Change in pain intensity during the last 60 s of TS1 (NRS 2100 to 1100), mean (SD) \| 12.39 (19.23)a \| 6.46 (19.05)a \| \| Decrease, n (%) \| 6 (3.31) \| 5 (8.33) \| \| Constant, n (%) \| 102 (67.55) \| 43 (71.67) \| \| Increase, n (%) \| 43 (28.48) \| 12 (20.00) \| \| Average pain intensity during CS (NRS 0–10), mean (SD) \| 8.24 (1.32)b \| 6.31 (2.41)a,b,c \| \| Change in pain intensity during the last 60 s of TS2 (NRS 2100 to 1100), mean (SD) \| 15.78 (20.66)a, \| 5.16 (14.49)a,c \| \| Decrease, n (%) \| 7 (4.64) \| 4 (6.67) \| \| Constant, n (%) \| 86 (56.95) \| 47 (78.33) \| \| Increase, n (%) \| 58 (38.41) \| 9 (15.00) \| \| CPM efficiency (%), mean 6 SD \| 9.25 (44.27)a,b \| -32.67 (35.47)c \| \| Inefficient, n (%) \| 97 (64.24) \| 14 (23.33) \| \| Suboptimal, n (%) \| 24 (15.89) \| 12 (20.00) \| \| Optimal, n (%) \| 30 (19.87) \| 34 (56.67) \|   a-c = significant difference at the .05 level though Scheffe´ post-hoc test from Cluster 1 to Cluster 3 respectively. |
| **Ocay, Loewen et al. (2022)** Canada | **Adolescents with chronic back pain**  N = 198; Age mean 15.69 (SD 2.25); Female 162, Male 36  Data below is presented as N  Ethnicity: Caucasian = 179; Black or African American = 10; Asian = 4; Interracial = 5  Duration of pain: 3-6 months = 14; 3-12 months = 41; >12 months = 143  Frequency of pain: Daily = 129; Every second day = 43; Once a week = 26  Duration of painful episodes: Few seconds = 8; Few minutes = 36; 1 hour = 44; Constant = 110  Pathology: Arthritic = 6; Disc protrusion = 8; Mechanical back pain = 14; Scoliosis = 115; Spondylolysis/Spondylolisthesis = 13; Tight hamstrings = 9; Non-specific back pain = 33  Pain location: Neck = 3; Left upper back = 6; Centre upper back = 38; Right upper back = 11; Left middle back = 8; Centre middle back = 37; Right middle back = 12; Left lower back = 12; Centre lower back = 63; Right lower back = 6 | **Test stimulus: Heat pain**  Assessed via 9cm^2^ thermode applied to the right forearm to reach a predetermined test temperature to a pain intensity 5/10. Once the target temperature was reached, it remained constant for 120 seconds. Baseline temperature of 32°C which increased 0.3°C/second. Participants evaluated their pain with a computerized visual analogue scale (CoVAS) ranging from 0 to 100. Average pain intensity was calculated.  **Conditioning Stimulus: Cold pressor test**  Immersion of their left forearm cold water (12°C) for 120 seconds.    **Procedure**  Heat pain intensity was assessed before and immediately after the conditioning stimulus. | **Pain assessment**  Face-to-face interview. Duration and frequency of pain assessed using NRS. Douleur Neuropathique 4 (DN4) was administered to assesses potential neuropathic aspects of pain.  **Pain catastrophising**  Pain Catastrophizing Scale for Children (PCS-C)  **Disability**  Functional Disability Inventory (FDI)  **Anxiety and depression**  Revised Child Anxiety and Depression Scale (RCADS) questionnaire  **Sleep quality**  Pittsburgh Sleep Quality Index (PSQI) questionnaire  **Quantitative Sensory Testing**  Mechanical and thermal QST was performed on the left volar forearm and the most painful region on the back.  *Mechanical detection threshold (MDT).* Assessed via calibrated von Frey filaments ranging between 0.008 and 300 grams.  *Pressure pain threshold (PPT).* Assessed via handheld algometer with a 1cm^2^ probe.  *Heat pain threshold (HPT)and heat pain tolerance (HTT)*  Assessed by a 9cm^2^ thermode (Q-Sense) with a baseline 32°C, 0.3°C/second upslope was applied three times.  *Temporal summation of pain (TSP)*  Assessed via the heat pain trial, whereby participants constantly rated their pain over 120 seconds via CoVAS (as described).  . | \|  \| M \| SD \| \| --- \| --- \| --- \| \| Descriptors of pain used (mean %) \|  \|  \| \| Sensory \| 18.04 \| 11.61 \| \| Affective \| 8.92 \| 12.00 \| \| Evaluative \| 34.13 \| 21.41 \| \| Temporal \| 23.94 \| 13.78 \| \| DN4 score \| 2.46 \| 2.08 \| \| FDI total score \| 15.43 \| 10.31 \| \| RCADS total T-score \| 45.34 \| 12.39 \| \| PSQI total score \| 6.98 \| 3.48 \| \| MDTlog (g) control area \| 0.52 \| 1.65 \| \| MDTlog (g) affected area \| 1.47 \| 12.37 \| \| PPTlog (kPa) control area \| 27.62 \| 14.82 \| \| PPTlog (kPa) affected area \| 26.38 \| 17.44 \| \| HPT (°C) \| 39.24 \| 3.17 \| \| HTT (°C) \| 45.16 \| 2.41 \| \| Test temperature for CPM assessment (°C) \| 43.56 \| 2.51 \| \| CPT average pain score NRS (0–10) \| 6.98 \| 2.32 \| \| CPM efficiency (%) \| -29.44 \| 42.87 \| \| TSP (NRS −10-+10) \| 0.09 \| 2.07 \|   **Other**   \| Average pain reported (NRS 0-10) \| Mean \| CI \| \| --- \| --- \| --- \| \| Neck \| 2.91 \| 2.50-3.32 \| \| Left upper back \| 2.73 \| 2.32-3.14 \| \| Centre upper back \| 3.44 \| 3.00-3.87 \| \| Right upper back \| 2.46 \| 2.05-2.87 \| \| Left middle back \| 2.8 \| 2.39-3.21 \| \| Centre middle back \| 4.32 \| 3.9-4.74 \| \| Right middle back \| 2.56 \| 2.16-3.53 \| \| Left lower back \| 3.07 \| 2.61-3.53 \| \| Centre lower back \| 4.09 \| 3.63-4.54 \| \| Right lower back \| 3.18 \| 2.72-3.64 \|   CPM efficiency was optimal in 51.5% of adolescents, sub- optimal in 22.7% of adolescents and inefficient in 25.8% of adolescents  Adolescents were grouped into three clusters according to principle components analysis; adaptive cluster (C1), pain-sensitive cluster (C2), and high somatic symptom cluster (C3). No significant differences were found between clusters in age, sex, ethnicity, duration of pain, duration of painful episodes, pathology or location of pain. C3 adolescents reported significantly higher pain intensity in all regions of their pain, were more likely to report pain radiating down their legs, and were more likely to be recruited from chronic pain services at the researchers institution. All C3 adolescents reported pain at least every second day.  The C1 cluster (compared to C2 and C3) significantly showed the highest pressure pain threshold in control and affected areas, highest heat pain and tolerance threshold, and lowest pain intensity during the cold pressor task (*p* < .001). This cluster also had a higher proportion of adolescents displaying temporal summation pain (*p* = .005). The C2 cluster in general displayed lower pressure pain threshold in the control and affected area, lower heat pain and tolerance threshold, and higher pain intensity reported during the cold pressor task than C1 (*p* < .001), but also displayed lower scores for all the questionnaires completed than C3 (*p* < .001).   \|  \| **Adaptive**  **cluster (*n* = 89) – C1** \| **Pain-sensitive**  **cluster (*n* = 71) – C2** \| **High somatic symptoms cluster (*n* = 38) – C3** \| \| --- \| --- \| --- \| --- \| \| MDT control area \| 0.42 (0.58) \| 0.75 (2.66) \| 0.31 (0.27) \| \| MDT affected area \| 0.56 (0.95) \| 0.53 (1.14) \| 5.34 (28.04) \| \| PPT control area \| 35.58 (14.05) \| 20.75 (9.53) \| 21.62 (15.68) \| \| PPT affected area \| 35.18 (19.41) \| 19.94 (10.49) \| 17.62 (12.92) \| \| HPT \| 41.04 (2.83) \| 37.74 (2.47) \| 37.802 (2.92) \| \| HTT \| 46.82 (1.30) \| 43.75 (2.13) \| 43.9 (2.48) \| \| CPT average pain score \| 5.68 (2.28) \| 8.08 (1.73) \| -28.53 (34.40) \| \| CPM efficiency \| -38.37 (33.00) \| -18.73 (54.40) \| -25.53 (34.40) \| \| TSP pain score \| 0.68 (1.99) \| -0.69 (2.02) \| 0.17 (1.94) \| |
| **Pas, Rheel et al. (2019)** Belgium | **Functional Abdominal Pain (FAPD) group (n = 39)**  Ages 6-12y  14 Male, 25 Female  36 Anglo-American  3 Non-Anglo-American  14 parental history of chronic pain  15 low parental education  16 Middle parental education  8 High parental education  Median Pain duration = 24 months (IQR = 28)  Median pain episodes/month = 18 (IQR = 20)  **Healthy control group (n = 36)**  Ages 7-11  15 Male, 21 Female  36 Anglo-American  2 parental history of chronic pain  2 parental history of chronic pain  4 low parental education  13 Middle parental education19  19 High parental education | **Test Stimulus: Pressure Pain**  Pressure pain thresholds recorded via algometer with a 1cm^2^ disk applied to trapezial region. Pressure force increased by 1kg/s. Children instructed to say ‘stop’ at onset of pain. Three measurements taken at each testing site with 30-second inter stimulus interval. Average of final 2 values was calculated as final value.  **Conditioning Stimulus: Cold pressor test**  Immersion of nondominant hand in circulating water maintained at 12±1°C in an unclenched, palm up position.  **Procedure**  Participants held their nondominant hand in an unclenched palm-up position, while submerged to 5 cm above the wrist in the tank. After 20 seconds, pressure pain threshold at the trapezial region was reassessed three times, with an interstimulus duration of 30 seconds. | **Pressure Pain at Umbilical and Tibial Regions:**  Pressure pain thresholds recorded via delivery of pressure 1cm^2^ algometer applied to Umbilical (symptomatic) and tibial (asymptomatic) region. Pressure force was increased at a rate of 1kg/s. Children instructed to say ‘stop’ at onset of pain.  3 measurements taken at each testing site with 30-second inter stimulus interval.  Average of final 2 values was calculated as final value.  **Pain Intensity**  Faces Pain Scale—Revised (FPS-R). Scale consisting of 6 faces presented horizontally and relating to a numeric value of 1-10 with endpoints explained as 0 = ‘no pain’ and 10 = ‘worst pain imaginable’.  **Fear of pain**  Pain Related Fear: Dutch Fear of Pain Questionnaire—parent report (FOPQ-P)  **Parental catastrophising**  Parental Pain Catastrophising: Dutch Parent version of the Pain Catastrophizing Scale (PCS-P)  **Functional disability**  Dutch version of the Functional Disability Inventory (FDI) | **CPM Outcomes:**   \| Median (IQR) \| **FAPD** \| **Control** \| \| --- \| --- \| --- \| \| Trapezial PPT \| 1.21 (.79) \| 1.49 (.77) \| \| Tibial PPT \| 2.48 (.98) \| 3.33 (.92) \| \| Umbilical PPT \| 1.04 (.44) \| 1.48 (.44) \| \|  \|  \|  \| \| CPM Trapezius PPT \| 1.37 (.72) \| 1.86 (.97) \| \| Absolute Difference \| -.11 (.46) \| -.36 (.56) \| \| % Change \| -9.04 (36.84) \| -25.20 (36.79) \| \|  \|  \|  \| \| FPS-R After CPT \| 8 (6) \| 4 (4) \|   Pressure pain thresholds at the trapezial (*p* = 0.011), tibial (*p* < 0.001), and umbilical region (*p* < .001) were significantly lower in FAPD group compared to healthy controls.  The FAPD group (*p* = .035) and healthy controls (*p* < .001) both showed a significant increase in PPT during the cold pressor test compared to before the cold pressor test.  FAPD group showed significantly less negative CPM magnitude ( *p* = .003) and CPM percentage change *p* = .023) than healthy controls.  **Additional Outcomes:**  Median (IQR)   \|  \| **FAPD** \| **Control** \| \| --- \| --- \| --- \| \| **Child-Report** \|  \|  \| \| Pain Intensity \| 4 (2) \| 0 (2) \| \| **Parent Report** \|  \|  \| \| Pain Related Fear (*m* (SD)) \| 36.38 (11.62) \| 24.83 (14.44) \| \| School Functioning (*m* (SD)) \| 10.90 (3.96) \| 7 (4.37) \| \|  \|  \|  \| \| FOPQ-P Fear \| 11 (5) \| 8 (8) \| \| FOPQ-P Avoidance \| 15 (5) \| 11 (6) \| \|  \|  \|  \| \| PCS-P Total \| 22 (14) \| 7 (9) \| \| PCS-P Magnification \| 2 (3) \| 1,50 (2) \| \| PCS-P Helplessness \| 9 (7) \| 1 (2) \| \| PCS-P Rumination \| 9 (6) \| 4 (5) \| \|  \|  \|  \| \| Functional Disability \| 7 (15) \| 0 (1) \|   FAPD group reported significantly higher abdominal pain intensity (*p* < .001, *r* = -.68).  FAPD parents reported significantly greater total pain-related fear (*t* = -3.83, *p* < .001, *r* = -.41) and on all three subscales.  School subscale (*t* = -4.18, *p* < .001, *r* = -.42)  Fear subscale (*p* = .003, *r* = -.35)  Avoidance subscale (*p* < .001, *r* = -.46)  FAPD parents catastrophised significantly more than healthy control parents (*p* < .001, *r* = -.57) and across all three subscales.  Helplessness subscale (*p* < .001, *r* = -.29)  Rumination subscale (*p* < .001, *r* = -.69)  Magnification subscale (*p* = .012, *r* = -.44)  FAPD parents reported significantly more disability in their children than parents of healthy children(*p* < .001, *r* = -.72). |
| **Ray & O’Connor (2023)** USA | The 54 participants who took part were healthy young women aged 18-35. This study explored effects of yoga and slow breathing on pain sensitivity and pain modulation.  **Group A (n = 14) – Sitting and normal breathing**  Age 21.29 (2.49)  BMI 21.90 (2.52)  7.1% had chronic pain  Average sleep 7.30 (0.59)  42.9% White/Caucasian  **Group B (n = 11) – Sitting and slow breathing**  Age 20.55 (1.51)  BMI 22.93 (3.49)  0% had chronic pain  Average sleep 7.18 (1.15)  54.5% White/Caucasian  **Group C (n = 15) – Yoga postures and normal breathing**  Age 20.33 (2.02)  BMI 23.07 (4.09)  0% had chronic pain  Average sleep 7.48 (0.88)  66.7% White/Caucasian  **Group D (n = 14) – Yoga postures and slow breathing (Vinyasa)**  Age 20.36 (1.22)  BMI 22.02 (2.83)  0% had chronic pain  Average sleep 7.36 (0.72)  78.6% White/Caucasian | **Test Stimulus: Heat Pain**  Assessed via a 30 × 30 mm thermode attached to the right forearm. The baseline temperature  was 35°C, which increased to target temperatures and returned to baseline at a rate of 8°C per seconds-1. Thirty seconds of 46°C heat was applied, with participants reporting pain intensity every 10 seconds.  **Conditioning Stimulus: Cold pressor test**  Left hand submerged in circulating ice water for 60 seconds  **Procedure**  Heat pain intensity was assessed before and thirty seconds after the left hand was submerged in the ice water. | **State Anxiety**  Assessed using anxiety subscale of Profile of Mood States-II (POMS-II).  **Pain Intensity**  Rated using 0-10 numerical rating scale.  **Exertion**  6-10 Borg Scale was used to gain overall ratings of average physical exertion. | **Overall average ratings of perceived exertion**   \| Group \| N \| Mean \| SD \| SE \| Min \| Max \| \| --- \| --- \| --- \| --- \| --- \| --- \| --- \| \| A \| 8 \| 8 \| 1.852 \| 0.655 \| 6 \| 11 \| \| B \| 9 \| 9.11 \| 2.261 \| 0.754 \| 6 \| 13 \| \| C \| 15 \| 11.6 \| 2.23 \| 0.576 \| 7 \| 16 \| \| D \| 14 \| 12.57 \| 2.821 \| 0.754 \| 8 \| 18 \|   Perceived exertion ratings were significantly higher in the yoga compared to non-yoga conditions (*F* (1, 42) = 23.516, *p* = .001). Heart rates were significantly higher during yoga compared to non-yoga conditions (*F*(1, 48) = 8.550, *p* = .005.) Mean heart rate during yoga were 84 and 95 beats per minute for yoga posture plus normal breathing and the Vinyasa groups respectively.  **Changes in mean pain intensity rating**   \| Group \| Mean \| SD \| \| --- \| --- \| --- \| \| A \| -1.196 \| 1.161 \| \| B \| -0.591 \| 0.882 \| \| C \| -1.3 \| 1.293 \| \| D \| -0.839 \| 1.303 \|   No significant interactions or main effects were found for change in heat pain ratings during CPM.  **Breaths per minutes**  Group A – 16.07  Group B – 12.53  Group C – 18.97  Group D – 15.90  Group B took significantly fewer (*p* = .002) breaths per minute than Group A. Group D took significantly (*p* = .003) fewer breaths compared to Group C and significantly more breaths than Group B (*p* = .002).   \|  \| State anxiety \| \| Systolic blood  pressure (mmHg) \| \| \| --- \| --- \| --- \| --- \| --- \| \| Group \| Pre \| Post \| Pre \| Post \| \| A \| 1.07 (1.69)* \| .57 (.94) \| 106.14 (12.08) \| 100.93 (9.79) \| \| B \| 1.00 (1.61)& \| .64 (1.21) \| 100.18 (6.66) \| 98.09 (97.11) \| \| C \| 2.13 (1.25) \| .20 (.41) \| 100.13 (13.11) \| 96.47 (10.60) \| \| D \| 3.21 (3.04)*& \| .86 (1.66) \| 103.43 (7.07) \| 101.46 (8.40) \|   *& Groups with these symbols were statistically different from one another.  Overall the authors conclude that a single session of low to moderate intensity yoga with or without slow breathing did not change pain sensitivity or endogenous pain modulation, although did induced weak to mild pain, reduce state anxiety and was associated with an expectation of pain. |
| **Schubert-Hjalmarsson, Fasth et al. (2023)** Sweden | **Adolescents with hypermobility spectrum disorder or hypermobile Ehlers‑Danlos syndrome (n = 10)**  Median (range)  Age (years): 15 (13 to 17)  Gender: 4 boys, 6 girls  Beighton score (0-9): 4 (2 to 8)  BMI (kg/m^2^): 19 (17 to 29)  Reported occasions of planned physical activity/week: 0.5 (0 to 3)  Pain intensity: Over the past week: 3.5 (0 to 8); At the time of the assessment: 1.5 (0 to 5)  Pain duration/frequency  No pain: 0  Less than 3 months: 1  1.5 years: 2  More than 5 years: 7  Pain location  Legs (unspecified): 5.  Hips: 4  Knees: 5  Feet/toes: 9  Shoulder/neck: 7  Hands/wrists: 3  Back/torso: 3  Stomach: 3  **Control group (*n* = 9),** median (range)  Age (years): 15 (14 to 17)  Gender: 4 boys, 5 girls  Beighton score (0-9): 4 (2 to 9)  BMI (kg/m^2^): 20 (19 to 24)  Reported occasions of planned physical activity/week: 0 (0 to 2)  Pain intensity: Over the past week: 0 (0 to 3) At the time of the assessment: 0 (0)  Pain duration/frequency No pain: 4  Less than 3 months: 1  1.5 years: 2  More than 5 years: 2    **Control group**  Knees: 3  Feet/toes: 1  Shoulder/neck: 1  Hands/wrists: 1  Stomach: 1 Other areas: 0 | **Test stimulus: Pressure pain** Pressure pain threshold (PPT) was assessed at the trapezius using a hand-held pressure algometer with a 1-cm^2^ rubber tip and an application rate of 1 kg/s. Participants said ‘stop’ as soon as the pressure sensation changed to discomfort. Three trials were conducted, with the mean of the last two trials taken as pressure pain threshold.  **Conditioning stimulus: Cold pressor test** Participants places their non-dominant hand in circulating cold water with a constant temperature of 12 °C±1 for one minute.  **Procedure** PPT was administered before and two during the conditioning stimulus (20 and 50 seconds after immersion of the hand in the cold water). | **Primary and Secondary Hyperalgesia**  PPTs assessed using hand-held pressure algometer at symptomatic test site (trapezius muscle) and three remote test sites (triceps muscle, lower back, and tibialis anterior muscle (upper and lower quadrant and axial) via the procedure described.  **Exercise-Induced Hypoalgesia** PPT was assessed immediately after performing an exercise test on a bicycle ergometer. The exercise was interrupted when participants reached 75% of their maximum heart rate or when they reported a perceived exertion rating of 15 on the Borg Rating of Perceived Exertion Scale  **Numerical rating scale** The average pain intensity caused by immersion in cold water was measured using an 11-point NRS. Pain intensity over the past week and at the time of assessment was also assessed, although not explicitly stated if this was an 11-point scale. | \| **Measurements** \| **Patient Group (*n* = 10), median (range)** \| **Control group (*n* = 9), median (range)** \| \| --- \| --- \| --- \| \| PPT—trapezius muscle (kg/cm2) \| 2.01 (1.52 to 5.04) \| 3.03 (1.3 to 5.05) \| \| PPT—deltoideus muscle (kg/cm2) \| 1.94 (0.67 to 3.28) \| 1.8 (1.06 to 5.53) \| \| PPT—tibialis anterior (kg/cm2) \| 3.02 (2.05 to 7.31) \| 4.53 (1.58 to 7.79) \| \| PPT—lower back (L3) (kg/cm2) \| 2.85 (1.56 to 6.88) \| 3.71 (1.94 to 10.54) \| \| CPM outcome (kg/cm2) \| 0.6 (− 0.23 to 1.23) \| 0.5 (− 0.82 to 1.33) \| \| EIH outcome—trapezius muscle (kg/cm2) \| 0.05 (− 0.48 to 2.01) \| 0.27 (− 1.17 to 0.59) \| \| EIH outcome—tibialis anterior (kg/cm2) \| 0.44 (− 1.3 to 2.91) \| 0.04 (− 1.51 to 1.01) \| \| Borg RPE (6–20) \| 16.5 (15 to 19) \| 14 (13 to 15) \| \| Cycling duration (min) \| 5 (4 to 6) \| 4 (3 to 5) \| \| NRS—cold pressure test \| 5.45 (2.3 to 6.8) \| 1.0 (0 to 7.8) \|   When assessing CPM, two patients and three healthy adolescents did not achieve a pain experience (NRS≥3) when immersing their hands in the cold water. |
| **Stolzman & Bement (2016)** USA | **32 normal weight adolescents and 24 overweight/obese adolescents**  Normal weight: Age = 16.0 (1.8); 17 male; BMI = 21.3 (1.8)  Overweight/obese: Age = 14.5 (1.7); 10 male; BMI = 30.9 (7.0) | **Test Stimulus: Pressure Pain**  Assessed via algometer with a 1cm^2^ probe at a rate of 50 kPa/s. Pressure applied to the left 4th digit nailbed and left middle deltoid muscle. Participants indicated when they first felt pain. Three trials were completed at each site with a 10-second inter-stimulus interval.  **Conditioning Stimulus: Cold Pressor Test**  Right foot immersed in water bath maintained at 1-2°C  **Neutral Control Stimulus: Cool water**  Right foot immersed in water bath maintained at 24-25°C (distraction control)  **Procedure**  Pressure pain threshold were assessed during the neutral, cool water bath condition and after twenty minutes during the painful conditioning stimulus.  Participants completed three sessions approximately one week apart. Questionnaires, weight and pressure pain thresholds were collected. During session 2 or 3 the CPM protocol was administered along with body composition testing. | **Pain intensity: Numeric Rating Scale**  Scored from 0-10 (0 = ‘no pain’, 10 = ‘worst pain’).  **Pain assessment**  McGill Pain Questionnaire  **Weight Status & Body Composition**  Calibrated stadiometer used to measure height. Calibrated standing scale used to measure weight.  **Tanner Staging**  Tanner staging questionnaire used to determine sexual maturation.  **Physical Activity**  Self-reported physical activity measured using the physical activity questionnaire-Elementary School & High School Versions (PAQ)  **Exercise Induced Hypoalgesia (EIH)**  Pressure pain threshold measure at left nailbed, left deltoid and left quadriceps before and after a maximal aerobic treadmill test. EIH was calculated as the increase in PPT after exercise. | \|  \| **All Participants  (n = 56)** \| \| --- \| --- \| \| **Nailbed** \|  \| \| PPT Cool water \| 451.9 ± 211.8 \| \| PPT Ice Water \| 528.2 ± 230.7 \| \| Absolute CPM \| 76.3 ± 99.5 \| \| Relative CPM \| 21.8 ± 28.2 \| \| **Deltoid** \|  \| \| PPT Cool water \| 375.1 ± 258.5 \| \| PPT Ice Water \| 466.5 ± 293.2 \| \| Absolute CPM \| 91.4 ± 122.4 \| \| Relative CPM \| 33.8 ± 44.7 \| \|  \|  \| \| Foot pain 20s cool water \| 0.2 ± 0.7 \| \| Peak foot pain cool water \| 0.3 ± 0.8 \| \| Foot pain 20s ice water \| 5.6 ± 2.5 \| \| Peak foot pain ice water \| 7.0 ± 2.5 \|  \|  \| **Normal Weight  (n = 32)** \| **Overweight/Obese**  **(n = 24)** \| \| --- \| --- \| --- \| \| **Nailbed** \|  \|  \| \| PPT Cool water \| 453.6 ± 201.0 \| 449.6 ± 229.7 \| \| PPT Ice Water \| 549.1 ± 235.2 \| 500.4 ± 226.5 \| \| Absolute CPM \| 95.5 ± 88.5 \| 50.8 ± 109.2 \| \| Relative CPM \| 22.5 ± 20.9 \| 20.8 ± 36.3 \| \| **Deltoid** \|  \|  \| \| PPT Cool water \| 374.9 ± 277.9 \| 375.4 ± 236.1 \| \| PPT Ice Water \| 457.7 ± 310.6 \| 478.2 ± 274.5 \| \| Absolute CPM \| 82.8 ± 128.6 \| 102.8 ± 115.2 \| \| Relative CPM \| 28.3 ± 44.4 \| 41.1 ± 45.0 \| \|  \|  \|  \| \| Foot pain 20s cool water \| 0.3 ± 0.8 \| 0.0 ± 0.2 \| \| Peak foot pain cool water \| 0.4 ± 1.0 \| 0.1 ± 0.4 \| \| Foot pain 20s ice water \| 5.6 ± 1.8 \| 5.6 ± 3.2 \| \| Peak foot pain ice water \| 7.3 ± 2.0 \| 6.7 ± 3.0 \|  \|  \| **Boys (n = 27)** \| **Girls (n = 29)** \| \| --- \| --- \| --- \| \| **Nailbed** \|  \|  \| \| PPT Cool water \| 468.8 ± 228.0 \| 436.1 ± 198.2 \| \| PPT Ice Water \| 549.4 ± 246.4 \| 508.5 ± 217.6 \| \| Absolute CPM \| 80.6 ± 94.9 \| 72.4 ± 105.1 \| \| Relative CPM \| 24.1 ± 32.2 \| 19.7 ± 24.4 \| \| **Deltoid** \|  \|  \| \| PPT Cool water \| 409.1 ± 302.1 \| 343.5 ± 210.7 \| \| PPT Ice Water \| 534.5 ± 349.9 \| 403.2 ± 215.8 \| \| Absolute CPM \| 125.4 ± 140.3 \| 59.7 ± 94.8 \| \| Relative CPM \| 40.3 ± 47.1 \| 27.8 ± 42.2 \| \|  \|  \|  \| \| Foot pain 20s cool water \| 0.4 ± 0.9 \| 0.1 ± 0.2 \| \| Peak foot pain cool water \| 0.4 ± 0.9 \| 0.2 ± 0.7 \| \| Foot pain 20s ice water \| 5.9 ± 2.4 \| 5.3± 2.6 \| \| Peak foot pain ice water \| 7.0 ± 2.4 \| 7.0 ± 2.6 \|   The change in pain thresholds differed between sessions (*p* = .04). No change in PPTs were found after quiet rest (*p* > .05), although these increased in the CPM session. PPTs were greater while the foot was in the ice water bath than the cool water bath (*p* < .0001). This response was similar between the nailbed and deltoid muscle (*p* = .12)  Conditioned pain modulation at the left deltoid was significantly correlated with left arm lean mass (*r* = 0.34, *p* = 0.01) but not fat mass. Nailbed CPM was significantly correlated with physical activity (*p* < .01, *r* = .34)  **Hierarchical Regression Analysis**  Left arm lean mass was a significant predictor of Deltoid CPM (*p* = .01) accounting for 10% of the change. Adolescents with greater lean mass of the arm demonstrated greater CPM magnitude at the deltoid muscle testing site. |
| **Teles, Ocay et al., (2019)** Canada | **Children with idiopathic scoliosis and chronic back pain**  N = 94  Age 15.1 ± 2.1  13 Male, 81 Female  81 Caucasian  7 Black/African American  1 Asian  1 Middle eastern  4 Interracial  Lenke Classification:  1 = 26  2 = 6  3 = 13  4 = 10  5 = 23  6 = 16  Frequency of Pain, n (%)  Daily: 52 (55.32)  Every 2nd Day: 25 (26.60  Once a week: 16 (17.02)  Once a month: 1 (1.06)    Pain Duration, n (%)  3-6m: 6 (6.38)  6-12m: 16 (17.02)  >12m: 72 (76.60)    Duration of painful episodes, n (%)  Few seconds: 3 (3.19)  Few minutes: 21 (22.34)  One hour: 25 (26.60)  Constant: 45 (47.878) | **Test stimulus: Heat pain**  Assessed via 3x3cm thermode applied to the left volar forearm to reach a temperature corresponding to a pain intensity of 5/10. Once the target temperature was reached, it remained constant for 120 seconds. Baseline temperature of 32°C which increased 0.3°C/second. Participants evaluated their pain with a computerized visual analogue scale (CoVAS) ranging from 0 to 100. Average pain intensity was calculated.  **Conditioning Stimulus: Cold pressor test**  Immersion of their right forearm cold water (12°C) for 120 seconds.    **Procedure**  Heat pain intensity was assessed before and immediately after the conditioning stimulus. | **Pain: Faces Pain Scale (Revised)**  Pain intensity during the prior month was reported by the patient using a numerical rating score of 0 – 10  **Duration & Frequency of Pain: Interview**  **Location of pain: segmented diagram**  **Neuropathic Pain**  Douleur Neuropathique 4 (DN4)  **Functional Disability**  Functional Disability Index (FDI)  **Depression & Anxiety**  Revised Child Anxiety and Depression Scale (RCADS)  **Sleep Quality**  Pittsburgh Sleep Quality Index (PSQI)  **Quantitative sensory testing**  Mechanical and thermal QST was performed on the left volar forearm and the most painful region on the back.  *Mechanical detection threshold (MDT).* Assessed via calibrated von Frey filaments.  *Pressure pain threshold (PPT).* Assessed via handheld algometer.  *Heat pain threshold (HPT)and heat pain tolerance (HTT)*  Assessed by a 9cm^2^ thermode (Q-Sense) with a baseline 32°C, 0.3°C/second upslope was applied three times.  *Temporal summation of pain (TSP)*  Assessed via the heat pain trial, whereby participants constantly rated their pain over 120 seconds via CoVAS (as described). | \|  \| **M** \| **SD** \| \| --- \| --- \| --- \| \| **Mechanical Detection Threshold** \|  \|  \| \| Control site \| 0.712 \| 2.35 \| \| Affected site \| 0.620 \| 1.22 \| \| **Pressure Pain Threshold** \|  \|  \| \| Control site \| 27.47 \| 12.68 \| \| Affected site \| 25.58 \| 16.85 \| \|  \|  \|  \| \| Heat Pain threshold \| 39.24 \| 3.32 \| \| Heal Tolerance threshold \| 45.04 \| 2.38 \| \|  \|  \|  \| \| CPM Efficacy \| -27.55 \| 42.86 \|   75% of adolescents with idiopathic scoliosis had lower pressure pain thresholds at affected area than control area, with 36.3% of patients having at least 50% lower pain thresholds at the affected area than the control area.  Efficient pain inhibitory response was observed in 51.1% of patients, 21.3% had sub-optimal and 27.7% had inefficient CPM.  Temporal summation of pain was observed in 11.7% of patients.  **Additional Outcomes**  Significant association between curve type and location of pain (*p* = .05). 60.9% of patients with thoracolumbar/lumbar curves reported pain at the lower back compared to 15.6% of patients with main thoracic or double thoracic curve types.  Weak significant correlation between deformity severity and back PPT (*r* = -0.234, *p* = .023); the larger the Cobb angle, the lower the PPT.  Weak significant correlation CPM and deformity severity (*r* = 0.245, *p* = .017); the larger the Cobb angle, the less efficient the CPM.  Association between DN4 pain scores and back PPT (*r* = -0.250, *p* = .015); the more sensitive is the back, the higher the neuropathic pain score.  Negative association between pain intensity scores and HTT (*r* = −0.205, *p* = .048); higher pain scores correlate to lower heat tolerance.  Neuropathic pain score correlated with temporal summation of pain (*r* = .206; *p* = .047).  Significant correlation between pain intensity and disability (*r* = .209, *p* = .043), and sensory (*r* = .270, *p* = .009) and affective (*r* =0.214, *p* =.038) pain descriptors.  DN4 scores positively correlated with disability (*r* = .321, *p* = .002), anxiety and depression scores (*r* =.250, *p* =.015), and sensory (*r* =0.332, *p* =.001) and affective (*r* =0.246, *p* =.017) pain descriptors.  Disability correlated with higher anxiety and depression scores (*r* = .339, *p* = .001), and sensory (*r* = .337, *p* = .001), affective (*r* = .449, *p* <.0001), and evaluative (*r* = .336, *p* <.0001) pain descriptors. |
| **Tham, Li et al. (2024)** USA | **Adolescents with functional abdominal pain (n = 77)**  Age (years): Mean (SD) = 16.6 (1.4), Range: 14.02-18.98  Sex, n (% female): 66 (85.7%)  Adolescent race, n (%):  Asian: 5 (6.5%)  Black: 1 (1.3%)  White: 56 (72.7%)  Other: 3 (3.9%)  Multiracial: 12 (15.6%)  Adolescent ethnicity, n (% Hispanic)*: 7 (9.2%)  Caregiver race, n (%)  Asian: 5 (6.5%)  White: 62 (80.5%)  Other: 3 (3.9%)  Multiracial: 7 (9.1%)  Caregiver ethnicity, n (% Hispanic): 5 (6.6%)  Highest parental education, n (%)  High school or less: 7 (9.2%)  Vocational or trade school/some college or university: 18 (23.7%)  College or university: 20 (26.3%)  Graduate degree or professional school: 31 (40.8%)  Household income, n (%)  < $50, 000: 16 (21.3%)  $50,000 to $149,999: 34 (45.3%)  ≥150,000: 25 (33.3%)  Usual pain intensity, mean (SD): 5.2 (2.1)  Worst pain intensity, mean (SD): 7.0 (2.0) | **Test stimulus: Heat pain threshold** Assessed via Medoc QSense System (Thermal Sensory Analyzer 2001) somatosensory stimulator with a 30 × 30mm thermode. Heat applied to the volar surface of the forearm at baseline temperature of 32 °C, increasing by 1.5 °C/s to a maximum of 50 °C. Participants pressed a button the first moment pain was experienced. Heat pain threshold was averaged from the last three of four trials.  **Conditioning stimulus: Cold pressor test** Participants submerged their nondominant hand and wrist in a 10°C circulating water two minutes (or less if not tolerated).  **Procedure** Heat pain threshold was assessed before and during the cold pressor task. Participants completed three trials during the cold-pressor task. | **Abdominal Pain Index** Abdominal pain severity was assessed via the four-item Abdominal Pain Index.  **Child Activity Limitation Interview-21** This is a 21-item measure evaluating difficulty with typical childhood activities over the past four weeks, such as going to school, running, doing chores.  **Pain Catastrophizing Scale for Children**  A 13-item measure of pain catastrophising with subscales of rumination, magnification, and helplessness.  **Pain Rating** The participants used the 11-point NRS to rate their pain following each task of the QST protocol.  **Pressure Pain Threshold** Assessed via handheld computerized pressure algometer with a 1 cm^2^ probe (AlgoMed, Medoc Ltd applied to the) was forearm. Pressure was increased at 35 kPa per second to the maximal pressure of 1,000 kPa. Adolescents pressed the button when they first experienced pain. Four trials were conducted with the average of the last bthree trials used in the analyses.  **Pressure Pain Tolerance**  Assessed via handheld computerized pressure algometer with a 1 cm^2^ probe. Adolescents pressed the button when they were not able to tolerate the pain.  **Heat Pain Threshold** Assessed via Medoc QSense System, as discussed. | **Clinical Characteristics and QST Values of the Study Sample (N=77)**   \| **Characteristics** \| **Values** \| \| --- \| --- \| \| API score, mean (SD) \| 2.5 (.9) \| \| Pain interference (CALI) \| 31.8 (17.0) \| \| Pain catastrophizing level (PCS-C)  Low catastrophizing (0–14)  Moderate catastrophizing (15–25)  High catastrophizing (≥26) \| 22.7 (10.5)  19 (24.7%)  23 (29.9%)  35 (45.4%) \| \| Pressure pain threshold: kPa \| 178 (89) \| \| Pressure pain tolerance: kPa \| 357 (166) \| \| Heat pain threshold: °C \| 43.1 (3.3) \| \| Conditioned pain modulation index \| 100.6 (5.9) \| \| Pressure pain threshold pain rating (0–10) \| 3.9 (2.0) \| \| Pressure pain tolerance pain rating (0–10) \| 6.5 (1.8) \| \| Heat pain threshold pain rating (0–10) \| 4.8 (2.1) \|   **Correlations between QST Measures, PCS-C, and Clinical Pain Severity (N=77)**   \|  \| **Pain catastrophising (PCS-C)** \| **Abdominal pain intensity (0-10)** \| **Pain-related interference (CALI)** \| \| --- \| --- \| --- \| --- \| \| **Pressure pain threshold** \| −.06 \| .20 \| −.01 \| \| **Pressure pain tolerance** \| −.06 \| .08 \| −.04 \| \| **Heat pain threshold** \| .31* \| .24** \| .18 \| \| **Conditioned pain modulation index** \| −.21 \| −.25** \| −.21 \| \| **Pressure pain threshold pain rating** \| .24** \| .35* \| .26** \| \| **Pressure pain tolerance pain rating** \| .27** \| .18 \| .27** \| \| **Heat pain threshold pain rating** \| .36** \| .44*** \| .28** \|   Abbreviations: PCS-C, Pain Catastrophizing Scale-Child; CALI, Child Activity Limitations Interview. * *p* < 0.01; ** *p* < .05; ****p* < .001.  **Age and Sex-adjusted Associations Between QST and Clinical Pain Outcomes**   \| QST (Standardised with mean of 0 and SD of 1) \| Abdominal pain intensity (0-10 NRS) \| \| Pain-related interference (CALI) \| \| \| --- \| --- \| --- \| --- \| --- \| \|  \| Beta (95%CI) \| *p* \| Beta (95%CI) \| *p* \| \| Pressure pain threshold \| .32 (−.15, .79) \| .18 \| −1.1 (−5.1, 2.9) \| .59 \| \| Pressure pain tolerance \| .12 (−.36, .61) \| .62 \| −1.0 (−5.0, 2.9) \| .60 \| \| Heat pain threshold \| **.54 (.07, 1.02)** \| **.026** \| 2.7 (−1.4, 6.8) \| .19 \| \| Conditioned pain modulation index \| −.33 (−.83, .18) \| .20 \| −3.8 (−8.0, .4) \| .072 \|   Abbreviation: CALI, Child Activity Limitations Interview. NOTE. Bold for significant associations.  **Effect Modification by Adolescent Pain Catastrophizing in the Relationship Between QST and Clinical Abdominal Pain Outcomes, Adjusted for Age and Sex**   \|  \| Abdominal pain intensity (0-10 NRS) \| \| Pain-related interference (CALI) \| \| \| --- \| --- \| --- \| --- \| --- \| \|  \| Beta (95%CI) \| *p* \| Beta (95%CI) \| *p* \| \| **Pressure pain threshold (PPTh)**  PPTh (z)  PCS-C (z)  PPTh (z) × PCS-C (z) \| .34 (−.10, .78)  **.81 (.36, 1.26)**  .22 (−.24, .67) \| .12  **.0006**  .35 \| −1.0 (−4.5, 2.6)  **7.6 (4.0, 11.3)**  **3.9 (.2, 7.6)** \| .59  **< .0001**  **.039** \| \| **Pressure pain tolerance (PPTl)**  PPTl (z) PCS-C (z) PPTl (z) × PCS-C (z) \| .32 (−.16, .79)  **.85 (.40, 1.30)**  **.48 (.00, .95)** \| .19  **.0004**  **.049** \| 1.1 (−2.5, 4.8)  **8.1 (4.6, 11.6)**  **5.4 (1.8, 9.0)** \| .54  **< .0001**  **.004** \| \| **Heat pain threshold (HPTh)**  HPTh (z)  PCS-C (z)  HPTh (z) × PCS-C (z) \| .29 (−.19, .77)  **.67 (.19, 1.16)**  −.31 (−.78, .15) \| .23  **.007**  .18 \| .6 (−3.4, 4.7)  **7.6 (3.5, 11.7)**  .6 (−3.3, 4.9) \| .76  **.0004**  .77 \| \| **Conditioned pain modulation index**  CPM index (z) PCS-C (z) CPM index (z) × PCS-C (z) \| −.16 (−.64, .32)  **.78 (.32, 1.24)**  .29 (−.19, .77) \| .51  **.001**  .23 \| −2.6 (−6.5, 1.4)  **7.5 (3.7, 11.3)**  .5 (−3.4, 4.5) \| .20  **.0002**  .80 \|   Abbreviations: CALI, Child Activity Limitations Interview; PCS-C, Pain Catastrophizing Scale-Child. Note: QST parameters and PCS-C scores were standardized with a mean of 0 and standard deviation of 1; significant effect sizes were bold. Bold for significant regression coefficients. |
| **Tsao, Seidman et al., (2013)** USA | **Healthy children and adolescents**  n = 124  Aged 8-17y (13.0 ± 2.9)  60 Male, 64 Female  39 Hispanic/Latino  85 Non-Hispanic/Latino  55 White  30 African American  2 Asian  1 American Indian/Alaska Native  33 Multi-Racial.  Sample divided into children (8-11 year olds) and adolescents (12-17 year olds) | **Test Stimulus: Pressure Pain**  Five second application of pressure via a 1x1cm rubber probe attached to a computer control hydraulic piston, applied to the left thumbnail. Ascending pressure stimuli were presented at a rate of .132kg/cm^2^ with a baseline of .066kg/cm^2^. Stimulus presentation ceased when the participant indicated moderate pain (6/10 on the NRS).  Stimuli were then presented at 15 second intervals in a random order using the multiple random staircase method. The two staircases were presented simultaneously (mild pain -3  on the NRS, moderate pain - 6 on the NRS). The final 4 measurements on the high staircase were averaged to produce a final value of moderate pressure pain threshold.  **Conditioning Stimulus: Cold Pressor**  Right hand submersion (up to 2inches above the wrist joint) in a water bath maintained at 5°C for up to 30 seconds or until the participant was unable to tolerate any longer.  **Procedure**  The test stimulus (TS) was administered initially on its own as a bassline rating (TS1). Participants were then administered the cold pressor task. With the right hand immersed in the cold water, the TS was administered a second time (TS2). Fifteen seconds after removal of the hand from the cold water, the TS was administered a third time (TS3). Lastly the TS was administered a fourth time (TS4) 50 seconds later. TS pain ratings using the NRS recorded immediately following each 5 second TS administration. | **Pain intensity: Numeric Rating Scale (NRS)**  Participants rated pain on a 0-10 scale, (0 = ‘none’, 10 = ‘worst or most possible’).    **Pain Catastrophising**  Pain Catastrophizing Scale for Children  (PCS-C)    **Pain Anxiety**  Child Pain Anxiety Symptoms Scale (CPASS)  **Bodily Pain**  Bodily pain (BP) subscale of the Child Health Questionnaire (CHQ)  **Heart Rate Variability**  Two lead ECG. Recorded for 5-minutes pre-task and after the completion of the CPM task. The extent of heart rate variability was assessed by calculation of the root mean square of successive differences (rMSSD). | **Descriptive Statistics:**   \|  \| **Total Sample** \| **Boys** \| **Girls** \| \| --- \| --- \| --- \| --- \| \| TS1 \| 5.35 (2.1) \| 5.32 (2.2) \| 5.38 (1.9) \| \| TS2 \| 3.81 (2.0 \| 3.83 (2.1) \| 3.80 (2.0) \| \| TS3 \| 4.23 (2.1) \| 4.50 (2.4) \| 3.98 (1.9) \| \| TS4 \| 5.01 (1.9) \| 4.97 (2.0) \| 5.05 (1.8) \| \| Absolute CPM \| -1.53 (2.1) \| -1.48 (2.0) \| -1.58 (2.2) \| \| CPM % Change \| -23.0% (41.8) \| -21.8% (45.5) \| -24.2 (38.3) \| \| Anticipatory Anxiety \| 4.15 (2.9) \| 4.12 (3.0) \| 4.17 (2.8) \| \| PCS-C \| 15.3 (8.0) \| 15.0 (8.2) \| 15.7 (7.9) \| \| CPASS \| 26.1 (16.1) \| 25.7 (16.8) \| 26.4 (15.5) \| \| CHQ BP Subscale \| 76.2 (19.2) \| 75.6 (17.7) \| 76.8 (20.5) \| \| **rMSDD** \|  \|  \|  \| \| Pre-Task \| 63.4 (38.9) \| 65.9 (39.1) \| 61.2 (39.0) \| \| Post-task \| 61.4 (41.4) \| 61.4 (35.2) \| 61.3 (46.7) \|   rMSDD = Root mean square of successive differences   \|  \| **Children** \| **Adolescents** \| \| --- \| --- \| --- \| \| TS1 \| 5.66 (2.1) \| 5.18 (2.0) \| \| TS2 \| 4.68 (2.2) \| 3.34 (1.8) \| \| TS3 \| 4.93 (2.3) \| 3.85 (2.0) \| \| TS4 \| 5.72 (2.0) \| 4.62 (1.7) \| \| Absolute CPM \| -.98 (2.4) \| -1.84 (1.9) \| \| CPM % Change \| -9.2% (44.5) \| -30.6% (38.4) \| \| Anticipatory Anxiety \| 4.70 (3.0) \| 3.84 (2.8) \| \| PCS-C \| 15.2 (7.0) \| 15.4 (8.6) \| \| CPASS \| 28.5 (17.1) \| 24.7 (15.5) \| \| CHQ BP Subscale \| 81.1 (18.6) \| 73.5 (19.1) \| \| **rMSDD** \|  \|  \| \| Pre-Task \| 65.4 (40.7) \| 62.3 (38.2) \| \| Post-task \| 64.5 (42.5) \| 59.7 (40.9) \|   rMSDD = Root mean square of successive differences  Main effect of time (F(1, 120) = 50.91, *p* < .001), with TS2 ratings significantly lower than TS1.  Main effect of Age ( F(1, 120) = 8.01, *p* < .01), with younger children reporting greater TS pain compared to older children  Significant Time x Age interaction (F(1, 120) = 4.70, *p* < .04).  Significant effect of Time for both younger (F(1,122) = 9.68, *p* < .01) and older (F(1,122) = 62.19, *p* < .001).  TS ratings during CS delivery were significantly lower in older children than younger children (F(1,122) = 13.62, *p* < .01).  Pressure force administered to younger children (*m* = 0.42, SD = 0.27) was significantly lower than the amount administered to older children (*m* = 0.69, SD = 0.35) (*t*(122) = −4.46, *p* < . 001)  **Exploratory analysis: Durability of CPM effects**  Main effect of Time (F (3,117) = 18.36, *p* < .001), and of Age (F(1,119) = 11.36, *p* < . 01).  Pairwise Bonferroni adjusted comparisons of the TS ratings indicated that TS1 was significantly different from TS2 (*p* < .001), and TS3 (*p* < .001), and TS4 was significantly different from TS2 (*p* < .001), and TS3 (*p* < .001)  **Additional Outcomes**  CPM magnitudes expressed as % change was negatively correlated with pre-task baseline rMSSD (*r* = −.22, *p* < .02), there was a trend toward a relationship for CPM absolute values (*r* = −.18, *p* = .053).  Post-task rMSSD values were significantly negatively correlated with CPM magnitudes expressed both as percent change (*r* = −.22, *p* < .02), and as absolute values (*r* = −.21, *p* < .03).  CPM anticipatory anxiety was significantly correlated with the PCS-C (*r* = .29, *p* < .01), and the CPASS (*r* = .26, *p* < .01). |
| **Uzawa, Takeuch et al. (2024)** Japan | **Female (*n* = 14)**  Age (years): 21.1 +/- 0.6  Body mass index (kg/m^2^): 20.0 +/- 1.5  Experience of orthopaedic surgery, *n* (%): 4 (28.6)  **Males (*n* = 18)**  Age (years): 20.9 +/- 0.3  Body mass index (kg/m^2^): 21.3 +/- 2.3  Experience of orthopaedic surgery, *n* (%): 2 (11.1) | **Test stimulus: Pressure pain** Assessed via pressure algometer (Wagner FPX-25), measuring pressure pain threshold (PPT) in the upper fibres of the left trapezius.  **Conditioning stimulus: Cold pressor test** Participants immersed their right hand in the cold water (average of 10 ̊C, ranging from 8 ̊C to 12 ̊C) for 2 minutes. Test stimuli were performed after rest and cold periods using a pressure algometer (Wagner FPX-25; Wagner Instruments, Greenwich, CT) to measure the pressure pain threshold (PPT) in the upper fibres of the left trapezius.  **Procedure** Participants were first seated for ten minutes in a rest period. Following this, they completed the first PPT assessment. Participants then immersed their hand ion the cold water, after which they completed a second PPT assessment. CPM was determined as the ratio between the PPT after the cold period and the PPT after the rest period. | **Pain Catastrophising Scale**  Evaluate pain catastrophising, an exaggerated negative feeling caused by an actual or anticipated painful experience.  **Hospital Anxiety and Depression Scale**  Assess anxiety and depression, with 7 questions on each test.  **Pittsburgh Sleep Quality Index**  A subjective measure of sleep quality and sleep disorders.  **International Physical Activity Questionnaire**  Assess the amount of physical activity: a higher score indicates a higher amount of physical activity.  **Checklist for Individual Strength**  Assess fatigue severity and fatigue-related symptoms.  **The autonomic nervous system was assessed using heart rate (HR) and blood pressure.**  A wearable electrocardiogram was attached to the participant’s chests to continuously measure beat-to-beat intervals during rest and cold periods. Blood pressure was measured using a digital sphygmomanometer for systolic and diastolic blood pressure. | **Pain catastrophising scale**  Female: 15.1 +/- 9.3  Male: 13.1 +/- 8.3  **Hospital Anxiety and Depression Scale (Anxiety)**  Female: 5.1 +/- 2.7  Male: 4.9 +/- 4.0  **Hospital Anxiety and Depression Scale (Depression)**  Female: 5.2 +/- 2.8  Male: 5.7 +/- 3.5  **Pittsburgh Sleep Quality Index**  Female: 5.8 +/- 2.7  Male: 5.2 +/- 2.2  **International Physical Activity Questionnaire**  Female: 24.4 +/- 25.0  Male: 25.0 +/- 22.2  **Checklist for Individual Strength**  Female: 77.0 +/- 8.3  Male: 80.0 +/- 9.3  **Group differences in conditioned pain modulation and autonomic indices (mean and SD)**   \| Variables \| Female (*n* = 14) \| Male (*n* = 18) \| *p* \| \| --- \| --- \| --- \| --- \| \| CPM Index (%) \| 127.0 +/- 19.1 \| 124.0 +/- 18.7 \| .661 \| \| LF/HF Index (%) \| 128.8 +/- 56.8 \| 119.2 +/- 35.2 \| .558 \| \| LFnu Index (%) \| 111.7 +/- 17.4 \| 104.6 +/- 9.2 \| .189 \| \| HFnu Index (%) \| 91.5 +/- 21.5 \| 92.4 +/- 20.6 \| .908 \| \| SBP Index (%) \| 110.6 +/- 8.6 \| 107.9 +/- 9.7 \| .414 \| \| DBP Index (%) \| 116.5 +/- 17.9 \| 116.7 +/- 27.6 \| .973 \| \| HR Index (%) \| 99.7 +/- 7.0 \| 99.7 +/- 7.8 \| .992 \|   *Abbrevations*. CPM, conditioned pain modulation; DBP, diastolic blood pressure; HFnu, high frequency normalised unit; HR, heart rate; LFnu, low frequency normalised unit; SBP, systolic blood pressure.  **Conditioned Pain Modulation**  Conditioned pain modulation indices were 127.0 6 19.1% in females and 124.0 6 18.7% in males, with no statistical difference between the groups (*p* = .661). No group differences were identified in the LF/HF, LFnu, HFnu, SBP, DBP, and HR indices.    Females and males showed a similar inhibitory effect after cold-water immersion. This result did not correspond with our first hypothesis, which was that females would show a lower CPM effect.  Sympathetic and parasympathetic nervous systems activities across the whole sample were significantly associated with CPM effects.  Only the LFnu and LF/HF indices in females were significantly associated with the CPM index (*p* =.008 and *p* = .004 respectively). |
| **Verriotis et al. (2021)**  UK | **52 adolescents with neuropathic pain and 14 adolescents with complex region pain syndrome**  Data for whole sample  Mean age: 14.9 years (12.9-16.1) median and IQR; 23 male and 43 female  Pain pathology   - Peripheral nerve injury associated with previous surgery 32 - Peripheral neuropathic pain other causes 20 - CRPS14  \| Pain intensity (VAS 0-10) \| M \| SD \| \| --- \| --- \| --- \| \| Pain now \| 4.6 \| 2.2 \| \| Average pain last week \| 6.3 \| 1.9 \| \| Worst pain last week \| 7.6 \| 2.0 \| \| Interference due to pain \| 6.9 \| 2.7 \|   Pain duration (n)   - 3-12 months 12 - 1-2 years 16 - 2-5 years 25 - >5 years 13   Neuropathic pain site (n)   - Head and neck 11 - Trunk 10 - Upper limb 7 - Lower limb 38   Neuropathic pain distribution (n)   - Unilateral 46 - Bilateral 20  \|  \| Median \| IQR \| \| --- \| --- \| --- \| \| S-LANSS average pain last week (NRS 0-10) \| 7 \| 7-8 \| \| S-LANSS total \| 19 \| 13-23 \| \| McGill Pain Questionnaire Sensory (0-33) \| 15 \| 12-18.5 \| \| McGill Pain Questionnaire Affective (0-12) \| 4 \| 2-6 \| \| PedsQL (0-100) \| 47 \| 35-57 \| \| PI-ED (0-42) \| 17 \| 11-22 \| \| PCS-C (0-52) \| 30 \| 20-39 \| | **Test stimulus: Pressure pain**  Pressure pain threshold (PPT) assessed on the head of the fibula (lateral knee) via computerized algometer (1cm^2^ tip, ramp 40 kPa/second), with the mean of three trials computed.  **Conditioning stimulus: Cold pressor test** Immersion of the contralateral hand in 5 ̊C cold circulating water for 30 seconds or until the stimulus became too unconformable. Participants rated their pain on a 0-10 NRS after removal.  **Procedure** The test stimulus was assessed at baseline, during the conditioning stimulus (after 15 seconds immersion) and 50 and 90 seconds after initial immersion. PPT assessed on the right knee with the left hand used for conditioning, although this was reversed if the participant reported pain in the left hand or right knee. | **Pain intensity and interference**  VAS 0-10cm assessing current pain, average pain in the last week, worst pain in the last week, and pain interference with usual activities.  **Anxiety and depression**  Pediatric Index of Emotional Distress (PIED)  **Quality of life** Paediatric Quality of Life Inventory Child (PedsQL-C) and Paediatric Quality of Life Inventory Parent tools  **Emotional distress** Pediatric Index of Emotional Distress (PI- ED)  **Pain catastrophising** Pain Catastrophizing Scale Child Version (PCS-C)  **Parental catastrophising** Pain Catastrophizing Scale Parent Version  **Neuropathic symptoms**  Self-report version of the Leeds Assessment of Neuropathic Symptoms and Signs (S-LANSS)  **Quantitative Sensory Testing**  Testing was performed on the following locations: thenar eminence of nondominant hand; dynamic mapping towards the region of pain; static testing at the site of maximal pain or as close as tolerated, and adjacent to surgical scars; for patients with unilateral pain a contralateral mirror site, for patients with bilateral symptoms a pain-free site within the same body region for patients with bilateral symptoms.  *Cold detection threshold (CDT) and warm detection threshold (WDT), CPT, and heat pain threshold (HPT).*  Assessed via 18x18cm thermode  *Mechanical detection threshold (MDT) and mechanical pain threshold (MPT)* Assessed via calibrated von Frey filaments.  *Pressure pain threshold (PPT).* Assessed via handheld algometer with a 1cm^2^ tip.  *Wind-up ratio* Pain to the MPT stimulus was rated 0 to 10  at the first stimulus (VRS1) and again  following a 1/second train of 10 repeated stimuli (VRS10). Wind-up ration computed as VRS10/VRS1  *Dynamic mechanical allodynia and dynamic allodynia mapping* Assessed via calibrated brush stroke (200-300 mN) | Cool detection threshold - CDT Thernar: 0.46 (0.21) Contralateral: 0.27 (.21) Pain site: 0.59 (0.33  Warm detection threshold - WDT Thernar: 0.50 (0.20) Contralateral: 0.72 (0.20) Pain site: 0.75 (0.25)  Cold pain threshold - CPT Thernar: 19.4 (6.7) Contralateral: 20.6 (6.74) Pain site: 21.8 (6.7)  Heat pain threshold – HPT Thernar: 43.0 (5.0) Contralateral: 43.1 (4.4) Pain site: 42.0 (5.1)  Pressure pain threshold - PPT Thernar: 2.3 (0.30) Contralateral: 2.14 (0.37) Pain site: 2.0 (0.45)  Mechanical pain threshold - MPT Thernar: 2.0 (0.44) Contralateral: 2.0 (0.46) Pain site: 1.6 (0.58)  Mechanical pain to von Frey filament - MP(vF) Thernar: 1.7 (0.71) Contralateral: 1.8 (0.69) Pain site: 1.1 (0.83)  Mechanical detection threshold - MDT Thernar: -0.11 (0.62) Contralateral: 1.83 (0.69) Pain site:0.07 (0.68)  A spectrum of CPM responses was found. Clear inhibition (increased PPT) was evoked in 32 participants, facilitation (decreased PPT) in 8 participants, and 17 participants were classified as nonresponders. Inhibitory CPM was associated with significant increases in pressure pain threshold during (73 +/- 50% at 15 seconds) and after the conditioning stimulus. Facilitation resulted in a significant decrease in parallel pressure pain threshold (-49 +/- 18% at 15 seconds) that did not persist  Males and females did not differ in conditioning tolerance (duration of the immersion in the cold water; males (median [IQR], 30 [17-30]), females (29 [23-30]), nor conditioning pain on removal from the cold water (males (8 [6-10]) and females (8 [IQR7-10]). CPM (absolute change) at 15 seconds in males was 0.07 [95%CI -0.01,0.16]) and in females was 0.10 [95%CI 0.01,0.18]). |
| **Williams, Heitkemper et al. (2013)** USA | **Girls with irritable bowel syndrome (IBS)**  N = 22, mean age 9.8 (1.5), aged 7-12.  **Healthy female controls** N = 21, mean age 9.4 (1.4) | **Test Stimulus: Heat pain**  Assessed via a 30x30mm thermode applied to the volar surface of the right forearm.  Method of limits used to determine pain threshold. Temperature began at 32°C and increased by of 1.5°C per second. Participants indicated when the temperature became pain.  **Conditioning Stimulus: Cold Pressor test**  Left hand immersed in 12 (±1°C) circulating water for up to 60 seconds or for as long as could be tolerated.  **Procedure**  Heat pain threshold assessed at baseline (four trials), and again twenty seconds after immersion in the cold water (three trials). At both times the first trial was excluded, and the average of the other trials computed. | **Pain intensity: Numeric Rating Scale (NRS)**  Participants rated pain on a 0-10 scale (0 = ‘none’, 10 = ‘worst or most possible’).  **Somatization symptoms**  The Children’s Somatization Inventory - revised form (CSI).  **Anxiety and depression**  Behaviour Assessment System for Children (2^nd^ edition) (BASC-2)  **Patient-reported symptom diary: Pain and stooling behaviour.**  14-day paper diary of abdominal pain and stooling frequency and form completed at home by participants. Measures included pain and interference ratings, and stooling patterns. | Final IBS n = 22, n=16 healthy controls  **Descriptive Statistics:**   \|  \| **IBS group** \| **Healthy group** \| \| --- \| --- \| --- \| \| **Endogenous Pain-Inhibition** \|  \|  \| \| Change scores \| -.26 ± 1.79 \| .91 ± 2.55 \| \| EPI + Parent report Psych variables as covariate \| -.04 ± SE = .71 \| .62 ± SE = .63 \| \| EPI + Child report psych variables as covariate \| -.44 ± SE = .69 \| .96 ± SE + .56 \| \| **Psychological Variables** \|  \|  \| \| PR child anxiety \| 60.6 ± 13.9 \| 49.2 ± 9.1 \| \| PR child Depression \| 49.4 ± 10.0 \| 44.1 ± 5.6 \| \| PR child somatisation \| 18.8 ± 11.4 \| 2.6 ± 2.8 \| \| Parent Somatisation \| 51.1 ± 9.3 \| 49.4 ± 10.4 \| \| Parent depression \| 54.2 ± 8.7 \| 48.7 ± 8.8 \| \| Parent anxiety \| 50.1 ± 11.2 \| 46.6 ± 9.9 \| \| CR anxiety \| 53.0 ± 11.9 \| 43.7 ± 6.9 \| \| CR Depression \| 48.3 ± 9.1 \| 44.3 ± 4.9 \| \| CR somatisation \| 29.9 ± 17.1 \| 14.5 ± 14.1 \| \| **Somatic Pain** \|  \|  \| \| Baseline Heat pain Threshold \| 40.0 ± 4.9 \| 39.3 ± 4.6 \| \| Conditioning NRS pain rating \| 7.2 ± 2.6 \| 5.1 ± 3.4 \|   PR = Parent-reported; CR = Child-reported  A significant difference between groups was found in endogenous pain inhibition, which was greater in healthy girls than girls with IBS (*p* = .03, *d* = .71).  Mean IBS group change score = -.26 ± 1.79  Mean healthy group change score = 1.20 ± 2.30  Greater child-reported anxiety (*p* = .01, *d* = .95) and somatization symptoms (*p* = .01, *d* = 1.04) were found in girls with IBS than healthy girls. Higher scores on parent reports of child anxiety (*p* < .01, *d* = 1.06), depressive symptoms (*p* = .03, *d* = .70), and somatization symptoms (*p* < .01, *d* = 1.97) were found in girls with IBS compared to healthy girls.  In the IBS group only, a significant correlation was found (*r* = .47, *p* = .03) between endogenous pain-inhibition and the number of days without a bowel movement (*r* = .47, *p* = .03). |
