# Supplementary material for: A Systematic Review and Meta-Analysis of Conditioned Pain Modulation in Children and Young People with Chronic Pain
Source: Children (Basel). 2024 Nov 11;11(11):1367. doi: 10.3390/children11111367 (PMC11592744; doi:10.3390/children11111367)
Supplement: Supplementary file 1 [file children-11-01367-s001.zip › Table S2 - Outcomes and psychometric properties 05-09-24.docx]

Table S2. Outcome measures and their psychometric properties for studies included in a systematic review and meta-analysis of conditioned pain modulation in children and young people with chronic pain

| **Patient Reported Outcomes** | | |
| --- | --- | --- |
| **Outcome Domain** Outcome Measure | **Psychometric properties reported in the study sample [study]** | **Suitability of outcome measure for paediatric population** |
|  |  |  |
| **Pain Intensity** |  |  |
| Numerical Rating Scale | NR [1-12] | Suitable for assessing acute and chronic pain intensity in paediatric populations as young as 6 years old with good psychometric properties including test-retest reliability, construct validity, and concurrent validity [13]. |
| Visual Analogue Scale | NR [14, 15] | Suitable for assessing pain intensity among children and adolescents aged between 6 and 17 years, with evidence of good psychometric properties including test-retest reliability and internal responsiveness [16]. |
| Modified version of Adolescent Pediatric   Pain Tool [17] | NR [2, 18] | Probably suitable for paediatric populations. Original version has adequate psychometric properties including content, construct and criterion validity and reliability in clinical and nonclinical groups of children and adolescence aged 8-17 years [1, 2, 19]. Modified versions (i) divided the diagram of the back into ten segments, with an NRS then used to rate pain intensity in each [2]; (ii) enlarged the diagram of the back so patients could indicate their pain location, with pain intensity then reported via NRS [18]. |
| Faces Pain Scale – Revised [20] | NR [3, 21] | Suitable for paediatric populations with and without chronic pain, with evidence of good psychometric properties including test-retest reliability, content, and construct validity [20, 22]. |
| Wong-Baker Faces Pain Rating Scale [23] | NR [15] | Suitable for a paediatric population with good psychometric properties including test-retest reliability, concurrent validity and some evidence of discriminant validity [22, 23]. |
| McGill Pain Questionnaire [24] | NR [25] | Probably suitable for adolescent populations. Evidence of adequate psychometric properties to be used as an outcome measure, with some concerns considering the complexity of the questionnaire, indicating a potential issue with the suitability of this measure for a paediatric population [26]. |
| McGill Pain Questionnaire – Short form [27] | NR [14] | Probably suitable for adolescent populations, with evidence of satisfactory reliability and superiority to the original MPQ as a measure of change and responsiveness to treatment [26]. |
| Child Health Questionnaire – Bodily Pain subscale   [28] | NR [29] | Suitable for paediatric populations with and without chronic pain. Reliability and validity tests of the complete measure, compromised of 14 domains, have been extensive with evidence of good psychometric properties [28]. The present paper includes the bodily pain domain exclusively, with no reported psychometric properties [29]. |
| Standardized case report form | NR [14] | Suitability unknown as psychometric properties not reported [14]. |
| Migraine health history form | NR [30] | Suitability unknown as psychometric properties not reported [30]. |
| **Pain Unpleasantness** |  |  |
| Numerical Rating Scale | NR [6] | Suitable for assessing pain unpleasantness in a paediatric population, with evidence of good psychometric properties including convergent validity, discriminant validity and sensitivity to change [31]. |
| **Pain Quality** |  |  |
| Adolescent Pediatric Pain Tool [17] | NR [1, 2] | Suitable for paediatric population with adequate psychometric properties including content, construct and criterion validity and reliability in clinical and nonclinical groups of children and adolescence aged 8-17 years [1, 2, 19]. |
| Modified version of Adolescent Pediatric   Pain Tool [17] | NR [18] | Probably suitable for paediatric populations. Original version has adequate psychometric properties as stated above. This modified version enlarged the diagram of the back to more clearly assess the location of reported back pain [18]. |
| **Pain Location** |  |  |
| Adolescent Pediatric Pain Tool [17] | NR [1] | Suitable for paediatric population with adequate psychometric properties including content, construct and criterion validity and reliability in clinical and nonclinical groups of children and adolescence aged 8-17 years [1, 2, 19]. |
| Modified version of Adolescent Pediatric Pain Tool   [17, 32] | NR [2, 18] | Probably suitable for paediatric populations. Original version has adequate psychometric properties as stated above. The modified versions either enlarged the diagram of the back to more clearly assess the location of reported back pain [18], or divided the diagram of the back into 10 segments to identify specific pain locations [2]. |
| Leeds Assessment of Neuropathic Symptoms and   Signs (S-LANSS) [33] | NR [14] | Suitable measure of neuropathic pain, commonly used in adults with good psychometric properties [33]. In children and adolescents evidence for high sensitivity but low specificity has been found [34]. |
| Peds-CHOIR self-report body map [35] | NR [7] | Suitable for paediatric population, with good psychometric properties as a comprehensive outcome measure [35]. The present paper utilises only the graphical body map portion of the survey, to assess pain location [7]. Evidence has suggested body maps to be an appropriate assessment of pain location in children over the age of 8 [36]. |
| Migraine health history form | NR [30] | Suitability unknown as psychometric properties not reported [30]. |
| Bespoke body outline diagram | NR [3] | Probably suitable, although instructions given to participants not provided [3]. |
| Navigate Pain app [37, 38] | NR [12] | Psychometric properties not reported so unknown if suitable for paediatric pain populations [12]. |
| **Pain Interference** |  |  |
| Child Activity Limitation Interview-21 [39] | NR [9] | Suitable for paediatric chronic pain populations, with evidence of good psychometric properties including internal consistency, test-retest reliable and concurrent validly [39]. |
| PROMIS Pediatric Pain Interference – 8-item   Short-form [40] | Internal consistency = .85 to .92 [5]  NR [7] | Suitable for paediatric populations with chronic and recurrent pain [40], with evidence of good psychometric properties including internal consistency [5]. |
| PROMIS Pediatric Pain Interference v 2.0 – 4-item   Short-form | NR [7] | Suitability unknown as psychometric properties of the 4-item version not found [7]. |
| Numerical Rating Scale | NR [14] | Suitable for paediatric populations when assessing pain intensity [13]. Further research required to examine psychometric properties in the assessment of pain interference. |
| **Neuropathic Pain** |  |  |
| Douleur Neuropathique 4 [41] | NR [1-3] | Potentially suitable. The DN4 has not been validated for use among children and adolescents. Despite low level evidence of satisfactory criterion and construct validity and reliability, it has been described as most suitable for clinical use [1, 42, 43]. |
| Leeds Assessment of Neuropathic Symptoms and   Signs [44] | NR [14] | Suitable measure of neuropathic pain, commonly used in adults, with good psychometric properties [44], but a cut off score has not been validated among adolescents [14]. |
| Neuropathic Pain Questionnaire [41] | NR [18] | Potentially suitable. The DN4 has not been validated for use among children and adolescents. Despite low level evidence of satisfactory criterion and construct validity and reliability, it has been described as most suitable for clinical use [1, 42, 43]. |
| **Abdominal Pain** |  |  |
| Abdominal Pain Index [45] | Internal consistency .79 to .92 [5]  NR [9] | Suitable for children with abdominal pain, with evidence for good internal consistency, test-re-test reliability, concurrent validity between patient and parental report, and discriminant validity [45]. |
| IBS Symptoms Diary (including NRS of pain   intensity and pain interference) | NR [4] | Probably suitable, although psychometric properties of version used unknown [4]. |
| **Neck Disability** |  |  |
| Neck Disability Index – Spanish version [46] | NR [10] | Suitable for young people, with evidence for good internal consistency and test-retest reliability [46]. |
| **Migraine History** |  |  |
| Bespoke form | NR [30] | Suitability unknown as psychometric properties not reported [30]. |
| **Pain History and Location** |  |  |
| Leubeck Pain Screening Questionnaire (LPQ) -   Portuguese version (parent- and patient-reported)   [47] | NR [15, 48] | Suitability unknown. Original version has satisfactory psychometric properties including feasibility, content, and face validity, before translating into Portuguese [47, 49, 50]. |
| **Headache Frequency** |  |  |
| Migraine health history form | NR [30] | Suitability unknown as psychometric properties not reported [30]. |
| **Gastrointestinal Symptoms** |  |  |
| Questionnaire on Paediatric Gastrointestinal Symptoms [51] | NR [52] | Probably suitable for paediatric populations with gastrointestinal symptoms, although psychometric properties of English version are difficult to find [51]. Within the present paper only sections A and B of the scale were used of which the precise psychometric properties are not reported [52]. |
| **Knee Injury and Osteoarthritis Outcomes (inc. quality of life)** |  |  |
| Knee Injury and Osteoarthritis Outcome Score [53] | NR [12, 54] | Probably suitable for adolescents and children. Measure demonstrates good psychometric properties with high test-retest reliability, evidence of validity, and was responsive to change, among an adult population [53]. Likely suitable for a young adult population [12], yet the psychometric properties of this measure among adolescents and children is unclear [54]. |
| **Central Sensitisation** |  |  |
| Central Sensitisation Inventory – Spanish version [55] | NR [10] | Suitable for young populations. Spanish version of this questionnaire has good psychometric properties including good internal consistency, test-retest reliability and construct validity [55]. |
| **Pain Intensity during conditioned pain modulation** |  |  |
| Numerical Rating Scale/Computerised Numerical Rating Scale | NR [1, 2, 4-7, 14, 25, 29, 52, 56-61] | Suitable for a paediatric population, assessing acute pain intensity in children as young as 6 years old with good psychometric properties including test-retest reliability, construct validity, and concurrent validity [13]. |
| Visual Analogue Scale/Computerised Visual Analogue Scale | NR [1, 3, 6, 8, 12, 15, 18, 30, 48, 54, 62-65] | Suitable for assessing pain intensity among children and adolescents aged between 6 and 17 years, with evidence of good psychometric properties including test-retest reliability and internal responsiveness [16]. Research with adults suggests no clinically relevant difference exists between the traditional paper-based assessment and scores obtained from laptop computer and mobile phone–based platforms [66]. |
| Faces Pain Scale – Revised [20] | NR [21] | Suitable for paediatric populations with and without chronic pain, with evidence of good psychometric properties including test-retest reliability, content, and construct validity [20, 22]. |
| Wong-Baker Faces Pain Rating Scale [23] | NR [61] | Suitable for a paediatric population with good psychometric properties including test-retest reliability, concurrent validity and some evidence of discriminant validity [22, 23]. |
| **Pain Unpleasantness during conditioned pain modulation** |  |  |
| Visual Analogue Scale | NR [6, 30, 64, 65] | Suitable for assessing pain unpleasantness [67] and suitable among children and adolescents aged between 6 and 17 years, with evidence of good psychometric properties including test-retest reliability and internal responsiveness [16]. |
| **Pain Sensations during conditioned pain modulation** |  |  |
| Agreement with descriptors (bespoke sensation   questionnaire) | NR [64] | Psychometric properties not reported [64]. |
| **Anxiety/Fear during conditioned pain modulation** |  |  |
| Numerical Rating Scale | NR [57] | Suitable for a paediatric population with evidence good psychometric properties when assessing pain intensity [13]. Psychometric properties precisely for anxiety and fear are unclear. |
| **Anticipatory Anxiety before conditioned pain modulation** |  |  |
| Numerical Rating Scale | NR [14, 29, 57] | Suitable for a paediatric population with evidence good psychometric when assessing pain intensity [13]. Psychometric properties for anticipatory anxiety specifically are unclear. |
| Visual analogue Scale | NR [63] | Suitable for assessing pain intensity among children and adolescents aged between 6 and 17 years, with evidence of good psychometric properties [16]. Psychometric properties for anticipatory anxiety specifically are unclear. |
| **Anxiety** |  |  |
| State-Trait Anxiety Inventory [68] | NR [56] | Potentially unsuitable for an adolescent mean age of 16. A modified version of the measure for a paediatric population has evidence of good psychometric properties including internal consistency and test-retest reliability [69]. |
| State subscale of the State-Trait Anxiety Inventory –   Spanish version [70] | NR [10] | Suitable for the young adult sample in the present paper [10]. The Spanish version of the STAI has good psychometric properties including test-retest reliability and internal consistency [70]. |
| Hospital Anxiety and Depression Scale [71] | NR [72] | Suitable for the sample of young adults within the present paper [72]. This test has evidence of good psychometric properties including adequate test-retest reliability and sensitivity [73]. |
| State anxiety subscale of the Profile of Mood States-  II (POMS-II) [74] | NR [60] | Suitable for young adult population within the present paper [60], psychometric properties are unclear for the anxiety subscale specifically. |
| Patient-Reported Outcomes Measurement   Information System (PROMIS) – Anxiety subscale   [75-77] | Coefficient alpha = .79 [58]  NR [30] | Suitable for paediatric population with evidence of acceptable psychometric properties including internal consistency [58]. |
| Revised Child Anxiety and Depression Scale [78] | NR [1-3, 18] | Suitable for a paediatric population with evidence of good psychometric properties including structural validity, reliability, and convergent and discriminant validity [78]. |
| Paediatric Index of Emotional Distress [79] | NR [14] | Suitable for a paediatric population, as a reliable and valid measure of emotional distress [79]. However psychometric properties are unclear in the assessment of anxiety exclusively. |
| Behavior Assessment System for Children – Second   Edition – child version [80] | NR [4] | Suitable for use with children and adolescents, with evidence for good internal consistency and test-retest reliability [80]. |
| Behavior Assessment System for Children – Second   Edition – parent version [80] | NR [4] | Suitable for use with children and adolescents, with evidence for good internal consistency and test-retest reliability [80]. |
| **Depression** |  |  |
| Children’s Depression Inventory [81] | Internal consistency > .73 [5]  NR [56, 59] | Suitable for a paediatric population with good psychometric properties including internal consistency [5], test-retest reliability and sensitivity [82]. |
| Hospital Anxiety and Depression Scale [71] | NR [72] | Suitable for the sample of young adults within the present paper [72]. This test has evidence of good psychometric properties including adequate test-retest reliability and sensitivity [73]. |
| Patient-Reported Outcomes Measurement Information System (PROMIS) – Depression subscale [75-77] | Coefficient alpha = .92 [58]  NR [30] | Suitable for paediatric populations with evidence of good psychometric properties including internal consistency [58]. |
| Revised Child Anxiety and Depression Scale [78] | NR [1-3] | Suitable for a paediatric population with evidence of good psychometric properties including structural validity, reliability, and convergent and discriminant validity [78]. |
| Paediatric Index of Emotional Distress [79] | NR [14] | Suitable for a paediatric population, as a reliable and valid measure of emotional distress [79]. However psychometric properties are unclear in the assessment of depression exclusively. |
| Beck Depression Inventory II - Spanish version [83] | NR [10] | Suitable for the sample of young adults included in the present paper [10], with evidence of good psychometric properties including internal consistency, and factorial validity [83]. |
| **Hopelessness** |  |  |
| Beck Hopelessness Scale [84] | NR [59] | Suitable for the population of young adults in the present paper [59], evidence of satisfactory psychometric properties in a nonclinical sample including internal consistency and convergent reliability [85]. |
| **Pain Catastrophising** |  |  |
| Pain Catastrophising Scale [86] | NR [56, 72] | Suitable for young adult sample [56], with evidence of good psychometric properties including internal reliability and test-retest reliability [87]. The suitability of this measure for an adolescent population is unclear, as a more suitable version exists for those aged 8 to 16 (Pain Catastrophizing Scale for children). |
| Pain Catastrophising Scale – Spanish version [88] | NR [10] | Suitable for young adult populations [10], with evidence of appropriate psychometric properties including internal consistency, test-rest reliability, and sensitivity to change amongst an adult pain population, with the same three-factor structure as the original English version [88]. |
| Pain Catastrophising Scale for Children [86, 89] | Coefficient alpha = .90 [58]  Cronbach’s alpha = .86 [29]  NR [1, 9, 14] | Suitable for paediatric populations with evidence of good psychometric properties including construct and predictive validity [89]. |
| Pain Response Inventory – Catastrophising Subscale [90] | Internal consistency > .73 [5] | Suitable for a paediatric population with evidence of good psychometric properties including internal consistency [5] and test-retest reliability [91]. |
| **Pain-Related Fear/Anxiety** |  |  |
| Dutch Fear of Pain Questionnaire – parent report [92] | NR [21] | The precise psychometric properties of the Dutch version utilised in the present study of the measure are unclear [21]. Original version is suitable for a paediatric population with a very strong internal consistency, construct validity [92]. |
| Child Pain Anxiety Symptoms Scale [93] | Cronbach’s alpha = .89 [29] | Suitable for a paediatric population with evidence of good psychometric properties including internal consistency and construct, discriminant and concurrent validity [93]. |
| **Pain Beliefs** |  |  |
| Pain Beliefs Questionnaire – Short-Form [94] | Internal consistency > .73 [5] | Suitable for adolescent populations with evidence for good internal consistency, test-retest reliability, concurrent validity and construct validity [94] |
| **Functioning/Disability** |  |  |
| Functional Disability Inventory [95, 96] | Coefficient alpha = .91 [58]  Coefficient alpha = .92 [52]  Internal consistency > .73 [5]  NR [1-3, 18, 30] | Suitable for use in paediatric pain populations, with evidence for excellent internal consistency and high test-retest reliability [96] discriminant validity and sensitivity to treatment [95]. |
| Dutch version Functional Disability Inventory – Parent proxy [96] | NR [21] | Probably suitable. Dutch translation performed by Lionbridge Translation and Localization Services and approved by original author. Psychometric properties for Dutch version not found though. |
| Pediatric Migraine Disability Assessment Scale [97, 98] | NR [6, 30, 65] | Suitable for use in paediatric migraine populations, with evidence for good internal consistency, test-retest reliability and criterion validity [97]. |
| Chronic Pain Grade Questionnaire/Graded Chronic Pain Scale – Spanish version [99] | NR [10] | Suitable for use in paediatric pain populations, with evidence for good internal consistency and test-retest reliability, and moderate convergent reliability [99]. |
| **Kinesiophobia** |  |  |
| Tampa scale for Kinesiophobia – Spanish version [100] | NR [10] | Suitable for young populations, with evidence for good internal consistency, stability and validity convergent and predictive validity [100]. |
| **Quality of Life** |  |  |
| Paediatric Quality of Life Inventory – Child [101] | NR [102] | Suitable for paediatric populations, with evidence for acceptable internal consistency, discriminant validity and construct validity [101]. |
| Paediatric Quality of Life – Parent [101] | NR [102] | Suitable for paediatric populations, with evidence for acceptable internal consistency, discriminant validity and construct validity [101]. |
| **Somatization/Somatic Symptoms** |  |  |
| Children’s Somatisation Inventory revised form [103] | Coefficient alpha = .84 [58]  Coefficient alpha = .93 [52] | Suitable for paediatric populations, with evidence for good internal consistency and a strong general factor [103]. |
| Children’s Somatization Inventory-35 – Child version [104] | NR [4] | Suitable for paediatric pain populations, with evidence for good internal consistency and concurrent and convergent validity [105]. |
| Children’s Somatization Inventory-35 – Parent version [104] | NR [4] | Suitable for paediatric pain populations, with evidence for good internal consistency [106]. |
| Children’s Somatic Symptom Inventory - gastrointestinal subscale [107] | Internal consistency .72 to .85 [5] | Suitable for paediatric chronic pain populations, with evidence for good internal consistency and a strong general factor [103]. |
| **Personality** |  |  |
| Personality Inventory for DSM-5 [108, 109] | NR [59] | Italian translation suitable for use in non-clinical populations, with evidence for adequate internal consistency (except for the suspiciousness subscale) and average inter-item correlation values [110]. |
| **Physical Activity** |  |  |
| Physical Activity Questionnaire [111] | NR [72] | Suitable for use in young populations, with evidence for good test-retest reliability and concurrent and criterion validity [111]. |
| Physical Activity Questionnaire – Elementary School and High School Versions [112] | NR [25] | Suitable for use in paediatric populations, with evidence for acceptable internal consistency and test-retest reliability [112]. |
| **Fatigue** |  |  |
| Checklist for Individual Strength [113] | NR [72] | Suitable for use in clinical and non-clinical young populations, with evidence for good to excellent internal consistency, acceptable to good test-reliability [113]. |
| **Sleep Quality** |  |  |
| Pittsburgh Sleep Quality Index [114] | NR [1-3, 18, 56, 72] | Suitable for paediatric populations, with evidence for acceptable internal consistency and support for external validity in children and adolescents with chronic pain [115], and acceptable internal consistency and convergent validity in community-based adolescents [116]. |
| **Self-Harm** |  |  |
| Deliberate Self-Harm Inventory [117] | NR [59] | Unsure if suitable for younger children, as the original scale was developed with university students aged 18-64 (mean age 23.19) [117]. Psychometric properties of Italian version unknown. |
| Repetitive Non-Suicidal Self-Injury Questionnaire [118] | NR [59] | Suitable for paediatric populations, with evidence for good construct validity and internal consistency [118]. Psychometric properties of Italian version unknown however. |
| Clinician rated Severity of non-suicidal injury [109] | NR [59] |  |
| **Bullying** |  |  |
| Bully Scale Survey [119] | NR [15] | Probably suitable. Unclear which measure this is precisely, although scale has shown good internal consistency in previous large-scale study [120]. |
| **Childhood adversity** |  |  |
| Adapted version of the Adverse Childhood Experiences [121] | NR [15] | Unsure if suitable, as scale has been adapted and psychometric properties not provided in original article [121]. |
| **Parent Outcomes** | | |
| **Maternal Fear of Pain & Pain Related Anxiety** |  |  |
| Pain Anxiety Symptoms Scale – 20-item version [122] | NR [57] | Unsuitable for mother’s assessing their own fear of pain and pain-related anxiety pain. Scale was originally developed as a self-report measure for patients with chronic pain, and items refer to the respondent’s own pain. Has good internal consistency, criterion validity and construct validity in chronic pain patients [122]. |
| **Maternal General Psychological Distress** |  |  |
| Brief symptom inventory – 18-item version, Global severity index [123] | NR [57] | Probably suitable for mother’s assessing their psychological distress. Scale was originally developed to be used with adult medical patients and individuals in the community not currently assigned patient status. Scale has good internal consistency, test-retest reliability based on data from community and non-patient samples [123]. |
| **Parental Pain Catastrophising** |  |  |
| Pain Catastrophising Scale: parent version [124] | NR [14] | Suitable for use in parents of children with chronic pain. Good support for the scale’s internal consistency, construct validity and criterion validity in Dutch language [124]. Unclear if modification to original Pain Catastrophising Scale was made before or after translating into Dutch. |
| Dutch Pain Catastrophising Scale: parent version   [124] | NR [21] | Suitable for use in parents of children with chronic pain. Good support for the scale’s internal consistency, construct validity and criterion validity in Dutch language [124]. Unclear if modification to original Pain Catastrophising Scale was made before or after translating into Dutch, and information on translation methods not provided. |

References

1. Ocay, D.D., et al., *Phenotyping Chronic Musculoskeletal Pain in Male and Female Adolescents: Psychosocial Profiles, Somatosensory Profiles and Pain Modulatory Profiles.* Journal of pain research, 2022. **15**: p. 591-612.

2. Ocay, D.D., et al., *Psychosocial and psychophysical assessment in paediatric patients and young adults with chronic back pain: A cluster analysis.* European journal of pain (London, England), 2022. **26**(4): p. 855-872.

3. Teles, A.R., et al., *Evidence of impaired pain modulation in adolescents with idiopathic scoliosis and chronic back pain.* The Spine Journal, 2019. **19**(4): p. 677-686.

4. Williams, A.E., et al., *Endogenous inhibition of somatic pain is impaired in girls with irritable bowel syndrome compared with healthy girls.* The Journal of Pain, 2013. **14**(9): p. 921-930.

5. Morris, M.C., et al., *Does Quantitative Sensory Testing Improve Prediction of Chronic Pain Trajectories? A Longitudinal Study of Youth With Functional Abdominal Pain Participating in a Randomized Controlled Trial of Cognitive Behavioral Treatment.* The Clinical journal of pain, 2021. **37**(9): p. 648-656.

6. Nahman-Averbuch, H., et al., *Identification of neural and psychophysical predictors of headache reduction after cognitive behavioral therapy in adolescents with migraine.* Pain, 2021. **162**(2): p. 372-381.

7. Li, R., et al., *Feasibility and reliability of a quantitative sensory testing protocol in youth with acute musculoskeletal pain postsurgery or postinjury.* Pain, 2023.

8. Ocay, D.D., et al., *Clusters of facilitatory and inhibitory conditioned pain modulation responses in a large sample of children, adolescents, and young adults with chronic pain.* Pain Reports, 2022. **7**(6).

9. Tham, S.W., et al., *Pain catastrophizing moderates the relationship between pain sensitivity and clinical pain in adolescents with functional abdominal pain.* The Journal of Pain, 2024.

10. Arribas‐Romano, A., et al., *Conditioned pain modulation and psychological factors in young adults with recurrent or chronic neck pain.* Pain Practice, 2024. **24**(3): p. 419-430.

11. Schubert-Hjalmarsson, E., et al., *Central sensitization in adolescents with hypermobility spectrum disorder or hypermobile Ehlers-Danlos syndrome-a feasibility study.* Pilot and feasibility studies, 2023. **9**(1): p. 97.

12. Holden, S., et al., *Young females with long-standing patellofemoral pain display impaired conditioned pain modulation, increased temporal summation of pain, and widespread hyperalgesia.* Pain, 2018. **159**(12): p. 2530-2537.

13. Castarlenas, E., et al., *Psychometric Properties of the Numerical Rating Scale to Assess Self-Reported Pain Intensity in Children and Adolescents: A Systematic Review.* The Clinical Journal of Pain, 2017. **33**(4): p. 376-383.

14. Verriotis, M., et al., *Phenotyping peripheral neuropathic pain in male and female adolescents: pain descriptors, somatosensory profiles, conditioned pain modulation, and child-parent reported disability.* Pain, 2021. **162**(6): p. 1732-1748.

15. Lucas, R., et al., *Bullying Involvement and Physical Pain Between Ages 10 and 13 Years: Reported History and Quantitative Sensory Testing in a Population-Based Cohort.* The journal of pain, 2024. **25**(4): p. 1012-1023.

16. Le May, S., et al., *Comparison of the psychometric properties of 3 pain scales used in the pediatric emergency department: Visual Analogue Scale, Faces Pain Scale-Revised, and Colour Analogue Scale.* Pain, 2018. **159**(8): p. 1508-1517.

17. Savedra, M.C., et al., *Assessment of postoperation pain in children and adolescents using the adolescent pediatric pain tool.* Nursing research, 1993. **42**(1): p. 5-9.

18. Ferland, C.E., et al., *Blood monoamines as potential biomarkers for conditioned pain modulation efficacy: an exploratory study in paediatrics.* European Journal of Pain, 2019. **23**(2): p. 327-340.

19. Jacob, E., et al., *Adolescent pediatric pain tool for multidimensional measurement of pain in children and adolescents.* Pain Manag Nurs, 2014. **15**(3): p. 694-706.

20. Hicks, C.L., et al., *The Faces Pain Scale–Revised: toward a common metric in pediatric pain measurement.* Pain, 2001. **93**(2): p. 173-183.

21. Pas, R., et al., *Endogenous pain modulation in children with functional abdominal pain disorders.* Pain, 2019. **160**(8): p. 1883-1890.

22. Stinson, J.N., et al., *Systematic review of the psychometric properties, interpretability and feasibility of self-report pain intensity measures for use in clinical trials in children and adolescents.* PAIN, 2006. **125**(1): p. 143-157.

23. Wong, D.L. and C.M. Baker, *Wong-Baker faces pain rating scale.* Pain Management Nursing, 2012.

24. Melzack, R., *The McGill Pain Questionnaire: major properties and scoring methods.* Pain, 1975. **1**(3): p. 277-299.

25. Stolzman, S. and M. Hoeger Bement, *Lean mass predicts conditioned pain modulation in adolescents across weight status.* European Journal of Pain, 2016. **20**(6): p. 967-976.

26. Main, C.J., *Pain assessment in context: a state of the science review of the McGill pain questionnaire 40 years on.* Pain, 2016. **157**(7): p. 1387-1399.

27. Melzack, R., *The short-form McGill pain questionnaire.* Pain, 1987. **30**: p. 191 - 197.

28. Landgraf, J.M., L. Abetz, and J.E. Ware, *Child Health Questionnaire (CHQ): A user's manual*. 1999: Landgraf & Ware.

29. Tsao, J.C., et al., *Conditioned pain modulation in children and adolescents: effects of sex and age.* The Journal of Pain, 2013. **14**(6): p. 558-567.

30. Nahman-Averbuch, H., et al., *Increased pain sensitivity but normal pain modulation in adolescents with migraine.* Pain, 2019. **160**(5): p. 1019-1028.

31. Pagé, M.G., et al., *Validation of the Numerical Rating Scale for Pain Intensity and Unpleasantness in Pediatric Acute Postoperative Pain: Sensitivity to Change Over Time.* The Journal of Pain, 2012. **13**(4): p. 359-369.

32. Fernandes, A.M., et al., *Pain assessment using the Adolescent Pediatric Pain Tool: a systematic review.* Pain Research and Management, 2014. **19**: p. 212-218.

33. Bennett, M.I., et al., *The S-LANSS score for identifying pain of predominantly neuropathic origin: validation for use in clinical and postal research.* The Journal of Pain, 2005. **6**(3): p. 149-158.

34. Walker, S.M., et al., *Sensitivity and specificity of a neuropathic screening tool (Self-Report Leeds Assessment of Neuropathic Symptoms and Signs, S-LANSS) in adolescents with moderate-severe chronic pain.* The Journal of Pain, 2024. **25**(2): p. 451-465.

35. Bhandari, R.P., et al., *Pediatric-Collaborative Health Outcomes Information Registry (Peds-CHOIR): a learning health system to guide pediatric pain research and treatment.* Pain, 2016. **157**(9): p. 2033.

36. von Baeyer, C.L., et al., *Pain charts (body maps or manikins) in assessment of the location of pediatric pain.* Pain Manag, 2011. **1**(1): p. 61-68.

37. Boudreau, S.A., et al. *Feature extraction APP for pain profiles*. in *Replace, Repair, Restore, Relieve–Bridging Clinical and Engineering Solutions in Neurorehabilitation: Proceedings of the 2nd International Conference on NeuroRehabilitation (ICNR2014), Aalborg, 24-26 June, 2014*. 2014. Springer.

38. Boudreau, S.A., et al., *Digital pain drawings: assessing touch-screen technology and 3D body schemas.* The Clinical journal of pain, 2016. **32**(2): p. 139-145.

39. Palermo, T.M., et al., *Development and validation of the Child Activity Limitations Interview: a measure of pain-related functional impairment in school-age children and adolescents.* Pain, 2004. **109**(3): p. 461-470.

40. Varni, J.W., et al., *PROMIS Pediatric Pain Interference Scale: an item response theory analysis of the pediatric pain item bank.* The Journal of Pain, 2010. **11**(11): p. 1109-1119.

41. Bouhassira, D., et al., *Comparison of pain syndromes associated with nervous or somatic lesions and development of a new neuropathic pain diagnostic questionnaire (DN4).* pain, 2005. **114**(1-2): p. 29-36.

42. de Leeuw, T.G., et al., *Diagnosis and Treatment of Chronic Neuropathic and Mixed Pain in Children and Adolescents: Results of a Survey Study amongst Practitioners.* Children, 2020. **7**(11): p. 208.

43. Mathieson, S., et al., *Neuropathic pain screening questionnaires have limited measurement properties. A systematic review.* Journal of Clinical Epidemiology, 2015. **68**(8): p. 957-966.

44. Bennett, M., *The LANSS Pain Scale: the Leeds assessment of neuropathic symptoms and signs.* Pain, 2001. **92**(1-2): p. 147-157.

45. Laird, K.T., et al., *Validation of the abdominal pain index using a revised scoring method.* Journal of pediatric psychology, 2015. **40**(5): p. 517-525.

46. JA, A.O. and D.M. AD, *Validation of a Spanish version of the Neck Disability Index.* Medicina Clinica, 2008. **130**(3): p. 85-89.

47. Roth-Isigkeit, A., et al., *Pain among children and adolescents: restrictions in daily living and triggering factors.* Pediatrics, 2005. **115**(2): p. e152-e162.

48. Brandão, M., et al., *Pain history and experimental pressure pain responses in adolescents: Results from a population‐based birth cohort.* European Journal of Pain, 2024. **28**(1): p. 70-82.

49. Haraldstad, K., et al., *Pain in children and adolescents: prevalence, impact on daily life, and parents’ perception, a school survey.* Scandinavian Journal of Caring Sciences, 2011. **25**(1): p. 27-36.

50. Roth-Isigkeit, A., et al., *[Pain in children and adolescents--results of an exploratory epidemiological study].* Schmerz, 2003. **17**(3): p. 171-8.

51. Walker, L.S., et al., *Recurrent abdominal pain: symptom subtypes based on the Rome II criteria for pediatric functional gastrointestinal disorders.* Journal of Pediatric Gastroenterology and Nutrition, 2004. **38**(2): p. 187-191.

52. Morris, M.C., et al., *Impaired conditioned pain modulation in youth with functional abdominal pain.* Pain, 2016. **157**(10): p. 2375.

53. Roos, E.M., et al., *Knee Injury and Osteoarthritis Outcome Score (KOOS)—development of a self-administered outcome measure.* Journal of Orthopaedic & Sports Physical Therapy, 1998. **28**(2): p. 88-96.

54. Holden, S., et al., *Mechanistic pain profiling in young adolescents with patellofemoral pain before and after treatment: a prospective cohort study.* Pain, 2020. **161**(5): p. 1065-1071.

55. Cuesta-Vargas, A.I., et al., *Cross-cultural adaptation and validity of the Spanish central sensitization inventory.* Springerplus, 2016. **5**: p. 1-8.

56. Chrétien, R., et al., *Reduced endogenous pain inhibition in adolescent girls with chronic pain.* Scandinavian Journal of Pain, 2018. **18**(4): p. 711-717.

57. Evans, S., et al., *Sex differences in the relationship between maternal fear of pain and children’s conditioned pain modulation.* Journal of pain research, 2013. **6**: p. 231.

58. Morris, M.C., et al., *Race effects on conditioned pain modulation in youth.* The Journal of Pain, 2015. **16**(9): p. 873-880.

59. Leone, C., et al., *Dissecting pain processing in adolescents with Non‐Suicidal Self Injury: Could suicide risk lurk among the electrodes?* European Journal of Pain, 2021. **25**(8): p. 1815-1828.

60. Ray, L.N. and P.J. O'Connor, *Session of yoga, with and without slow (Ujjayi) breathing, reduces anxiety; no change on acute pain sensitivity and endogenous pain modulation.* Explore (New York, N.Y.), 2023. **19**(3): p. 362-370.

61. Jørgensen, J.V., et al., *Assessment of somatosensory profiles by quantitative sensory testing in children and adolescents with and without cerebral palsy and chronic pain.* European journal of paediatric neurology : EJPN : official journal of the European Paediatric Neurology Society, 2024. **51**: p. 32-40.

62. Goffaux, P., et al., *Preterm births: can neonatal pain alter the development of endogenous gating systems?* European Journal of Pain, 2008. **12**(7): p. 945-951.

63. Hoehn, J.L., L.M. Dahlquist, and J.A. Zeroth, *Conditioned Pain Modulation in Children: The Effects of Painful and Nonpainful Conditioning Stimuli.* The journal of pain, 2022.

64. Harper, D. and M. Hollins, *Conditioned pain modulation dampens the thermal grill illusion.* European Journal of Pain, 2017. **21**(9): p. 1591-1601.

65. Nahman-Averbuch, H., et al., *Spatial aspects of pain modulation are not disrupted in adolescents with migraine.* Headache, 2021. **61**(3): p. 485-492.

66. Delgado, D.A., et al., *Validation of digital visual analog scale pain scoring with a traditional paper-based visual analog scale in adults.* JAAOS Global Research & Reviews, 2018. **2**(3): p. e088.

67. Duncan, G.H., M.C. Bushnell, and G.J. Lavigne, *Comparison of verbal and visual analogue scales for measuring the intensity and unpleasantness of experimental pain.* Pain, 1989. **37**(3): p. 295-303.

68. Spielberger, C.D., R.L. Gorsuch, and R.E. Lushene, *State Trait Anxiety Inventory*. 1970, Palo Alto, California: Consulting Psychologists Press.

69. Southam-Gerow, M.A., E.C. Flannery-Schroeder, and P.C. Kendall, *A psychometric evaluation of the parent report form of the State-Trait Anxiety Inventory for Children—Trait Version.* Journal of Anxiety Disorders, 2003. **17**(4): p. 427-446.

70. Spielberger, C., F. Gonzalez-Reigosa, and A. Martinez-Urrutia, *Development of the Spanish Edition Of The State-Trait Anxiety Invetory.* Interam J Psychol, 1971. **5**: p. 3-4.

71. Zigmond, A.S. and R.P. Snaith, *The Hospital Anxiety and Depression Scale.* Acta Psychiatrica Scandinavica, 1983. **67**(6): p. 361-370.

72. Uzawa, H., S. Takeuch, and Y. Nishida, *Sex differences in conditioned pain modulation effects and its associations with autonomic nervous system activities in healthy, younger individuals: a pilot study.* Pain reports, 2024. **9**(2): p. e1123-e1123.

73. White, D., et al., *Validation of the Hospital Anxiety and Depression Scale for use with adolescents.* Br J Psychiatry, 1999. **175**: p. 452-4.

74. Heuchert, J.P. and D.M. McNair, *Profile of Mood States, POMS-2*. 2012, North Tonawanda: Multi-Health Systems Inc.

75. Irwin, D.E., et al., *An item response analysis of the pediatric PROMIS anxiety and depressive symptoms scales.* Quality of Life Research, 2010. **19**(4): p. 595-607.

76. Cella, D., et al., *The Patient-Reported Outcomes Measurement Information System (PROMIS): progress of an NIH Roadmap cooperative group during its first two years.* Medical care, 2007. **45**(5 Suppl 1): p. S3.

77. Pilkonis, P.A., et al., *Item banks for measuring emotional distress from the Patient-Reported Outcomes Measurement Information System (PROMIS®): depression, anxiety, and anger.* Assessment, 2011. **18**(3): p. 263-283.

78. Chorpita, B.F., et al., *Assessment of symptoms of DSM-IV anxiety and depression in children: A revised child anxiety and depression scale.* Behaviour research and therapy, 2000. **38**(8): p. 835-855.

79. O’Connor, S., et al., *The development and evaluation of the paediatric index of emotional distress (PI-ED).* Social psychiatry and psychiatric epidemiology, 2016. **51**(1): p. 15-26.

80. Reynolds, C. and R. Kamphaus, *Manual for the behavior assessment system for children.* 2004, Circle Pines, MN AGS Publishing.

81. Kovacs, M., *Children's Depression Inventory*. 1992, North Tonawanda, N.Y: Multi-Health System.

82. Masip, A., et al., *Psychometric Properties of the Children's Depression Inventory in Community and Clinical Sample.* The Spanish journal of psychology, 2010. **13**: p. 990-9.

83. Sanz, J., A.L. Perdigón, and C. Vázquez, *Adaptación española del Inventario para la Depresión de Beck-II (BDI-II): 2. Propiedades psicométricas en población general.* Clínica y salud, 2003. **14**(3): p. 249-280.

84. Beck, A.T., G. Brown, and R.A. Steer, *Prediction of eventual suicide in psychiatric inpatients by clinical ratings of hopelessness.* Journal of consulting and clinical psychology, 1989. **57**(2): p. 309.

85. Steed, L., *Further Validity and Reliability Evidence for Beck Hopelessness Scale Scores in a Nonclinical Sample.* Educational and Psychological Measurement, 2001. **61**(2): p. 303-316.

86. Sullivan, M.J.L., S.R. Bishop, and J. Pivik, *The Pain Catastrophizing Scale: development and validation.* Psychological Assessment, 1995. **7**: p. 524 - 532.

87. Wheeler, C.H.B., A.C.C. Williams, and S.J. Morley, *Meta-analysis of the psychometric properties of the Pain Catastrophizing Scale and associations with participant characteristics.* Pain, 2019. **160**(9): p. 1946-1953.

88. Rodero, B., et al., *Validation of the Spanish version of the Pain Catastrophizing Scale in fibromyalgia.* Medicina Clínica, 2008. **131**(13): p. 487-492.

89. Crombez, G., et al., *The child version of the pain catastrophizing scale (PCS-C): a preliminary validation.* Pain, 2003. **104**(3): p. 639-646.

90. Walker, L.S., et al., *A typology of pain coping strategies in pediatric patients with chronic abdominal pain.* PAIN®, 2008. **137**(2): p. 266-275.

91. Walker, L., et al., *Development and Validation of the Pain Response Inventory for Children.* Psychological Assessment, 1997. **9**: p. 392-405.

92. Simons, L.E., et al., *The Fear of Pain Questionnaire (FOPQ): assessment of pain-related fear among children and adolescents with chronic pain.* The Journal of Pain, 2011. **12**(6): p. 677-686.

93. Pagé, M.G., et al., *Development and preliminary validation of the Child Pain Anxiety Symptoms Scale in a community sample.* Journal of pediatric psychology, 2010. **35**(10): p. 1071-1082.

94. Stone, A.L., et al., *Pediatric Pain Beliefs Questionnaire: Psychometric properties of the short form.* The Journal of Pain, 2016. **17**(9): p. 1036-1044.

95. Walker, L.S. and J.W. Greene, *The functional disability inventory: measuring a neglected dimension of child health status.* Journal of pediatric psychology, 1991. **16**(1): p. 39-58.

96. Claar, R.L. and L.S. Walker, *Functional assessment of pediatric pain patients: psychometric properties of the functional disability inventory.* Pain, 2006. **121**(1-2): p. 77-84.

97. Hershey, A., et al., *PedMIDAS: development of a questionnaire to assess disability of migraines in children.* Neurology, 2001. **57**(11): p. 2034-2039.

98. Hershey, A.D., et al., *Development of a patient-based grading scale for PedMIDAS.* Cephalalgia, 2004. **24**(10): p. 844-849.

99. Ferrer-Peña, R., et al., *Adaptation and validation of the Spanish version of the graded chronic pain scale.* Reumatología Clínica (English Edition), 2016. **12**(3): p. 130-138.

100. Gómez-Pérez, L., A.E. López-Martínez, and G.T. Ruiz-Párraga, *Psychometric properties of the Spanish version of the Tampa Scale for Kinesiophobia (TSK).* The journal of Pain, 2011. **12**(4): p. 425-435.

101. Varni, J.W., M. Seid, and C.A. Rode, *The PedsQL™: measurement model for the pediatric quality of life inventory.* Medical care, 1999: p. 126-139.

102. Ruan, J.-H., et al., *Midterm Outcomes After Open Arthrolysis for Posttraumatic Elbow Stiffness in Children and Adolescents.* Journal of pediatric orthopedics, 2021. **41**(3): p. e266-e271.

103. Walker, L.S., et al., *Children's Somatization Inventory: psychometric properties of the revised form (CSI-24).* Journal of pediatric psychology, 2009. **34**(4): p. 430-440.

104. Walker, L. and J. Garber, *Children’s somatization inventory: preliminary manual.* Nashville, TN: Vanderbilt University Medical Center, 1992.

105. Lavigne, J.V., M. Saps, and F.B. Bryant, *Reexamining the factor structure of somatization using the children's somatization inventory (CSI-24) in a community sample.* Journal of pediatric psychology, 2012. **37**(8): p. 914-924.

106. Walker, L.S., J. Garber, and J.W. Greene, *Psychosocial correlates of recurrent childhood pain: a comparison of pediatric patients with recurrent abdominal pain, organic illness, and psychiatric disorders.* Journal of abnormal psychology, 1993. **102**(2): p. 248.

107. Stone, A.L., et al., *Somatic symptoms in pediatric patients with chronic pain: Proposed clinical reference points for the children's somatic symptoms inventory (formerly the children's somatization inventory).* The Journal of Pain, 2019. **20**(8): p. 932-940.

108. Krueger, R.F., et al., *Initial construction of a maladaptive personality trait model and inventory for DSM-5.* Psychological medicine, 2012. **42**(9): p. 1879-1890.

109. Somma, A., et al., *DSM‐5 personality domains as correlates of non‐suicidal self‐injury severity in an Italian sample of adolescent inpatients with self‐destructive behaviour.* Personality and mental health, 2019. **13**(4): p. 205-214.

110. Somma, A., et al., *Reliability, factor structure, and associations with measures of problem relationship and behavior of the Personality Inventory for DSM-5 in a sample of Italian community-dwelling adolescents.* Journal of personality disorders, 2017. **31**(5): p. 624-646.

111. Craig, C.L., et al., *International physical activity questionnaire: 12-country reliability and validity.* Medicine & science in sports & exercise, 2003. **35**(8): p. 1381-1395.

112. Crocker, P., et al., *Measuring general levels of physical activity: preliminary evidence for the Physical Activity Questionnaire for Older Children.* Medicine and science in sports and exercise, 1997. **29**(10): p. 1344-1349.

113. Worm-Smeitink, M., et al., *The assessment of fatigue: Psychometric qualities and norms for the Checklist individual strength.* Journal of psychosomatic research, 2017. **98**: p. 40-46.

114. Buysse, D.J., et al., *The Pittsburgh Sleep Quality Index: a new instrument for psychiatric practice and research.* Psychiatry research, 1989. **28**(2): p. 193-213.

115. Larche, C.L., et al., *The Pittsburgh Sleep Quality Index: reliability, factor structure, and related clinical factors among children, adolescents, and young adults with chronic pain.* Sleep disorders, 2021. **2021**(1): p. 5546484.

116. Raniti, M.B., et al., *Factor structure and psychometric properties of the Pittsburgh Sleep Quality Index in community-based adolescents.* Sleep, 2018. **41**(6): p. zsy066.

117. Gratz, K.L., *Measurement of deliberate self-harm: Preliminary data on the Deliberate Self-Harm Inventory.* Journal of psychopathology and behavioral assessment, 2001. **23**(4): p. 253-263.

118. Manca, M., F. Presaghi, and R. Cerutti, *Clinical specificity of acute versus chronic self-injury: Measurement and evaluation of repetitive non-suicidal self-injury.* Psychiatry Research, 2014. **215**(1): p. 111-119.

119. Hamburger, M.E., K.C. Basile, and A.M. Vivolo, *Measuring bullying victimization, perpetration, and bystander experiences; a compendium of assessment tools.* 2011.

120. Silva-Rocha, N., et al., *Bullying involvement, family background, school life, and well-being feelings among adolescents.* Journal of Public Health, 2020. **28**: p. 481-489.

121. Felitti, V.J., et al., *Relationship of childhood abuse and household dysfunction to many of the leading causes of death in adults: The Adverse Childhood Experiences (ACE) Study.* American journal of preventive medicine, 1998. **14**(4): p. 245-258.

122. McCracken, L.M. and L. Dhingra, *A short version of the Pain Anxiety Symptoms Scale (PASS-20): preliminary development and validity.* Pain research and management, 2002. **7**(1): p. 45-50.

123. Derogatis, L.R., *Brief Symptom Inventory 18*. 2001, Minneapolis, Mnnesota: NCS Pearson INC.

124. Goubert, L., et al., *Parental catastrophizing about their child’s pain. The parent version of the Pain Catastrophizing Scale (PCS-P): a preliminary validation.* Pain, 2006. **123**(3): p. 254-263.
